# Depolymerized Poly(ethylene-2,5-furanoate) as a Sustainable Feedstock for Biobased Unsaturated Polyester Resins

**DOI:** 10.1021/acs.macromol.5c01600

**Published:** 2025-09-25

**Authors:** Tomáš Foltýn, Jan Všetečka, Roman Svoboda, Štěpán Podzimek, Jaromír Vinklárek, Jan Honzíček

**Affiliations:** † Institute of Chemistry and Technology of Macromolecular Materials, Faculty of Chemical Technology, 48252 University of Pardubice, Studentská 573, Pardubice 532 10, Czech Republic; ‡ Department of Physical Chemistry, Faculty of Chemical Technology, University of Pardubice, Studentská 573, Pardubice 532 10, Czech Republic; § Synpo Ltd, S. K. Neumanna 1316, Pardubice 532 07, Czech Republic; ∥ Department of General and Inorganic Chemistry, Faculty of Chemical Technology, University of Pardubice, Studentská 573, Pardubice 532 10, Czech Republic

## Abstract

This study describes the chemical upcycling of poly­(ethylene-2,5-furanoate) (PEF) waste into biobased thermosetting materials. High molar-mass PEF of intrinsic viscosity of 1.04 dL/g, serving as a model of postconsumer PEF, was synthesized by melt polymerization followed by solid-state polymerization. After characterization, PEF was depolymerized by glycolysis to give a mixture of hydroxy-terminated oligomers suitable as a raw material for the production of styrene-free unsaturated polyester resins. It was exemplified in a series of formulations that use biobased itaconic acid as a source of polyester unsaturation and biobased dimethyl itaconate as a reactive diluent. Mechanical and thermoanalytic testing of the cured formulations indicates that biobased materials produced in this way can serve as an equal substitute for fossil-based polyester resins currently used in the industry.

## Graphical abstract

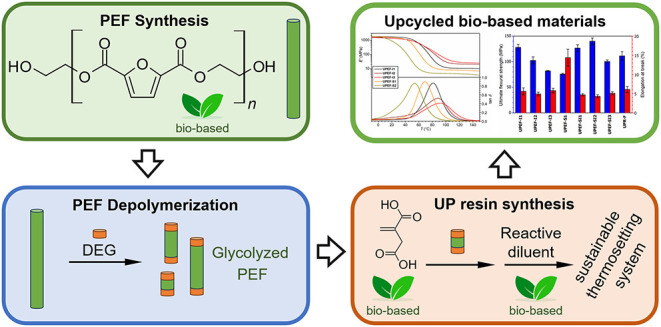

## Introduction

Poly­(ethylene-2,5-furanoate) (PEF) is one of the biobased thermoplastic polymers that can play a significant role in reducing greenhouse gas (GHG) emissions.^1^ Cradle-to-grave life-cycle assessment revealed that it can reduce GHG emissions by up to ∼54% relative to commercial fossil-based poly­(ethylene terephthalate) (PET).^2^ The superior mechanical,^3^ thermal,^4^ and gas barrier properties^5^ of PEF make it a sustainable alternative to PET for food and beverage packaging, including carbonated drinks. It allows thinner and more durable containers to be produced.^6^ Commercial use of PEF is still limited by the high market price of the key monomer, furan-2,5-dicarboxylic acid (FDCA), produced from carbohydrates.^7,8^ However, recent progress in FDCA technologies allows for an extension of its production and a considerable price drop.^9,10^ The most profitable commercialization scenario predicts that the market price of PEF will be ∼77% higher than that of fossil-based PET.^11^

Since the life cycle of thermoplastic polymers has become a global issue of utmost importance,^12^ it is necessary to consider waste valorization options for newly introduced polymers, including PEF. After its introduction to the market, PEF is expected to be mechanically recycled (molded and reshaped) in a mixture with PET, as the 5% PEF content in PET does not influence its mechanical properties. In later phases, the sorting is required by near-infrared equipment.^13^ It should be noted that the mechanical recycling of PEF is limited by the number of cycles. As in the case of other polyesters, it leads to decay in molar mass.^14,15^ Furthermore, PEF undergoes molar mass decay after UV irradiation, which can accelerate the degradation of outdoor items.^16^

Another option for the valorization of polyester waste is chemical recycling^17^ and biochemical recycling.^18^ These approaches seem to be very promising because they recover pure monomers and are not limited by the molar mass of the polymer to be recycled.^19^ Furthermore, they allow removal of used additives (e.g., colorants, plasticizers, and fillers) using common separation techniques (e.g., filtration and crystallization).^20^ The main drawback of chemical recycling processes is their high energetic demands, making the monomers produced more expensive than virgin monomers, which hampers their widespread application.^18^ Several technologies for the chemical recycling of PEF have been examined so far. They involve methanolysis catalyzed by ionic liquids^21^ or zinc acetate^22^ that gives dimethyl furan-2,5-dicarboxylate (DMFDC), which commonly serves as an intermediate in the synthesis of PEF from FDCA. The process is fast, but it proceeds at high temperatures and pressures. Mild conditions are reported for the saponification of PEF when methyl sesamol is used as a cosolvent.^23^ Biotechnological depolymerization of PEF by PET hydrolases represents another option for the recovery of FDCA or hydroxyethyl furan-2,5-dicarboxylate.^24,25^ However, this process is still slow, and the isolation of FDCA from a diluted aqueous solution would be challenging. So far, glycolysis of PEF using an acid–base organocatalyst^26^ or the deep eutectic solvent system^27^ seems to be the most promising avenue for chemical recycling of PEF, as it allows repolymerization without the need for catalyst removal.

The products of chemical depolymerization of polyesters can be used not only for their recycling but also as a feedstock in the chemical industry.^17,28^ Saving natural resources in this way aligns with the concept of upcycling when the final product is of a higher value.^29,30^ In the case of PEF, this approach was used for the preparation of high-performance fibers by partial substitution of ethylene glycol units with isosorbide.^30^ However, wider scrutiny of the upcycling concept for PEF is still lacking, which opens a gap for this study.

Note that chemical recycling and upcycling of plastic waste currently face multiple challenges in terms of scalability, pretreatment of raw materials, postprocessing steps, energy consumption, stability of catalysts, and equipment requirements, making this a pressing research topic.^31^ Although several technologies for commodity polyesters have been verified to be scalable with promising economic potential, substantial efforts are still required to establish a viable economic model and achieve industrial implementation.^32^

This study is focused on the development of new thermosetting systems accessible from PEF waste. For this purpose, the PEF was synthesized in bottle-grade quality, characterized, and then depolymerized by glycolysis. The formed hydroxy-terminated oligomers were then used as feedstocks for sustainable styrene-free polyester resins.

## Results and Discussion

### Synthesis of Bottle-Grade PEF

As PEF is still a polymer that is scarcely commercially accessible, it was synthesized on a ∼100 g scale by melt condensation polymerization of dimethyl furan-2,5-dicarboxylate (DMFDC) with ethylene glycol (EG) followed by solid-state condensation polymerization. The starting DMFDC was prepared from commercial FDCA in 76% yield by Fisher esterification and purified by recrystallization from ethyl acetate.

The melt polymerization process, carried out at *t* = 250 °C and *p* = 10 Pa, was followed by ^1^H NMR spectroscopy. Already at low conversion, solution in a mixture of CDCl_3_/CF_3_COOD shows well-resolved signals of the terminal EG (a1) and penultimate EG groups (a2) as well as the furane ring between them (b1), diethylene glycol (DEG) segments (c), and terminal COOCH_3_ functions (d); see Figure 1. The traces of terminal COOH are evidenced by the quartet at 7.38 ppm, assigned to the neighboring furane ring (see b3 in the magnified part of the spectrum in Figure S1 in the Supporting Information). We note that Soxhlet extraction with MeOH allows us to isolate oligomers with *M_n_* ∼ 500 g/mol, as determined by analysis of the end groups by ^1^H NMR spectroscopy (see Figure S2 in the Supporting Information).

**Figure 1:**
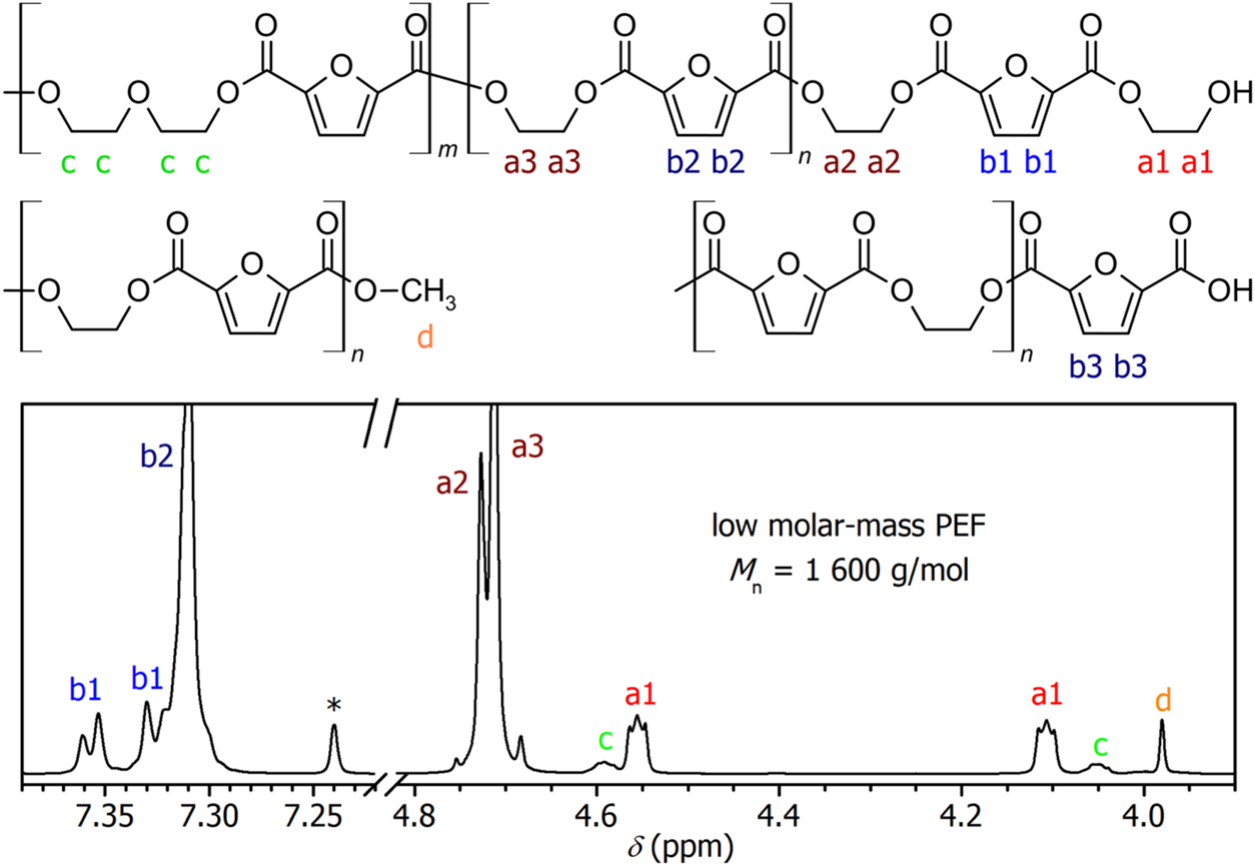
Assignment of signals in the ^1^H NMR spectrum on PEF with low molar mass (*M_n_* = 1600 g/mol).

The final product of the melt polymerization, obtained after 6 h, was washed with MeOH and tetrachloroethylene and then precipitated from HFIP (hexafluoropropan-2-ol)/CH_2_Cl_2_ solution by MeOH. Number-average molar mass (*M_n_* = 23,000 g/mol) and DEG content (4.1%) were calculated from the ^1^H NMR spectrum (Figure 2). To eliminate the previously reported esterification of end OH functions by trifluoroacetic acid,^33,34^ samples for the analysis of the aliphatic region were dissolved in a 20/1 mixture (*v*/*v*) of 1,1,2,2-tetrachloroethane-*d*_2_/phenol (Figure 2). Note that the obtained spectra exhibit better resolution of the DEG and EG signals than the solutions in the more convenient mixtures of CDCl_3_/CF_3_COOD, which were used for the analysis of the aromatic region (Figure S3 in the Supporting Information). The high *M_n_* value estimated from the NMR data is in line with the high intrinsic viscosity of the sample ([η] = 0.62 dL/g). Nevertheless, it is higher than the one calculated using the Mark–Houwink equation and constants determined by Berkowitz for PET (*M*_*n*;Berkowitz_ = 15,000 g/mol),^35^ which are commonly used for PEF.^36^

**Figure 2:**
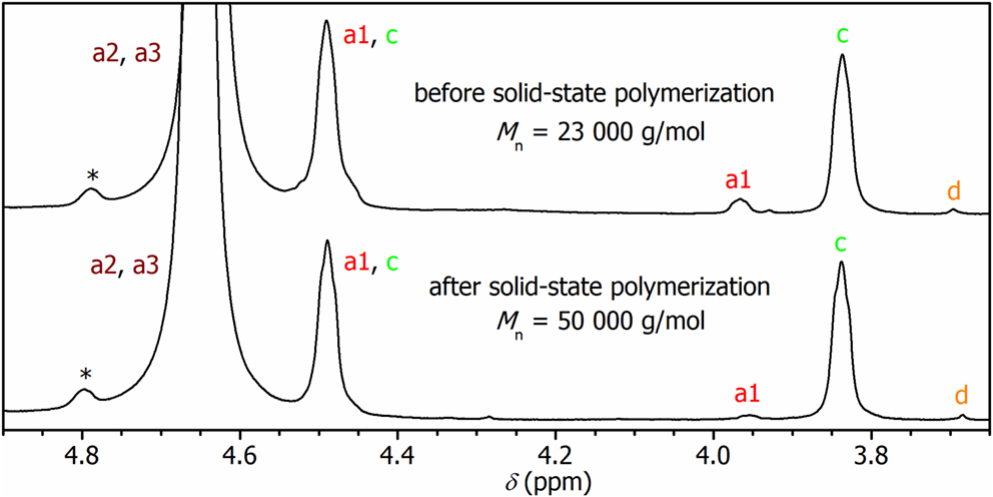
^1^H NMR spectra of medium-mass PEF (top) and high-mass PEF (bottom). Assignment is given in Figure 1. * ^13^C satellite signal from a3 segments.

Subsequent solid-state polymerization, performed at *t* = 200 °C and *p* = 10 Pa, led to a considerable increase in intrinsic viscosity ([η] = 1.04 dL/g) and *M_n_* value determined by NMR analysis of the end groups (*M_n_* = 50,000 g/mol). The aliphatic region of the ^1^H NMR spectrum, shown in Figure 2, documents a significant decrease in the content of terminal CH_2_CH_2_OH groups (signal a1). The decrease in the DEG content to 3.8% is ascribed to thermal degradation under conditions of the solid-state polymerization process, previously proposed for the PET polymer.^37^

The DSC data of PEF, synthesized by the solid-state polymerization process (Figure 3), confirm an increased polymerization degree as evidenced by an increase in the *T*_m_ value (from 207 to 209 °C). Increased *T*_g,DSC_ value (from 80 to 84 °C) indicates limited chain mobility because of a higher molar mass and potentially lower chain-end concentration.^36^ Furthermore, the major decrease in the enthalpic signal associated with melting (4.4 → 0.3 J/g) implies a much slower crystallization proceeding to a practically negligible extent compared with the sample synthesized by melt polymerization. This is ascribed to the formation of even longer and more interconnected polymeric chains with hindered reorganization. Note that neither the melt-polymerized PEF exhibits a considerable amount of crystallinity, as 4.4 J/g still represents only ∼3% of the polymer being in the crystalline form (the standard melting enthalpy of PEF in β form is estimated to be 137–140 J/g).^38,39^

**Figure 3:**
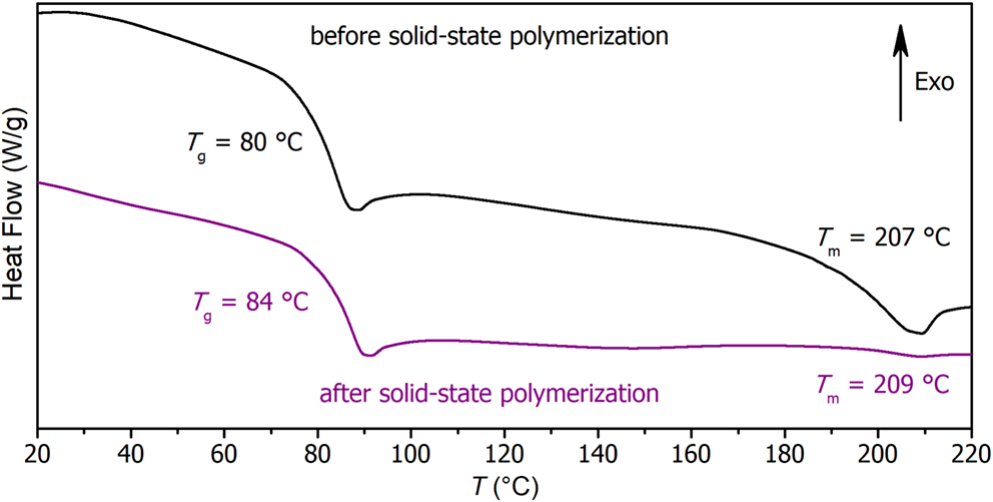
DSC curves (second heat, rate 20 °C/min) of medium-molar mass PEF (top) and high-molar mass PEF (bottom).

### Glycolysis of PEF

The high molar-mass PEF (*M_n_* = 50,000 g/mol) was depolymerized with one molar equivalent of DEG (relative to PEF repeating unit; *M*_r_ = 182.13) for 4 h. DEG was chosen for its high boiling point (245 °C), as it allows the glycolysis process to run at normal pressure even at temperatures higher than 200 °C. Glycolyzed PEF (**GLF**) was characterized by ^1^H NMR spectroscopy. It verified a statistical distribution of ester functions among OH groups of diols (Figure 4) without signs of DEG oligomerization, previously observed for PET glycolysis at elevated temperatures.^40^ Note that the samples for NMR spectroscopy were dissolved in two solvents (CDCl_3_ and DMSO-*d* ^6^) to achieve the full assignment (see Figure S8 in Supporting Information). For quantitative analysis, the DMSO-*d* ^6^ solutions were chosen due to better signal separation. Furthermore, the spectra showed well-resolved hydroxyl functions due to a strong hydrogen bonding with the sulfoxide function. Note that the oligomeric character of **GLF** was confirmed by SEC chromatography (Figure S9 in the Supporting Information).

**Figure 4:**
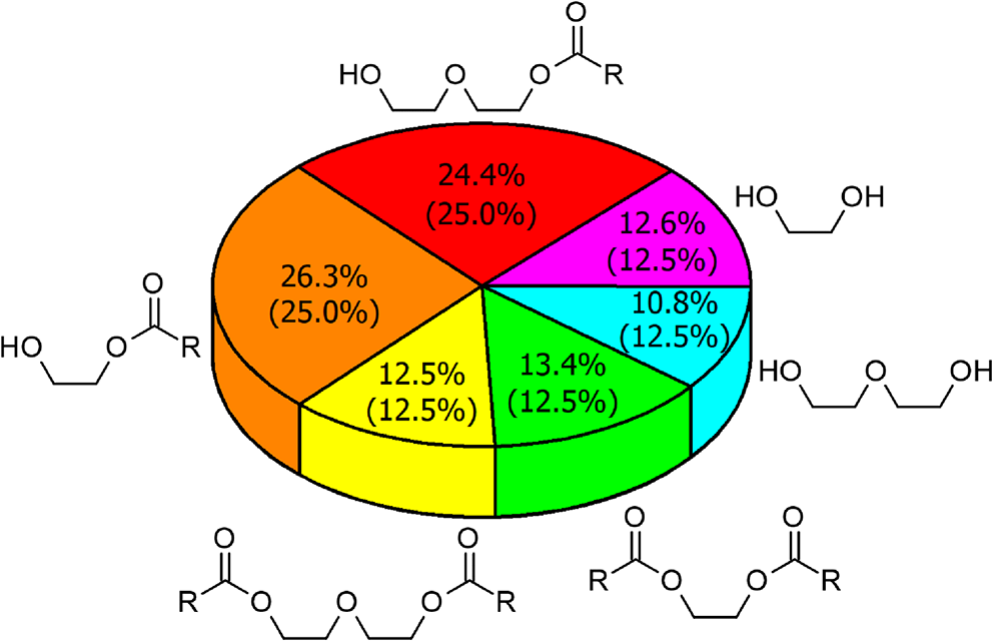
Composition of **GLF** was determined by ^1^H NMR spectroscopy. Theoretical value given in parentheses.

### Synthesis of PEF-Based Unsaturated Polyesters (UPs)

Three series of unsaturated polyesters were synthesized by the condensation polymerization of **GLF** with itaconic acid (IA), which serves as the source of unsaturation. They are denoted **UPEF-I**, **UPEF-S**, and **UPEF-SI**, and their feed composition is detailed in Table 1 together with experimental values determined by ^1^H NMR spectroscopy (values given in parentheses). The small differences in the actual chemical composition from that in the feed imply a negligible loss of the starting material during the polymerization process and an acceptable degree of undesired side reactions (Scheme 1). The condensation polymerization was carried out at a temperature not exceeding 160 °C to avoid extensive isomerization of IA to mesaconate (MES), as it reduces the number of reactive double bonds available for radical curing reactions.^41^ Higher temperatures also promote the formation of Ordelt adducts (OA) by the addition of glycols to the C=C double bond of itaconate, which leads to undesired polyester branching.^42^

**Table 1:** Feed and Actual Chemical Composition of Synthesized UPs^a^,^b^,^c^

polyester	FDCA	IA	SA	EG	DEG
**UPEF-I1**	0.25 (0.24)	0.25 (0.20)		0.25 (0.26)	0.25 (0.26)
**UPEF-I2**	0.20 (0.19)	0.30 (0.26)		0.20 (0.2)	0.30 (0.32)
**UPEF-I3**	0.15 (0.14)	0.35 (0.31)		0.15 (0.15)	0.35 (0.37)
**UPEF-S1**	0.20 (0.18)	0.20 (0.18)	0.10 (0.10)	0.20 (0.20)	0.30 (0.31)
**UPEF-S2**	0.15 (0.13)	0.15 (0.14)	0.20 (0.20)	0.15 (0.15)	0.35 (0.36)
**UPEF-SI1**	0.225 (0.22)	0.25 (0.20)	0.025 (0.03)	0.225 (0.23)	0.275 (0.30)
**UPEF-SI2**	0.20 (0.19)	0.25 (0.23)	0.05 (0.05)	0.20 (0.21)	0.30 (0.30)
**UPEF-SI3**	0.15 (0.15)	0.25 (0.23)	0.10 (0.11)	0.15 (0.14)	0.35 (0.34)

a Molar content of used building blocks.

b Feed composition; composition determined by ^1^H NMR is given in parentheses.

c Full composition including mesaconate and Ordelt adducts is given in Table S1 in Supporting Information.

**Scheme 1:**
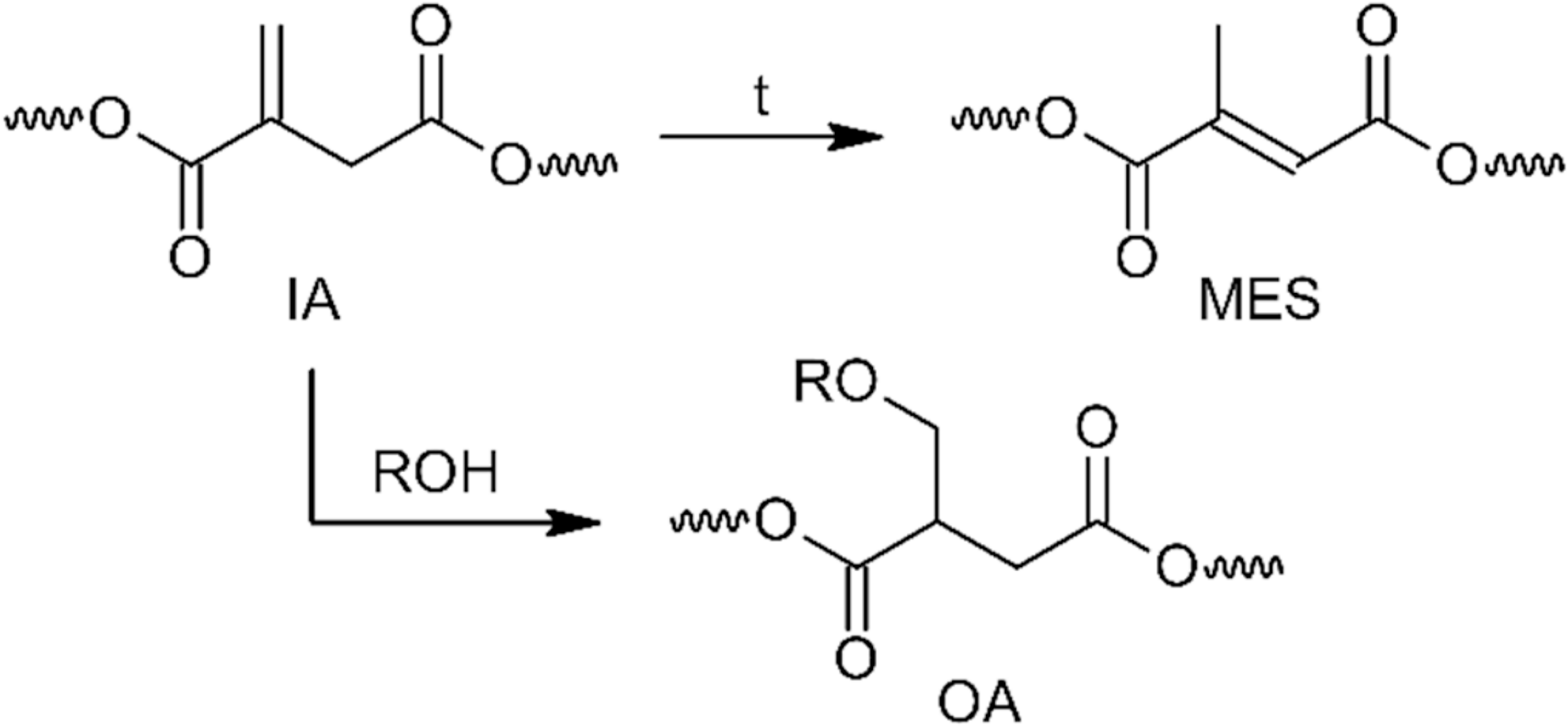
Side Products of the Condensation Polymerization^a^

a Isomerization of itaconate (top) and Ordelt addition (bottom).

The acid values of the final products do not exceed 50 mg of KOH/g (Table 2). As a reference, classical fumarate polyester (**UPR-F**) was synthesized from phthalic anhydride, maleic anhydride, ethylene glycol, and propylene glycol in a molar ratio 1:1:1:1. In this case, condensation polymerization was run at a higher temperature (240 °C) to allow extensive *cis*-*trans* isomerization of maleic acid to fumarate, which is crucial for enhancing the reactivity of the C=C double bond. It allows achieving a better copolymer with styrene, which is a convenient reactive diluent for **UPR-F**, due to the more favorable reactivity ratios (*r*_styrene_ = 0.301; *r*_fumarate_ = 0.0697) than in the maleate case (*r*_styrene_ = 6.52; *r*_fumarate_ < 0.01).^43^

**Table 2:** Properties of Synthesized UPs

polyester	AV (mg KOH/g)^a^	*M_n_* (g/mol)^b^,^c^	*Đ* ^b^,^c^
**UPEF-I1**	46	6200 (3200)	2.0 (3.0)
**UPEF-I2**	46	4700 (2500)	3.0 (3.0)
**UPEF-I3**	45	3700 (2500)	4.5 (3.7)
**UPEF-S1**	48	2300 (1900)	2.8 (2.1)
**UPEF-S2**	42	3100 (2300)	2.2 (2.1)
**UPEF-SI1**	32	4600 (2200)	2.6 (2.4)
**UPEF-SI2**	44	2300 (2200)	1.9 (1.9)
**UPEF-SI3**	49	2000 (2000)	2.1 (1.9)
**UPR-F**	28	4100 (3000)	6.4 (4.7)

a Acid values.

b Number-average molar mass (*M_n_*) and dispersity (*Đ*) determined by SEC-MALS;

c Values determined by SEC-RI on polystyrene standards are given in parentheses.

The absolute number-average molar mass (*M_n_*) and dispersity (*Đ*) of the itaconate polyesters (**UPEF** series) were determined by size-excluded chromatography (SEC) coupled with a multiangle light scattering (MALS) photometer (Table 2). The *M_n_* values range between 2100 and 6600 g/mol, demonstrating the formation of polyester chains long enough for thermosetting applications. Note that the conventional SEC technique using a refractive index (RI) detector with calibration on polystyrene standards gave considerably lower *M_n_* values (1900–3200 g/mol) due to the much higher polarity of polyester chains compared to polystyrene standards. Nevertheless, even these values are satisfactory for common thermosets and comparable industrial products (*M_n_* = 2000–4000 g/mol).^44^ The dispersity of most of the synthesized polyesters in the **UPEF** series ranges between 1.9 and 3.0. A greater deviation from a Flory distribution of molecular weights was observed only for polyester **UPEF-I3** (*Đ* = 4.5), which is ascribed to a higher degree of branching and rationalized by more extensive Ordelt additions due to a significantly higher IA content. An even higher value was observed for fumarate polyester **UPR-F** (*Đ* = 6.4). In this case, the higher degree of polyester branching is attributed to a much higher reaction temperature.^45^

In the parent polyester **UPEF-I1**, the **GLF** was treated with one molar equivalent of IA (calculated on the feed content of DEG), which gives a polyester with equal contents of the FDCA, IA, EG, and DEG segments (see Table 1). To explore the effect of unsaturation, polyesters **UPEF-I2** and **UPEF-I3** were synthesized with an increasing content of IA. In both cases, the lower content of hydroxy functions was compensated for by DEG.

Infrared and Raman spectra of the **UPET-I** series show a typical polyester pattern with a strong C=O stretching band at 1713 and 1734 cm^–1^, respectively (Figure 5). Broadening of the bands is caused by a different nature of ester functions in the polymer backbone (α,β-unsaturated, β,γ-unsaturated, aromatic). Comparison of the Raman bands in the **UPET-I** series reveals an increasing intensity of the shoulder at ∼1715 cm^–1^ with increasing IA content. This shoulder is assigned to the symmetrical C=O stretching mode of α,β-unsaturated IA moiety, as conjugation with the C=C double bond significantly reduces its vibration energy.

**Figure 5:**
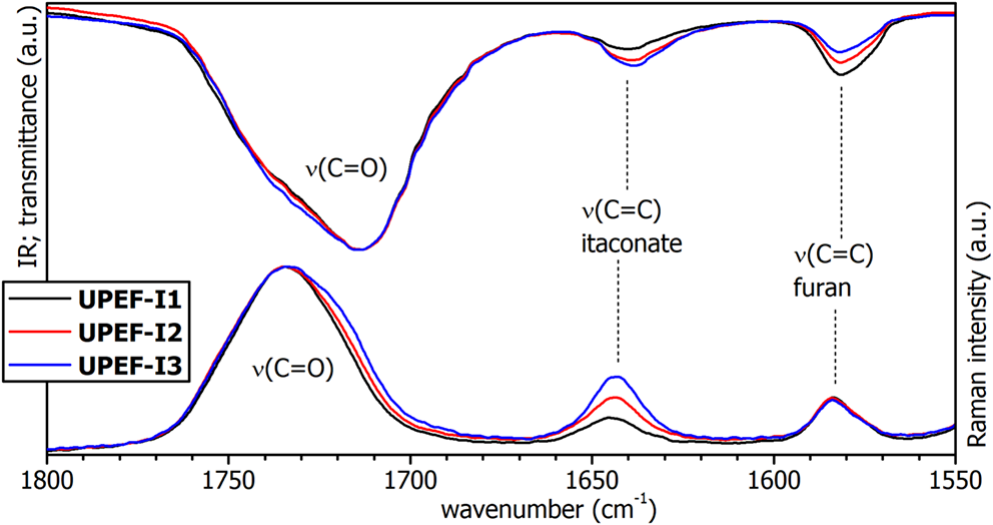
Infrared spectra (top) and Raman spectra (bottom) of the unsaturated polyesters **UPEF-I1** (black), **UPEF-I2** (red), and **UPEF-I3** (blue).

The infrared band at 1582 cm^–1^ was assigned to the antisymmetric C=C stretching of the furane ring, as its intensity correlates well with the FDCA content, decreasing in the order **UPET-I1** > **UPET-I2** > **UPET-I3**. The low wavenumber of the vibration mode is attributed to the aromatic character of the furane ring. The Raman band at 1644 cm^–1^, assigned to C=C stretching, is diagnostic for IA segments. The band increases in intensity in order **UPET-I1** < **UPET-I2** < **UPET-I3**, as the IA content rises.

^1^H NMR spectra were used for the quantitative analysis of the UP composition. The assignment of NMR signals to the UP segments is shown in Figure 6. The contents of the main building blocks, given in Table 1, agree well with the composition of the feed. Somewhat lower IA content is ascribed to partial isomerization to less reactive mesaconate (MES) and branching through Ordelt addition of the hydroxyl function group (OA) on the C=C double bond (Scheme 1).^42,46^ The appearance of both side products was evidenced by characteristic signals at 6.78 ppm (MES), 3.92 ppm (OA), and 3.56 ppm (OA). However, their content in the UP does not exceed 3% (MES) and 2% (OA), verifying a negligible reduction of the active C=C double bonds in the polyester backbone, which implies only a limited effect on the mechanical properties of the final thermosetting product. Note that the full compositions of UPs, including MES and OA, are given in Table S1 in the Supporting Information.

**Figure 6:**
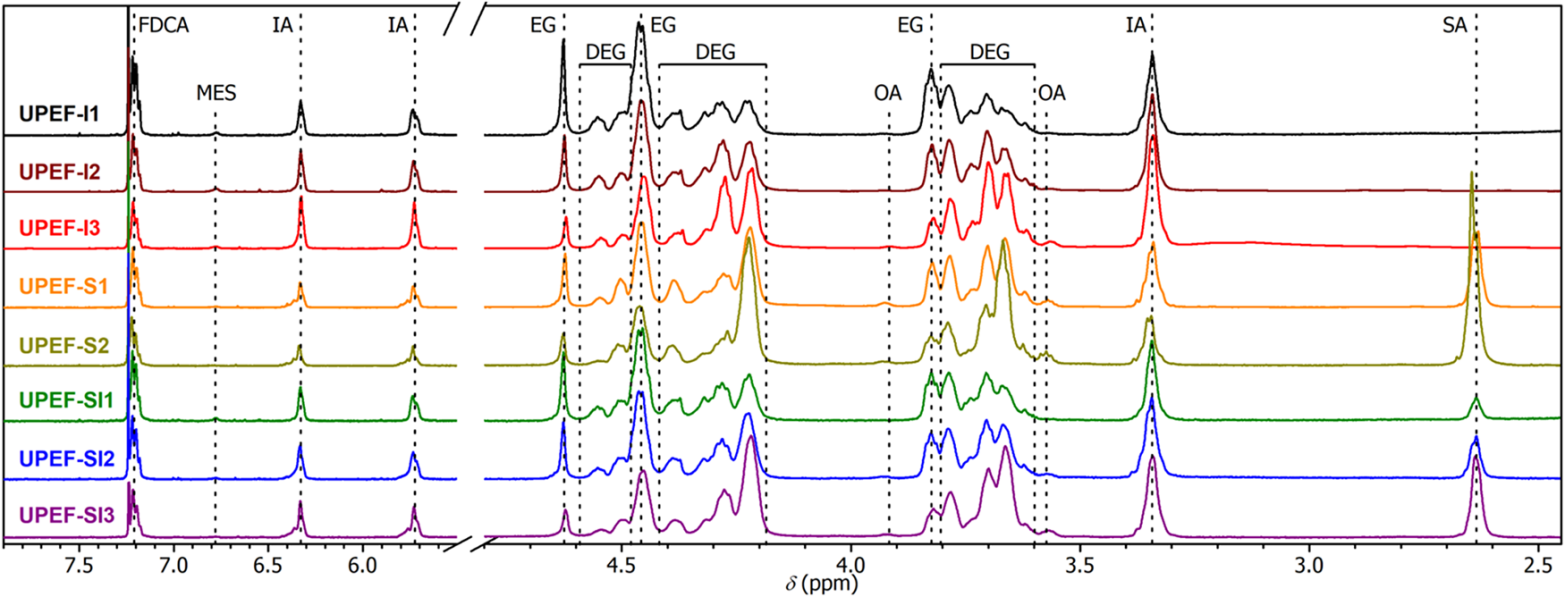
^1^H NMR spectra of UPs assigned to building blocks.

The **UPEF-S** series was designed to be less reactive than the parent compound **UPEF-I1**. The polyester backbone is plasticized by succinic acid (SA) and a higher content of DEG segments. The FDCA/IA ratio is adjusted to 1:1, as in the parent **UPEF-I1**. Plasticization by SA was also used in the **UPEF-SI** series. However, in this case, the content of IA is adjusted to 25%, which should ensure reactivity similar to that of **UPEF-I1**. The presence of SA segments in the polyesters of the **UPEF-S** and **UPEF-SI** series was evidenced by ^1^H NMR spectroscopy. They give a well-separated signal of methylene groups at 2.64 ppm.

### Curing of UP Formulations

In the present work, dimethyl itaconate (DMI), produced from sustainable feedstock, was used as a reactive diluent instead of the commonly used petrochemical styrene. Furthermore, the DMI diluent is nonvolatile and nonflammable and has a more convenient toxicological profile.^47^ Formulations containing 40 wt % of DMI were cured using a redox initiating system consisting of butanone peroxide (MEKP) and oxidovanadium­(IV) dibutylphosphate (VOP), which was developed for styrene-based UP formulations^48^ and recently shown to be very suitable for styrene-free systems.^47^ Two styrene-based references were used in this study: formulation of **UPR-F** (60 wt %) in styrene (40 wt %) and commercial multipurpose phthalate resin **PES109** of the same styrene content. Due to the higher reactivity of styrene, **UPR-F** was cured by a lower dose of MEKP and VOP. Note that **PES109** was cured by the MEKP/cobalt carboxylate system recommended by the supplier.

### Green Metrics of UPEF Formulations

The sustainability of the PEF processing to **UPEF** resins, atom economy (AE), and environmental factor (E-factor) were calculated for the protocols presented here (Tables S2–S19 in Supporting Information). These fundamental green chemistry metrics are valuable tools for quantifying both the theoretical proportion of raw materials incorporated into the final product and the actual amount of waste generated.^49^ PEF glycolysis is a waste-free process (AE = 100%; E-factor = 0) since no side products are formed, and the resulting **GLF** is used directly in the subsequent step without any purification. The theoretical efficiency of the condensation polymerization ranges from 88.3 to 91.4%, which is comparable to conventional polyester **UPR-F** (AE = 90.6%). Higher values are obtained for glycolysate-rich polyesters (e.g., **UPEF-I1**), due to the oligomeric nature of **GLF**. The AE parameter could be further improved to ∼95% by using itaconic anhydride and succinic anhydride instead of the corresponding acids, as this substitution halves the amount of water generated. In our setup, the procedure generates only a small amount of waste (E-factor = 0.41–0.46), consisting of esterification water and toluene used for its azeotropic removal. Since the solvent can be readily recovered without purification, the E-factor could be reduced to 0.1–0.13. The lower E-factor of **UPR-F** is attributed to a larger scale protocol with a reduced amount of solvent. Note that final dilution with a reactive diluent and the curing process generate no waste or side products (AE = 100%; E-factor = 0).

### Properties of Cured UP Formulations

Most of the cured formulations of the **UPEF** series have a gel content exceeding 95%, which is in line with the literature data for related itaconate-based resins.^40,50^ It indicates the formation of a highly cross-linked polyester network suitable for common thermosetting applications (Table 3). A lower value was observed only for the formulation **UPEF-S2**, which is attributed to a low IA content that results in a too high content of saturated polyester chains. Such interpretation is supported by the density cross-links (ν_e_) calculated from the DMA data (Figure 7), according to eq 1:

1 $$\nu_{e}=G^{\prime }/\mathrm{RT}$$

where *G*′ is the storage modulus in the rubbery plateau (at *T* = *T*_g,DMA_ + 50 K), *R* is the universal gas constant, and *T* is the absolute temperature. The acquired DMA data indicate the decrease of ν_e_ in line: **UPEF-I3** > **UPEF-I2** > **UPEF-SI** series ≈ **UPEF-I1** > **UPEF-S1** > **UPEF-SI2**, which follows the decrease in content of the IA segments in the polyester backbone. References formulations **UPR-F** and **PES109** exhibit ν_e_ comparable to **UPEF-I2** and **UPEF-I3**, respectively. Low-temperature shoulder at damping factor curves (tan δ) of reference formulations is attributed to segregation of low-polar polystyrene domains (Figure 7).

**Table 3:** Properties of Cured UPs Formulations

polyester resin	Gel^a^ (wt %)	*T*_g,DMA_ ^b^ (°C)	ν_e_ ^c^ (mmol/cm^3^)	σ_f,max_ ^d^ (MPa)	*E*_f_ ^e^ (GPa)	*ε*_f,failure_ ^f^ (%)	*T*_g,DSC_ ^g^ (°C)	*T*_5%,N2_ ^h^ (°C)	*T*_5%,air_ ^i^ (°C)
**UPEF-I1**	98.5	82.5	3.1	128.1 ± 5.8	3.2 ± 0.2	5.6 ± 0.9	57.8	230.3	231.7
**UPEF-I2**	99.5	88.4	6.1	102.6 ± 6.9	2.9 ± 0.2	4.9 ± 0.5	60.0	236.1	226.2
**UPEF-I3**	96.4	92.1	7.4	82.0 ± 0.9	2.3 ± 0.2	5.8 ± 0.6	62.9	238.9	213.8
**UPEF-S1**	99.8	69.2	2.4	75.8 ± 1.7	2.1 ± 0.1	14.4 ± 2.2	45.8	240.5	231.7
**UPEF-S2**	90.6	53.9	1.4	n.d.	n.d.	n.d.	41.7	236.0	211.5
**UPEF-SI1**	99.9	87.2	4.0	126.7 ± 6.5	3.2 ± 0.3	4.7 ± 0.3	54.5	270.6	251.8
**UPEF-SI2**	99.8	83.0	4.9	140.0 ± 6.4	3.8 ± 0.2	4.3 ± 0.4	50.8	255.7	238.6
**UPEF-SI3**	99.9	78.7	3.9	100.5 ± 3.0	3.0 ± 0.2	5.1 ± 0.4	46.6	242.3	234.7
**UPR-F**	95.3	100.1	5.9	111.3 ± 8.3	3.0 ± 0.2	6.1 ± 0.8	49.1	193.0	185.6
**PES109**	98.8	104.1	7.4	128.5 ± 6.2	2.9 ± 0.3	11.1 ± 4.8	59.3	307.9	277.1

a Gel content.

b Temperature of glass transition determined by DMA analysis as the maximum of the tan δ peak.

c Cross-link density.

d Ultimate flexural strength.

e Flexural modulus.

f Flexural strain at break.

g Temperature of glass transition determined by DSC as half-height midpoint from the second heating scan.

h Temperatures at 5% mass loss in the nitrogen atmosphere.

i Temperatures at 5% mass loss in the air atmosphere.

**Figure 7:**
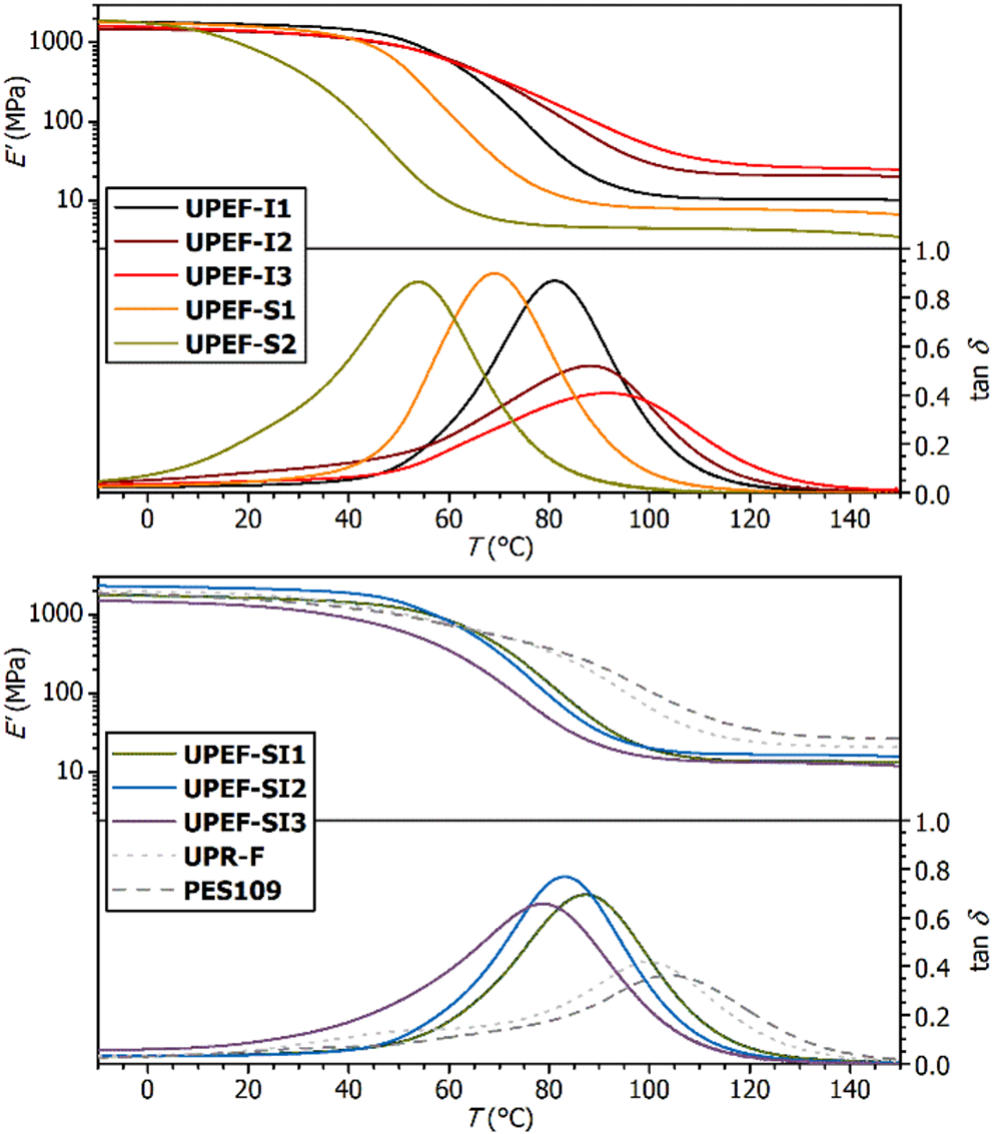
Temperature dependence of storage modulus (*E*′) loss factor (tan δ) for the cured formulations of **UPEF-I** and **UPEF-S** (top), and the **UPEF-SI** series and reference **UPR-F** formulation (bottom).

The thermoanalytical results are represented by the example data obtained for the parent formulation **UPEF-I1**; see Figures 8 and 9. When given enough time to relax their structure, the present materials exhibit a dominant relaxation peak above the half-height midpoint *T*_g,DSC_, which indicates a uniform polymeric structure with a moderately narrow distribution of the relaxation times (corresponding to only a limited number of types of relaxation domains and their movements) and/or a high degree of cooperativity between the relaxing amorphous domains. On erasure of thermal history (by heating the material above *T*_g,DSC_), the relaxation peak completely disappears (Figure 8), which indeed confirms the dominant effect of the cooperativity-based relaxation, i.e., a high degree of cross-linking within the polymer matrix.

**Figure 8:**
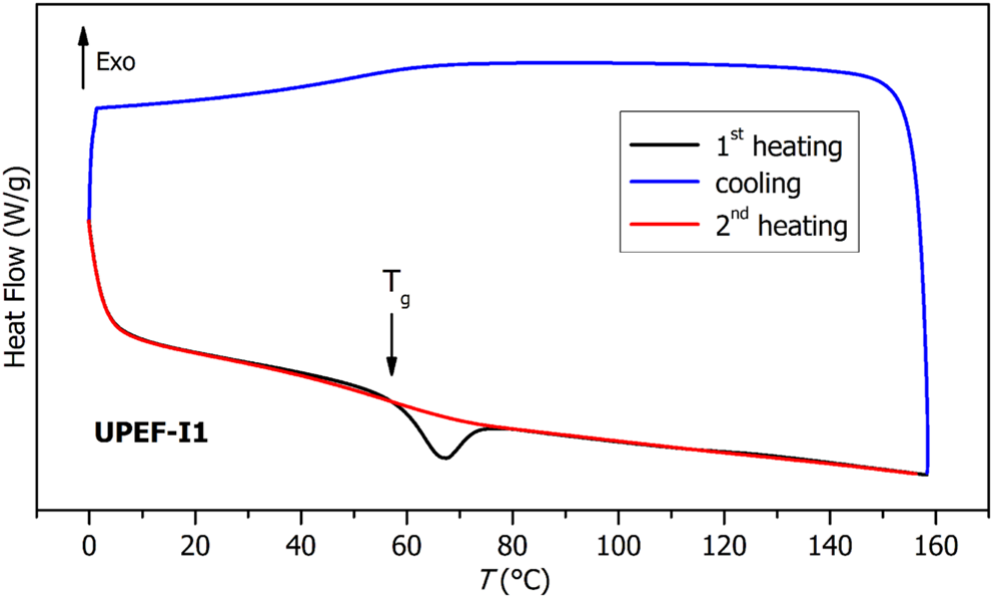
DSC curve for cured **UPEF-I1**.

**Figure 9:**
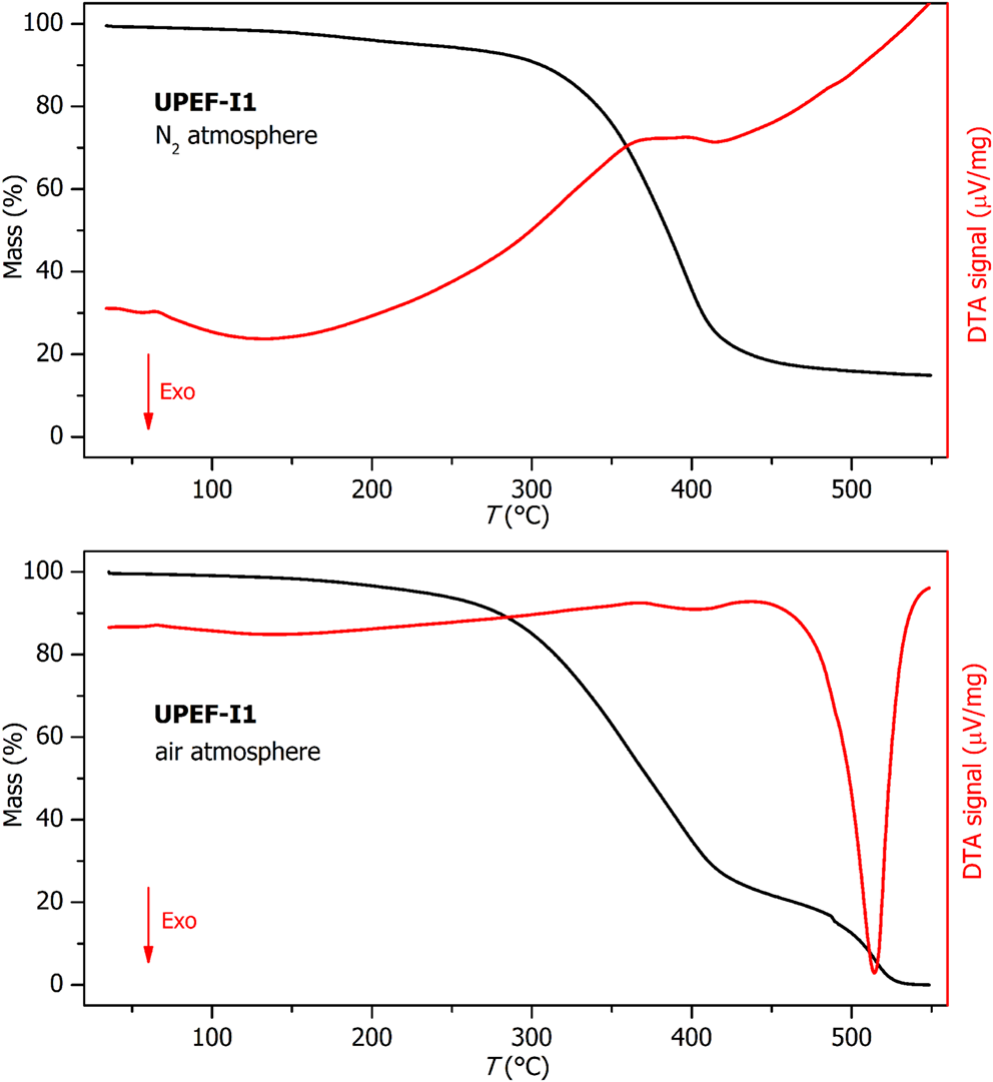
TGA data for **UPEF-I1** measured under N_2_ atmosphere (top) and under air atmosphere (bottom).

Qualitatively, a similar behavior was exhibited by the majority of the measured **UPEF** samples (Figures S40–S46 in the Supporting Information). The only exception was the **UPEF-S** series, where the magnitude of the relaxation peak observed during the first heating scan dramatically decreased with the decline in the IA content (a large portion of IA was replaced by SA in this series). This, again, corresponds well to the loss of interconnectivity (leading to a lower cooperativity among the relaxing domains) within the polymeric matrix being caused by the decreased amount of unsaturated bonds polyester backbone. Note that the **UPEF-S** materials also exhibited the lowest *T*_g,DSC_ values, which will be discussed later. Reference formulations **UPR-F** and **PES109** show *T*_g,DSC_ values of 49.1 and 59.3 °C, respectively, and generally much weaker relaxation behavior than the **UPEF** series (Figures S47 and S48 in the Supporting Information). Interestingly, the main **UPR-F** *T*_g_ value obtained from DSC is significantly lower than the temperature of the tan δ maximum obtained from DMA (*T*_g,DMA_), and corresponds rather to the low-*T* shoulder observed on the tan δ-*T* dependence (Figure 7). Note that the DSC record of the **UPR-F** sample exhibits a second but very weak *T*_g_-like heat capacity change near 90 °C (Figure S47 in the Supporting Information), which would correspond to the temperature of the tan δ maximum.

This DSC/DMA discrepancy might indicate that it is the structural relaxation movements of the segregated low-polar polystyrene domains, which are associated with the largest changes of the bonding arrangements and thus exhibit the dominant enthalpy relaxation signal. The highly cross-linked polyester matrix then, naturally, dictates the mechanical properties, and thus its *T*_g_ (the high-temperature one) dominates the damping factor signal.

Contrary to the glass transition behavior, the thermal decomposition of the **UPEF** materials proceeded very similarly for all studied samples; the example is for the **UPEF-I1** data shown in Figure 9. Under the N_2_ atmosphere, the main degradation step, corresponding to the ∼80% mass loss, occurs between approximately 230 and 450 °C. The decomposition is associated with an endothermic effect of ∼400 J/g. In the case of the measurement carried out in the air atmosphere, the onset of the decomposition appears to be slightly accelerated, but otherwise very similar kinetics are observed; the solid-state decomposition products are, however, further burned in the 470–530 °C range, leaving no solid residuum. The burning is a strongly exothermic effect with an enthalpy of ∼1800 J/g. The TGA data obtained for the rest of the **UPEF** samples were qualitatively as well as quantitatively very similar (see Table 3 and Figures S49–S62 in the Supporting Information). The *T*_5%,N2_ and *T*_5%,air_ parameters of all **UPEF** formulations exceeded 200 °C, demonstrating their high thermal stability, even though they did not reach the values obtained for commercial **PES109** (Table 3). Among the **UPEF** series, somewhat improved stability was observed for the **UPEF-SI** formulations, as their *T*_5%,N2_ and *T*_5%,air_ parameters were found to be higher than 240 and 230 °C, respectively. Note that the much lower thermal stability of the **UPR-F** reference is ascribed to the higher content of volatile styrene oligomers, which is supported by the lower gel content.

From the thermoanalytical point of view, the main differences between the formulations lie in the position of *T*_g_, with the overall span among the cured formulations being 42–63 °C for the DSC data and 54–92 °C for the DMA data. The obtained *T*_g,DMA_ and *T*_g,DSC_ values are listed in Table 3. To quantify the individual contributions of the **UPEF** components, multilinear regression analysis was employed, based on eq 2:

2 $$P=w_{1}\times x_{\mathrm{FDCA}}+w_{2}\times x_{\mathrm{IA}}+w_{3}\times x_{\mathrm{SA}}$$

where *P* is the modeled property (glass transition temperature) and *w*_n_ stands for a weighting coefficient for the respective component (the content of which is expressed in mole fraction, as given in Table 1).

The values of weighting coefficients were optimized based on the least sum of squares residue (SSR) with the Levenberg–Marquardt algorithm employed. Note that incorporation of the EG and DEG contents did not lead to any improvement in the SSR value, as their role is only to compensate for the deficiency of the hydroxyl groups. For the *T*_g,DMA_ values, the correlation the multilinear regression was performed with the correlation coefficient *r* = 0.971; the *T*_g,DSC_ values were predicted with the *r* = 0.954 correlation. To better express the ratio between the contributions of the individual components, the weighting coefficients were normalized according to eq 3:

3 $$z_{n}=\frac{w_{n}}{\sum_{i=1}^{n}w_{n}}$$

The resulting values of the original as well as normalized weighting coefficients are given in Table 4.

**Table 4:** Weighting Coefficients from Multilinear Analysis

property	*w*_1_ FDCA	*w*_2_ IA	*w*_3_ SA	*z*_1_ FDCA	*z*_2_ IA	*z*_3_ SA
*T*_g,DMA_ ^a^	127.0	215.0	23.8	0.352	0.551	0.097
*T*_g,DSC_ ^b^	87.7	137.4	24.3	0.347	0.588	0.065

a Temperature of glass transition determined by DMA analysis as the maximum of the tan δ peak.

b Temperature of glass transition determined by DSC as half-height midpoint from the second heating scan.

As can be seen in Table 4, IA exhibits a dominant influence on the *T*_g_ values, which is expected due to its cross-linking ability (through unsaturated bonds). The amount of FDCA affects the *T*_g_ values to a lesser extent, as it only increases the robustness of the polymeric chain through the higher mass and lower rotational flexibility of the corresponding structural unit. Interestingly, the SA content has very little influence on the *T*_g_ values; its supposed plasticization effect is probably negated to a certain extent by its large polarity, which can contribute to the rigidity of the surrounding polymeric matrix. Furthermore, the molar mass of **UPEF** (see Table 2), which can partially decrease due to the chain-terminating effect of SA, does not dominantly dictate the *T*_g_ value (the *M_n_*–*T*_g_ correlation is only *r* ≈ 0.53). Hence, the SA content is of only secondary relevance to the *T*_g_ values.

The parent formulation (**UPEF-I1**) exhibits very good flexural properties with ultimate flexural strength (σ_f,max_) comparable to those of the reference fumarate/styrene formulations (**UPR-F**, **PES109**); see Figure 10 and Table 3. The increased IA content in **UPEF-I2** and **UPEF-I3** does not improve the mechanical properties of the formulation, probably owing to ineffective cross-linked structures formed by the reaction of adjacent itaconates in the polyester backbone. Reduction of the IA content allows improvement of the ability of the material to withstand greater deformation (*ε*_f,failure_), as documented on **UPEF-S1**. It is ascribed to the increase in chain mobility as a result of a higher content of flexible SA segments and lower cross-link density. However, this comes at the cost of the ability of the material to withstand flexural stress (σ_f,max_), which is commonly influenced by chain mobility in an inverse manner. Note that a further decrease in the IA content led to deterioration of the mechanical properties owing to the inferior polymeric network mentioned above. The highest value of the ultimate flexural strength (σ_f,max_ = 140.0 ± 6.4 MPa) was observed for the **UPEF-SI2** formulation, which reflects the optimal SA/FDCA ratio. Note that a further increase in the SA/FDCA ratio in **UPEF-SI3** leads to the expected decrease in the value of σ_f,max_. In summary, the mechanical properties of the formulations presented here, except **UPEF-SI2**, meet the basic requirements for material application, since their flexural parameters fit the ranges of commercially relevant UP resins (σ_f,max_ = 80–160 MPa, *ε*_f,failure_ = 2–7%).^44^

**Figure 10:**
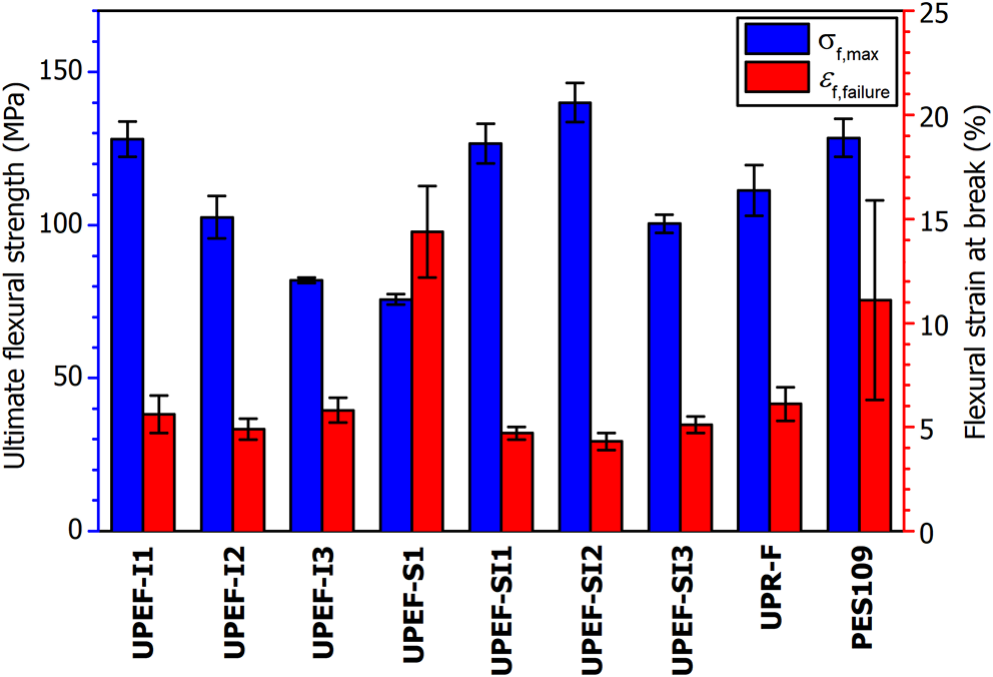
Flexural properties of cured formulations of the **UPEF** series and references (**UPR-F** and **PES109**). Ultimate flexural strength (blue) and flexural strain at break (red).

## Conclusions

This study has shown a promising pathway for the chemical upcycling of postconsumer PEF waste, which will be generated after the planned replacement of PET in food and beverage packaging. Because PEF is not a commonly available polymer at this stage, we synthesized it in our laboratory and then depolymerized it by glycolysis using readily available DEG. The mixture of hydroxy-terminated oligomers produced was successfully used for the synthesis of a series of unsaturated polyester resins. For this purpose, biobased itaconic acid was chosen as a source of unsaturation, as it allows the formulation of styrene-free systems due to the copolymerization parameters being more convenient than in the case of commonly used fumarate polyesters.^47^

To demonstrate the application potential of the synthesized polyesters, the formulations in DMI were subjected to mechanical and thermoanalytical testing. Note that DMI was used as a reactive diluent for its low volatility, low flammability, and nontoxicity. Furthermore, it is accessible from renewable resources.^51^ Our study has shown that the properties of cured formulations are strongly affected by a variation of the IA content in the polyester backbone, which regulates the density of the polymer network. Note that the IA content should not fall below 20 molar %, as a lower content of double bonds in the polyester backbone does produce a sparsely cross-linked network. It results in a decreased gel content, a drop of *T*_g_ value, and deterioration of the mechanical properties.

The variation in polyester composition shows that the concept described here can produce formulations with flexural strength comparable to that of conventional fumarate-based polyesters formulated in styrene. This opens an avenue to the sustainable valorization of waste formed from biobased polyesters. Unlike current industrial technologies, the production of itaconate-based polyesters is hindered by the need to prevent isomerization, making the process much more time-consuming. On the other hand, the procedures described here are easily scalable without special equipment, as they do not require any additional purification compared to fumarate-based technologies. Moreover, PEF waste does not need any extensive pretreatment beyond sorting from other polymer blends, and the technology does not generate any waste or side products, which fits the principles of green chemistry.

## Experimental Section

### Materials

Dimethyl itaconate (DMI), furan-2,5-dicarboxylic acid (FDCA), and succinic acid (SA) were supplied from BLDPharmatech. Itaconic acid (IA), maleic anhydride, propylene glycol (propane-1,2-diol), hydroquinone, toluene-4-sulfonic acid monohydrate (PTSA), and hexafluoropropan-2-ol (HFIP) were supplied from Thermo Fisher Scientific. Diethylene glycol (DEG), ethylene glycol (EG), phthalic anhydride, sulfuric acid (96%), sodium sulfate (anhydrous), and tetrahydrofuran (THF) were supplied from Penta. Dichloromethane, ethyl acetate, methanol, and toluene were supplied by Lach-Ner. Dibutyltin­(IV) oxide (DBTO), and tin­(II) 2-ethylhexanoate were obtained from the PMC Group. Polyester resin ChS-Polyester 109 (**PES109**; 60 wt % in styrene), butanone peroxide (32% in a mixture of dimethyl phthalate and dicetone alcohol; MEKP), and cobalt carboxylate (in organic solvents, 4 wt % of Co) were obtained from Stachema. Oxidovanadium­(IV) dibutylphosphate (10.6% V, VOP) was synthesized as described elsewhere.^48^

### Synthesis of Dimethyl Furan-2,5-dicarboxylate (DMFDC)

A suspension of FDCA (100 g, 0.64 mol) in methanol (350 mL) was treated with sulfuric acid (15 mL) and heated at reflux for 1 h. After that, the hot reaction mixture was treated with sulfuric acid (35 mL) dropwise and heated under reflux for 5 h. After the mixture was cooled to room temperature, white crystals precipitated from the reaction mixture. The precipitate was filtered off on a glass frit and rinsed with a saturated solution of sodium carbonate until the filtrate became pH neutral. Crude product was dissolved in ethyl acetate (∼150 mL), washed with brine (2 × 100 mL), and dried with anhydrous sodium sulfate. Volatiles were vacuum evaporated to give the final product. Yield was 89.6 g (0.49 mol, 76%). Colorless crystals. Anal. Calc. (C_8_H_8_O_5_): C, 52.18; H, 4.38. Found: C, 52.46; H, 4.59. ^1^H NMR (CDCl_3_, 400 MHz), δ (ppm): 7.20 (s, 2H, furan), 3.91 (s, 6H, Me).

### Synthesis of Oligomeric PEF

A mixture of DMFDC (80 g, 0.43 mol) with EG (135 g, 2.17 mol) was treated with tin­(II) 2-ethylhexanoate (0.4 g). The reaction mixture was heated to 230 °C and the formed methanol was slowly distilled off during 2.5 h. In the next step, residual methanol and excess EG were vacuum evaporated. The pressure in the setup was very slowly decreased (during 2h) to 400 Pa, while the temperature of the reaction mixture was heated to 230 °C. *Note*: The methyl ester residues were detected by ^1^H NMR spectroscopy when distillation was done faster or at lower temperatures. When 86 mL of EG was distilled off, the process was stopped. While hot, the viscous liquid was transferred to a beaker and cooled to room temperature to give a white solid of oligomeric PEF (108 g).

### Synthesis of Medium-Molar Mass PEF by Melt Polymerization

Melt polymerization was performed in a Kugelrohr distillation apparatus. The oligomeric PEF (75 g) was heated under a nitrogen atmosphere to 180 °C, and the pressure in the apparatus was gradually reduced to 10 Pa, while the EG was distilled off. After 2h, the temperature was increased to 250 °C and melt polymerization continued for 6 h, while the viscosity of the mixture increased and the color changed from yellow to orange. After that, the melted product was removed from the reaction flask, cooled, ground, washed with methanol and tetrachloroethylene, and vacuum-dried to give 52 g of bottle-grade PEF. *Caution: cooling of the PEF melt in a glass flask can cause its destruction due to crystallization of the PEF polymer.*

### Synthesis of High-Molar Mass PEF by Solid-State Polymerization

Solid-state polymerization was performed in a Kugelrohr distillation apparatus at a pressure of 10 Pa. Fine powder of bottle-grade PEF (2.5 g) was heated to 160 °C for 1 h. After that, the temperature gradually increased. The powder was heated to 180 °C for 1 h and to 200 °C for 14 h. After cooling to room temperature, the crude product was dissolved in HFIP (10 mL), diluted with CH_2_Cl_2_ (10 mL), and filtered through 0.45 μm PTFE filters, precipitated with methanol. The final product was washed with methanol and vacuum-dried to give 2 g of white powder.

### Synthesis of GLF

PEF powder (50 g) was treated with DEG (29.2 g, 0.27 mol) under a nitrogen atmosphere and heated to 240 °C and vigorously stirred by an anchor stirrer (300 rpm) for 4 h. The resulting viscous liquid was cooled to room temperature and used without further purification.

### General Procedure for Preparation of Unsaturated Polyester (UP) Formulations

A mixture of monomers (composition is given in Table 5), DBTO (29 mg), PTSA (70 mg), and hydroquinone (20 mg) was heated under a nitrogen inlet while stirred with an anchor stirrer (300 rpm). At 160 °C, the melt reaction mixture was treated with toluene (∼10 mL), and the nitrogen flow was stopped. The temperature of the reaction mixture was regulated by the amount of toluene to not exceed 165 °C and produced water was separated using a Dean–Stark apparatus. When the acid value dropped below 50 mg/g KOH, the temperature of the mixture was kept at 160 °C and volatiles were vacuum evaporated. After cooling to 80 °C, a sample for analysis was collected, and the produced polyester was diluted by DMI (15.2 g) to give a formulation of 60 wt % UP content. We note that DMI was melted in a water bath before use.

**Table 5:** Monomeric Composition of the UPs

polyester	**GLF** (g)	IA (g)	SA (g)	DEG (g)
**UPEF-I1**	20.7	9.3	0.0	0.0
**UPEF-I2**	16.1	10.9	0.0	3.0
**UPEF-I3**	11.8	12.4	0.0	5.8
**UPEF-S1**	16.3	7.4	3.3	3.0
**UPEF-S2**	12.0	5.4	6.6	5.9
**UPEF-SI1**	18.4	9.2	0.8	1.5
**UPEF-SI2**	16.2	9.1	1.7	3.0
**UPEF-SI3**	11.9	9.0	3.3	5.9

### Preparation of UPR-F Formulation

A mixture of monomers phthalic anhydride (74 g, 0.5 mol), maleic anhydride (49 g, 0.5 mol), propylene glycol (38 g, 0.5 mol), EG (31 g, 0.5 mol), DBTO (0.19 g), PTSA (0.48 g), and hydroquinone (96 mg) was heated under a nitrogen inlet while stirring with an anchor stirrer (300 rpm). At 240 °C, the melt reaction mixture was treated with toluene (∼20 mL), and the nitrogen flow was stopped. The temperature of the reaction mixture was regulated by the amount of toluene to not exceed 240 °C and the produced water was separated using a Dean–Stark apparatus. When the acid value dropped below 30 mg/g KOH, the temperature of the mixture was kept at 160 °C and volatiles were vacuum evaporated. After cooling to 80 °C, the produced polyester was diluted by styrene (111 g, 1.1 mol) to give a formulation of 60 wt % UP content. The mixture was stirred under heating to 150 °C until it became homogeneous. The final product was stored at room temperature.

### Curing of UP Formulations and Preparation of Test Specimens

Given **UPEF** formulation (25 g) was treated with VOP (100 mg) and thoroughly stirred. The mixture was treated with MEKP (400 μL) and vigorously stirred to obtain a homogeneous blend. After degassing in a centrifuge (6000 rpm for 2 min), the resin was then poured into silicone molds and cured at room temperature overnight. The next day, the specimens were postcured in an oven under the following conditions: at 50 °C for 1 h, 70 °C for 1 h, 90 °C for 1 h. Finally, all samples were cooled down slowly to ambient temperature to avoid thermal stress. Specimen shape: rectangular cuboid of dimensions 50 × 6 × 3 mm^3^. The **UPR-F** specimens were prepared in a similar way, but the formulation (25 g) was treated with a lower amount of the redox initiation system: VOP (25 mg) and MEKP (100 μL). The initiation of the **PES109** resin (25 g) was performed according to the supplier’s recommendation using cobalt carboxylate (62.5 mg) and MEKP (210 μL).

### Gel Content

The specimens were cryogenically ground in an IKA A10 Basic impact mill cooled with liquid nitrogen. The ground material (∼1 g) was precisely weighed (*m*_1_), treated with THF (30 mL), and stirred in a closed Erlenmeyer flask overnight. Solids were filtered off, dried in an oven at 50 °C overnight, and weighed (*m*_2_). The gel content (in %) was calculated according to eq 4:

4 $$\mathrm{GEL}=\left(m_{2}/m_{1}\right)\times 100$$

### Intrinsic Viscosity

Samples of PEF were dissolved in a 3/2 mixture (w/w) of phenol/1,1,2,2-tetrachloroethane (*c* = 5 mg/mL) and measurements were done at 30 °C using an Ubbelohde viscometer.^52^ Intrinsic viscosity [η] was calculated according to eq 5:

5 $$\left[\eta \right]={\left[2\left\{t/t_{0}-\ln\left(t/t_{0}\right)-1\right\}\right]}^{0.5}/c$$

where *c* is the concentration of the solution. Variables *t*_0_ and *t* are the flow times of pure solvent and solution of polymer, respectively. Number-average molar mass was calculated according to the Mark–Houwink equation^35^ using the Berkowitz fit for PET (eq 6):^53^

6 $$M_{n,\mathrm{Berkowitz}}=3290\times {\left[\eta \right]}^{1.54}$$

### NMR Spectroscopy

The ^1^H NMR and ^1^H–^1^H COSY NMR spectra of the samples were collected on Bruker Avance 400 and Bruker Avance 500 spectrometers at room temperature. Chemical shifts are reported in parts per million relative to tetramethylsilane.

### Vibrational Spectra

Infrared spectra were collected on a Nicolet iS50 FTIR spectrometer (Thermo Fisher Scientific) in the region of 4000–400 cm^–1^ (data spacing = 0.5 cm^–1^) using a built-in diamond ATR crystal. Raman spectra were collected on an FT-Raman module (Nd:YAG excitation laser, λ = 1064 nm, power = 0.5 W, data spacing = 0.5 cm^–1^) of the Nicolet iS50 spectrometer in the region of 4000–400 cm^–1^.

### Chromatography

Size exclusion chromatography (SEC) was performed on a Waters liquid chromatograph Alliance e2695 with a refractive index (RI) detector 2414 and two Agilent Mixed-E columns 300 × 7.5 mm (**GLF**) or two Agilent Mixed-C columns 300 × 7.5 mm (**UPEF** series and **UPR-F**). THF was used as the mobile phase at a flow rate of 1 mL/min. The samples were dissolved in THF at concentration ≈3 mg/mL and filtered through 0.45 μm filters (injected volume = 100 μL). The calibration on polystyrene standards covered the molar mass ranges of 162 to 30,000 g/mol (Mixed-E columns) and 162 to 6 × 10^6^ g/mol (Mixed-C columns).

An experimental setup for SEC with a multiangle light scattering (MALS) detector consisted of an Agilent 1200 Series isocratic pump and an autosampler coupled with a MALS detector HELEOS and an RI detector Optilab T-rEX. ASTRA 8 software was used for data collection and processing. The software and detectors were purchased from Waters | Wyatt Technology. The separation was performed with two Agilent PLgel Mixed-C 300 × 7.5 mm columns and THF at a flow rate of 1 mL/min. The samples were prepared in THF at a concentration of ≈7 mg/mL, filtered with 0.45 μm filters, and injected in a volume of 100 μL.

### Viscosity

It was measured on a Höppler’s Rheo-Viscometer (Veb Prufgerate-Werk) with cells 0.1 (*k* = 0.11302), 1 (*k* = 1.05758), 10 (*k* = 10.64967), and 40–360 g weights at a temperature of 25 °C.

### Green Chemistry Metrics

Atom economy (AE) and the E-factor were calculated according to eqs 7 and 8:

7 $$\mathrm{AE}=\sum \left(y\times M_{\mathrm{product}}\right)/\sum \left(x\times M_{\mathrm{reactant}}\right)\times 100$$

8 $$\mathrm{E‐factor}=m_{\mathrm{waste}}/m_{\mathrm{product}}$$

where *M*_product_ is the molar weight of a product (idealized glycolysis product; polyester segment), *M*_reactant_ is molar weight of a reactant, *x* and *y* are stoichiometric coefficients, *m*_waste_ is mass of waste, and *m*_product_ is mass of product.^49^

### Dynamic Mechanical Analysis

The analysis was performed on a DMA303 Eplexor device (Netzsch) in a single fixed-point cantilever configuration with a span between clamps of 11 mm, a deviation of ± 0.15 mm, and a frequency of 1 Hz. Measurements were made in the range −60 to 200 °C and a heating rate of 3 °C/min.

### Mechanical Tests

Flexural testing was carried out on specimen A using a universal testing machine Autograph AGS-X 50kN (Shimadzu) in a three-point bending configuration with a support span of 40 mm at a crosshead speed of 1 mm/min. The experiment was stopped when the specimen ruptured or the force exerted on the specimen was less than 10 N.

### Thermoanalytical Measurements

The differential scanning calorimetry (DSC) was used to perform heating scans (at 10 °C/min in the 0–160 °C temperature range) of the PEF and **UPEF** samples under a static air atmosphere. In this regard, the heat-flow DSC Q2000 (TA Instruments) was used, equipped with an autosampler, RCS90 cooling accessory, and T-zero technology. The DSC was calibrated using the In, Zn, and Ga metal standards. The samples were measured in the form of compact pieces with masses ∼10 mg. Identical samples were also used for the thermogravimetric (TGA) measurements, which were performed using the STA 449 F5 Jupiter instrument (Netzsch) equipped with a DSC/TG holder. The TGA runs were also realized as heating scans at 10 °C/min; the investigated temperature range was 30–550 °C. Two series of the TGA measurements were performed, either in the N_2_ or in the air atmosphere; the flow of the purge gas was set to 50 mL/min.

## Supplementary Material



## Notes

Infrared and Raman spectra, NMR spectra, SEC chromatograms, DSC data, TGA data, stress–strain curves, green chemistry metrics (PDF)

## Abbreviations

AE: atom economy
AV: acid value
DBTO: dibutyltin­(IV) oxide
DEG: diethylene glycol
DMFDC: dimethyl furan-2,5-dicarboxylate
DMI: dimethyl itaconate
DSC: differential scanning calorimetry
E-factor: environmental factor
EG: ethylene glycol
FDCA: furan-2,5-dicarboxylic acid
GHG: greenhouse gas
HFIP: hexafluoropropan-2-ol
IA: itaconic acid
MALS: multiangle light scattering
MEKP: butanone peroxide
MES: mesaconate
OA: Ordelt adducts
PEF: poly­(ethylene-2,5-furanoate)
PET: poly­(ethylene terephthalate)
PTSA: toluene-4-sulfonic acid monohydrate
RI: refractive index
SA: succinic acid
SEC: size-excluded chromatography
SSR: sum of squared residue
TGA: thermogravimetry
THF: tetrahydrofuran
UP: unsaturated polyester
VOP: oxidovanadium­(IV) dibutylphosphate
