# Supplementary material for: Depolymerized Poly(ethylene-2,5-furanoate) as a Sustainable Feedstock for Biobased Unsaturated Polyester Resins
Source: Macromolecules. 2025 Sep 25;58(19):10324–35. doi: 10.1021/acs.macromol.5c01600 (PMC12530047; doi:10.1021/acs.macromol.5c01600)
Supplement: Supplementary file 1 [file ma5c01600_si_001.pdf]

Supporting Information for:

Depolymerized Poly(ethylene-2,5-furanoate) as a Sustainable Feedstock for Bio-based Unsaturated Polyester Resins.

Tomáš Foltýn,[†] Jan Všetečka,[†] Roman Svoboda,[‡] Štěpán Podzimek,[†,⊥] Jaromír Vinklárek[‖] and Jan Honzíček[†,]*

[†]Institute of Chemistry and Technology of Macromolecular Materials, Faculty of Chemical Technology, University of Pardubice, Studentská 573, 532 10 Pardubice, Czech Republic

[‡] Department of Physical Chemistry, Faculty of Chemical Technology, University of Pardubice, Studentská 573, 532 10, Pardubice, Czech Republic

[⊥] Synpo Ltd, S. K. Neumanna 1316, 532 07 Pardubice, Czech Republic

[‖] Department of General and Inorganic Chemistry, Faculty of Chemical Technology, University of Pardubice, Studentská 573, 532 10 Pardubice, Czech Republic

Table of Contents

Characterization of PEF by ^{1}H NMR spectroscopy	S3
Characterization of **GLF** by NMR spectroscopy and SEC chromatography	S4
Characterization of **UPEF** polyesters by infrared and Raman spectroscopy	S7
Characterization of **UPEF** polyesters by ^{1}H NMR spectroscopy	S13
Characterization of **UPEF** polyesters by SEC-MALS	S17
Characterization of cured **UPEF** resins by DSC	S22
Characterization of cured **UPEF** resins by TGA	S27
Stress-strain curves of cured **UPEF** resins	S36
Composition of synthesized **UPEF** polyesters	S36
Gren chemistry metrics for **UPEF** polyesters	S37

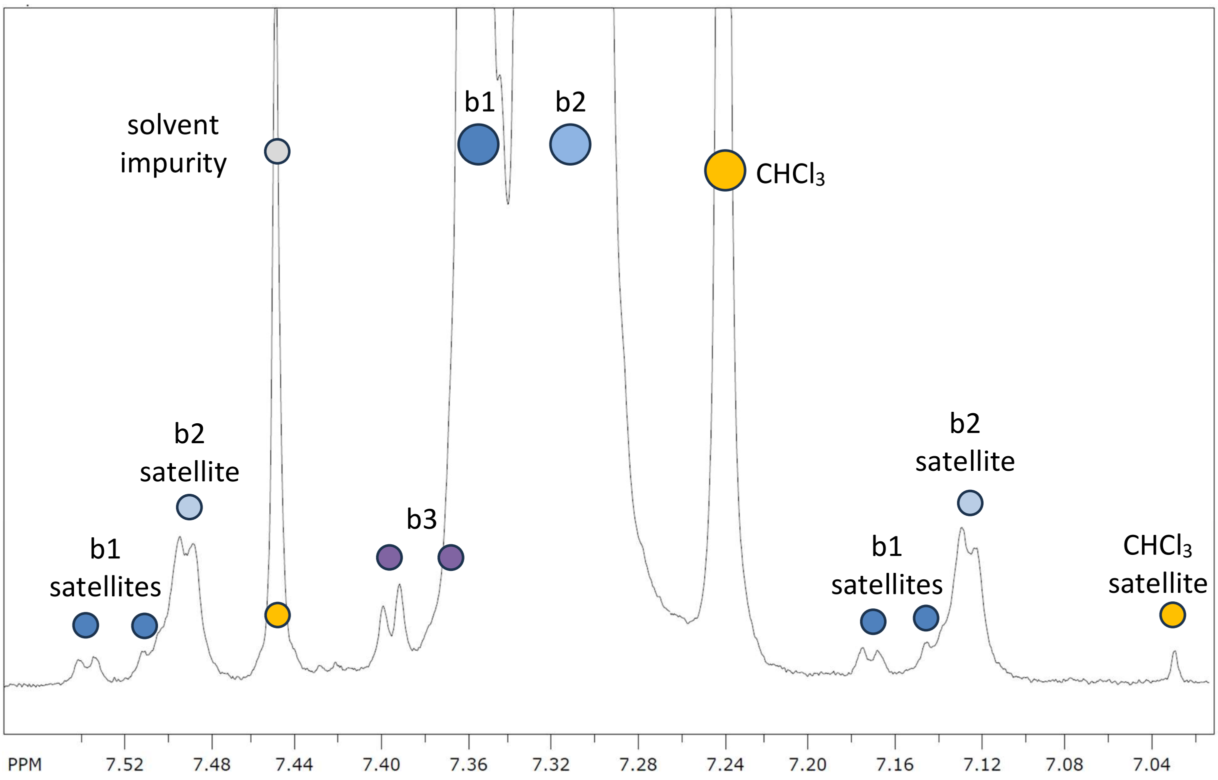

Figure S1. Aromatic part of ^{1}H NMR spectrum of low molar-weight PEF (M_n = 1 600 g/mol). Measured in mixture $CDCl_3/CF_3COOD$.

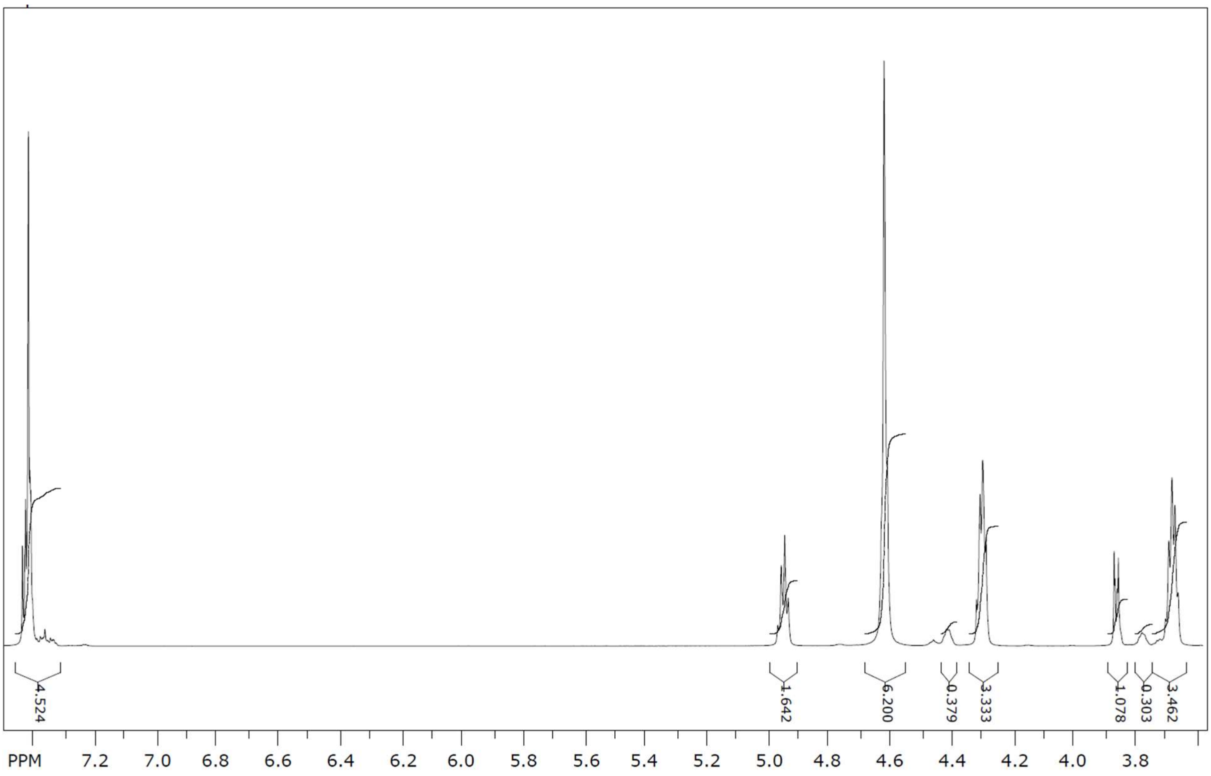

Figure S2. ^{1}H NMR spectrum of oligomeric PEF (M_n ~ 500 g/mol) measured in DMSO-d^6.

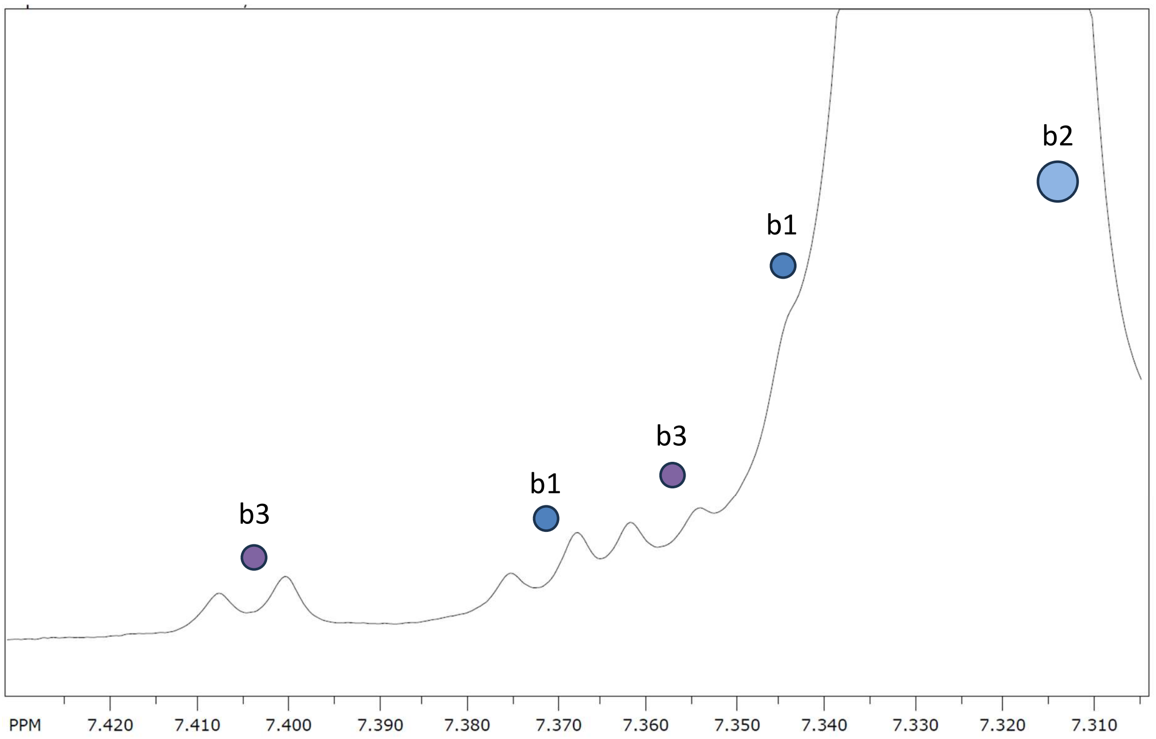

Figure S3. Aromatic part of ^{1}H NMR spectrum of medium molar-weight PEF (M_n = 23 000 g/mol). Measured in mixture $CDCl_3$/CF_3COOD.

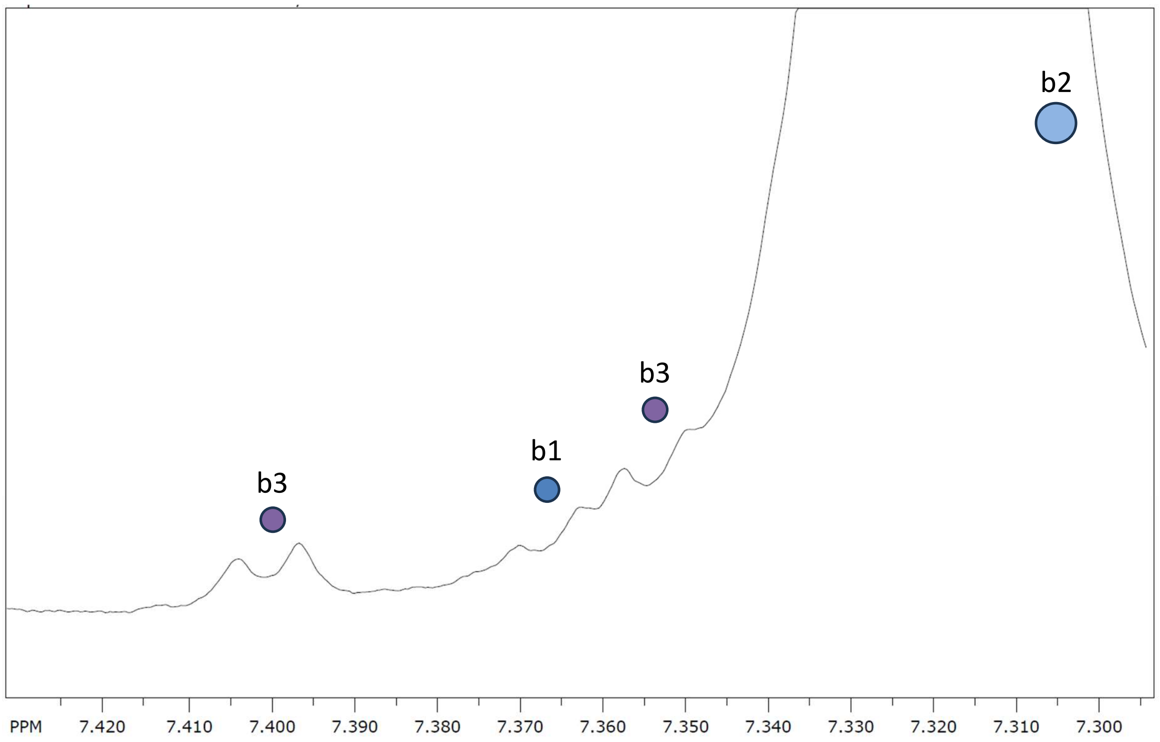

Figure S4. Aromatic part of ^{1}H NMR spectrum of high molar-weight PEF (M_n = 50 000 g/mol). Measured in mixture $CDCl_3$/CF_3COOD.

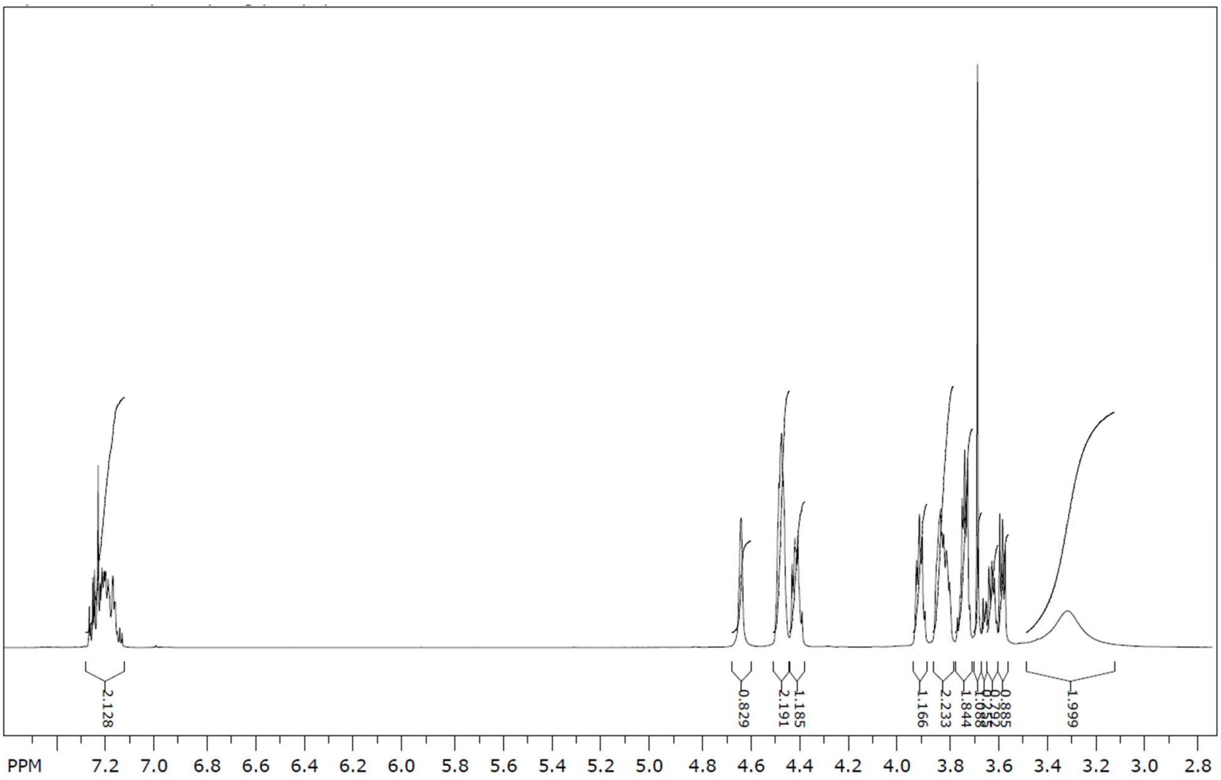

Figure S5. ^{1}H NMR spectrum of **GLF** in $CDCl_3$.

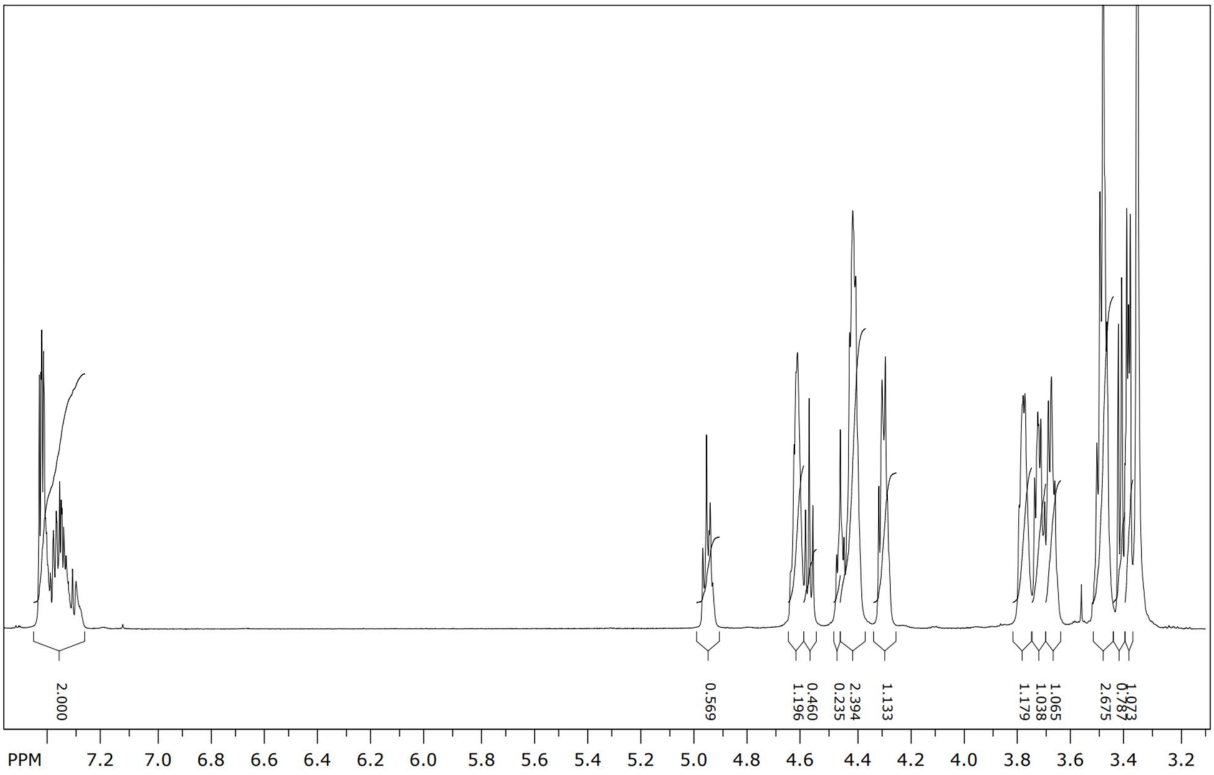

Figure S6. ^{1}H NMR spectrum of **GLF** from high molecular weight PEF in DMSO-d^6.

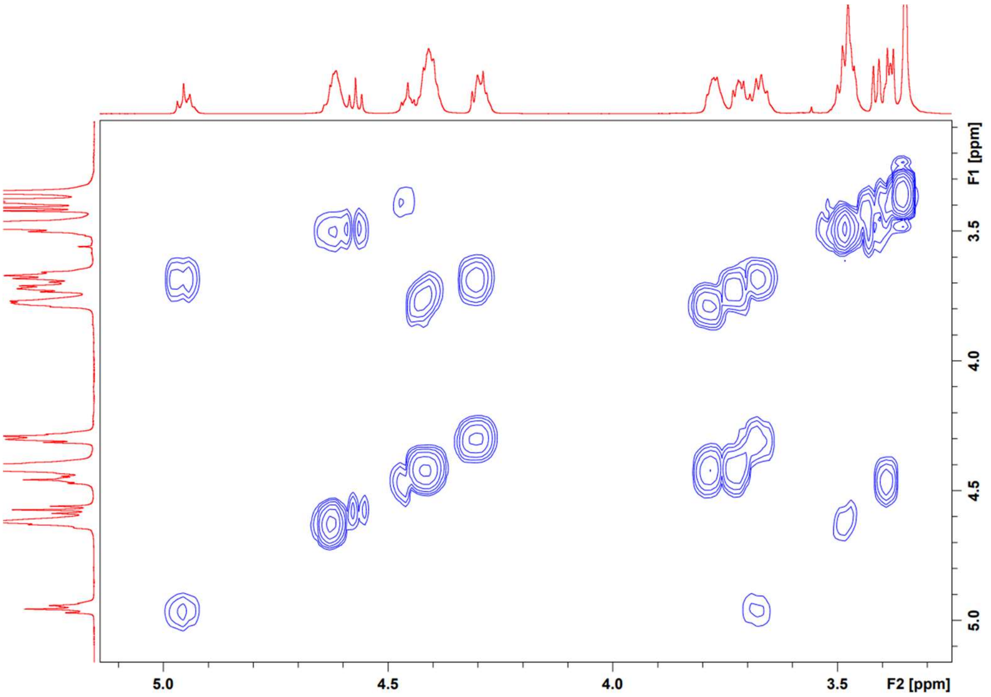

Figure S7. ^{1}H-^{1}H COSY of **GLF** in DMSO-d^6.

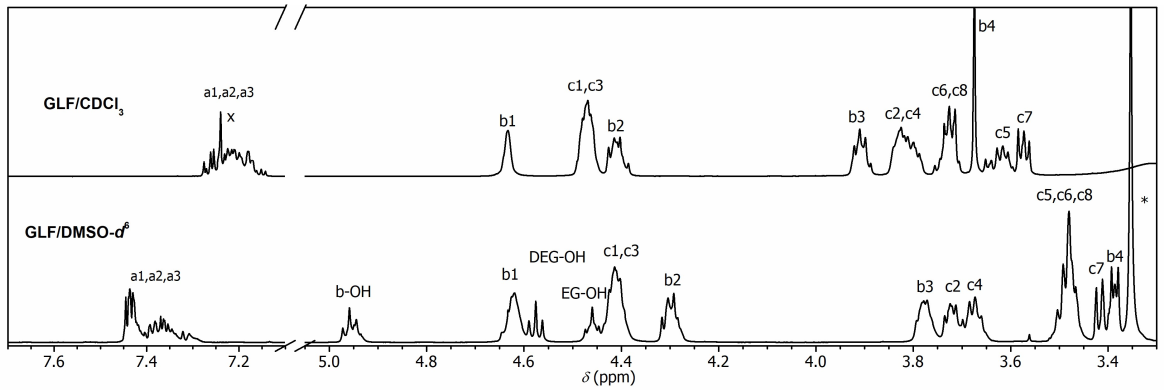

Figure S8. ^{1}H NMR spectra of **GLF** in $CDCl_3$ (top) and DMSO-d^6 with assignment of signals. Notation according to Fig. 1.

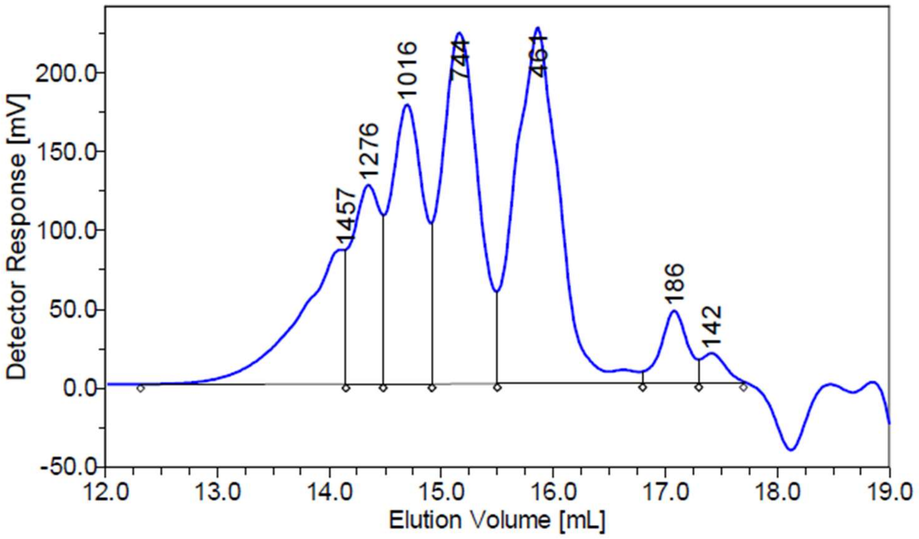

	MP (Daltons)	Area	% Area	Height	Retention Time (min)
1	1457	2503381	12.1	85105	14.150
2	1276	2196818	10.6	126182	14.352
3	1016	3623586	17.5	177257	14.696
4	744	5125003	24.7	222609	15.163
5	461	6219433	30.0	225816	15.861
6	186	767842	3.7	46046	17.082
7	142	283294	1.4	19126	17.414

Figure S9. SEC chromatogram of **GLF** and relative peak areas.

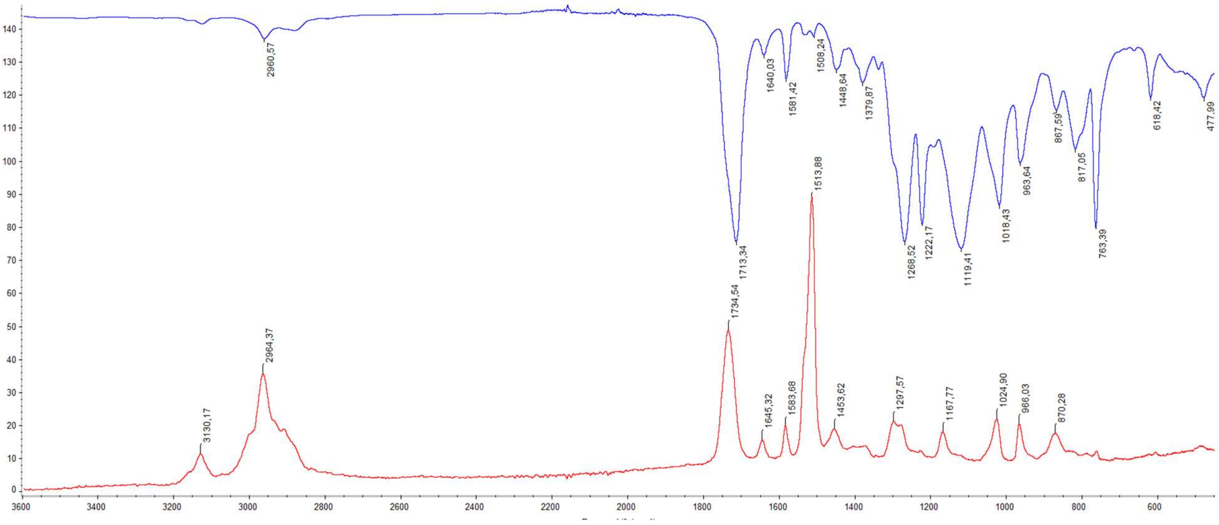

Figure S10. Infrared spectrum (top) and Raman spectrum (bottom) of **UPEF-I1**.

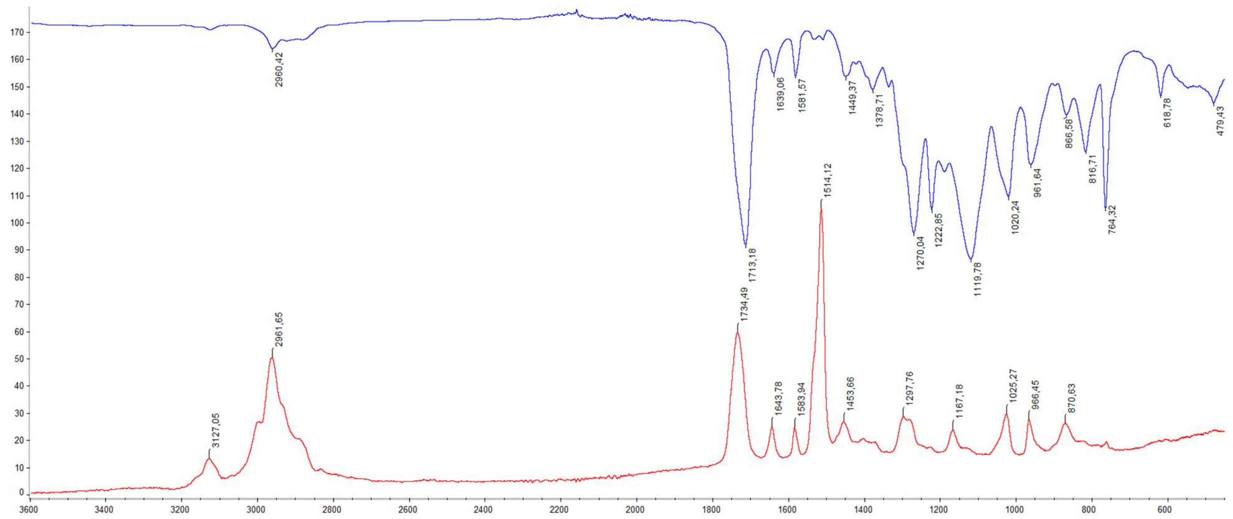

Figure S11. Infrared spectrum (top) and Raman spectrum (bottom) of **UPEF-I2**.

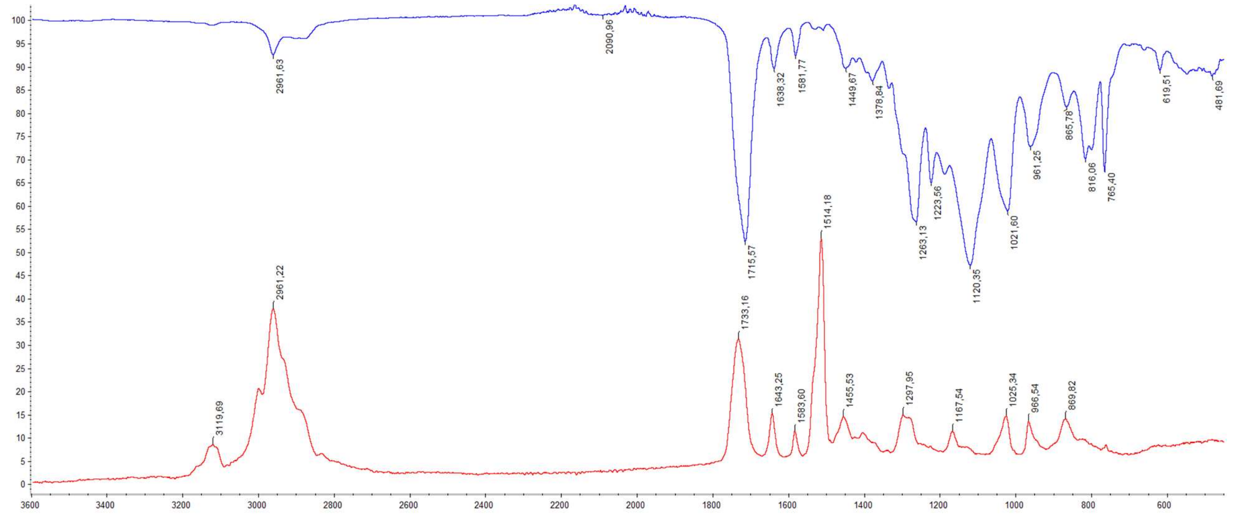

Figure S12. Infrared spectrum (top) and Raman spectrum (bottom) of **UPEF-I3**.

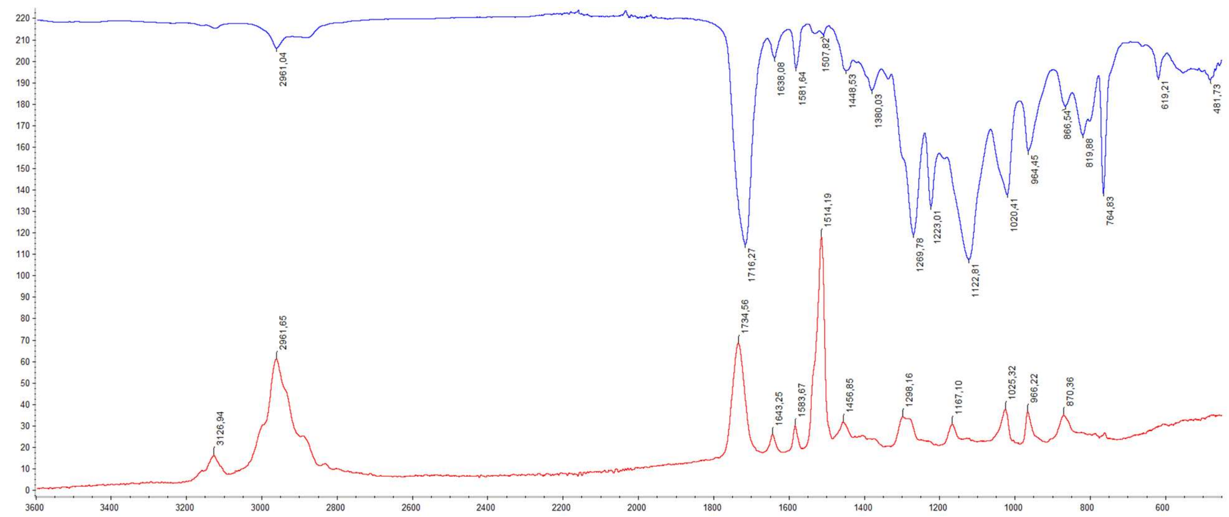

Figure S13. Infrared spectrum (top) and Raman spectrum (bottom) of **UPEF-S1**.

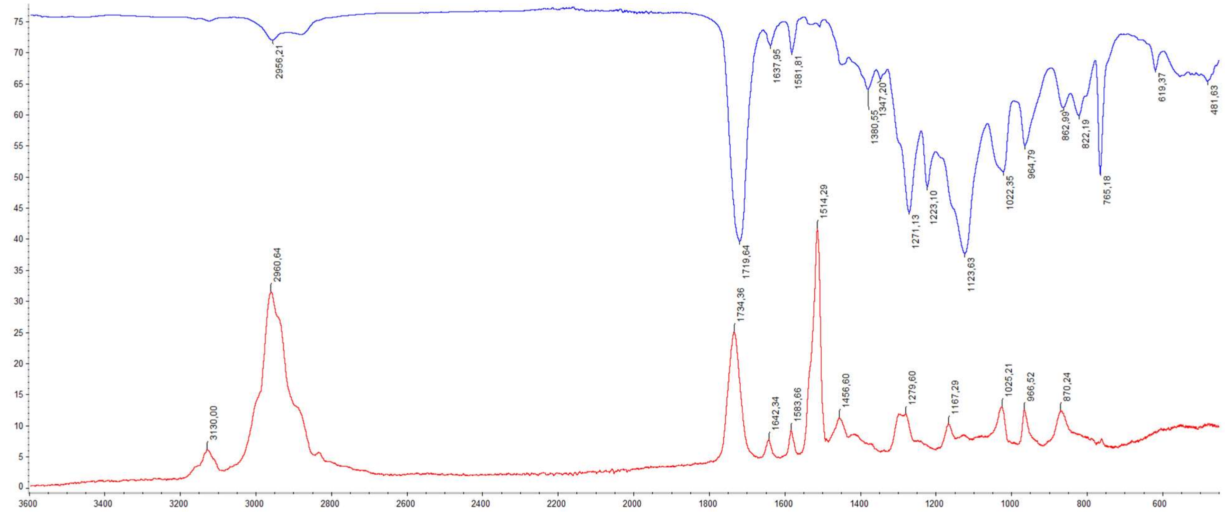

Figure S14. Infrared spectrum (top) and Raman spectrum (bottom) of **UPEF-S2**.

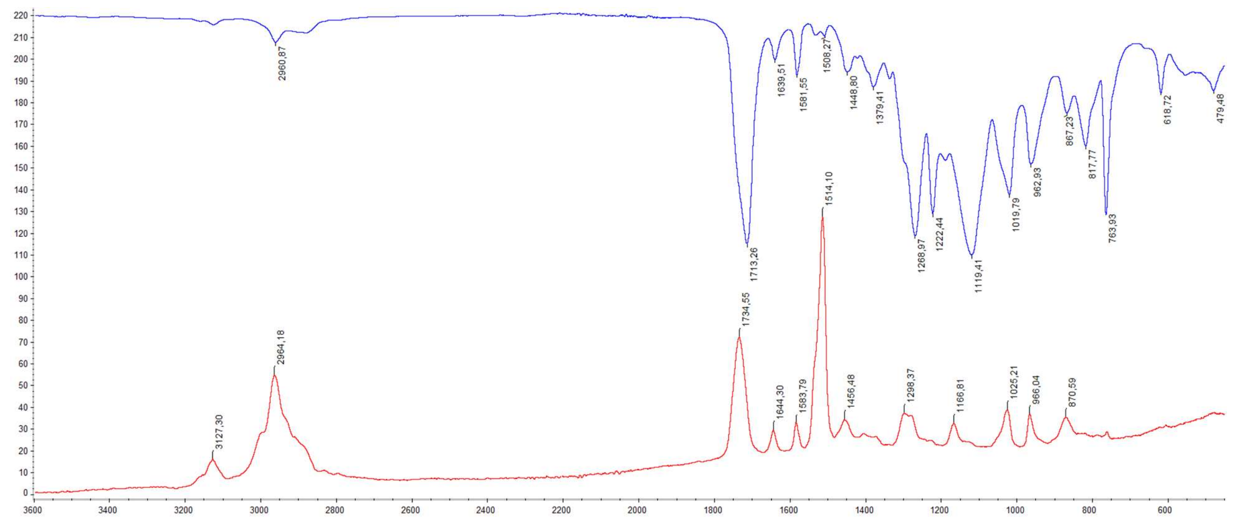

Figure S15. Infrared spectrum (top) and Raman spectrum (bottom) of **UPEF-SI1**.

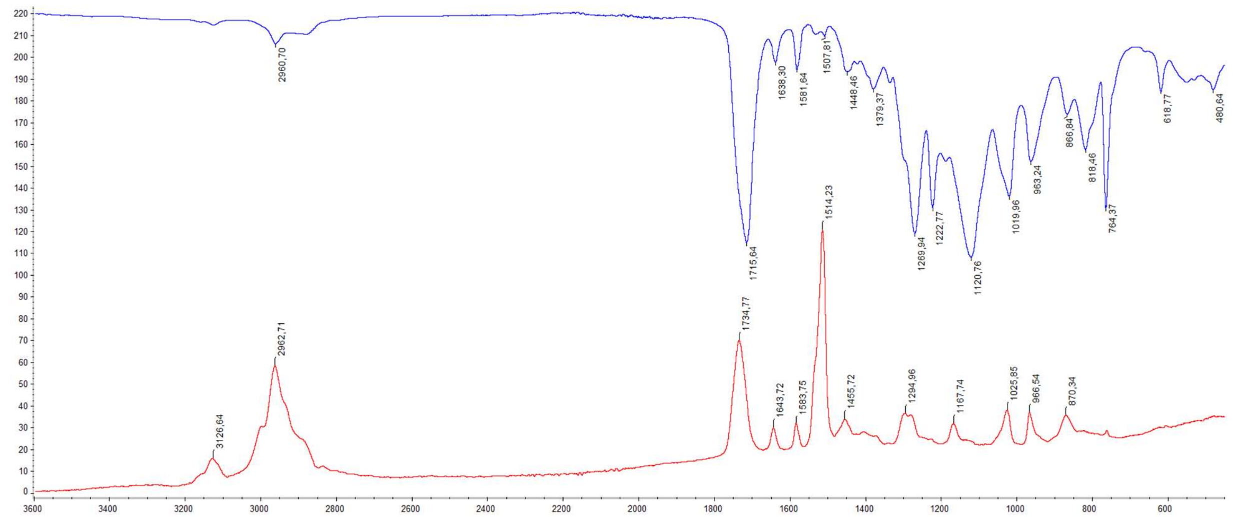

Figure S16. Infrared spectrum (top) and Raman spectrum (bottom) of **UPEF-SI2**.

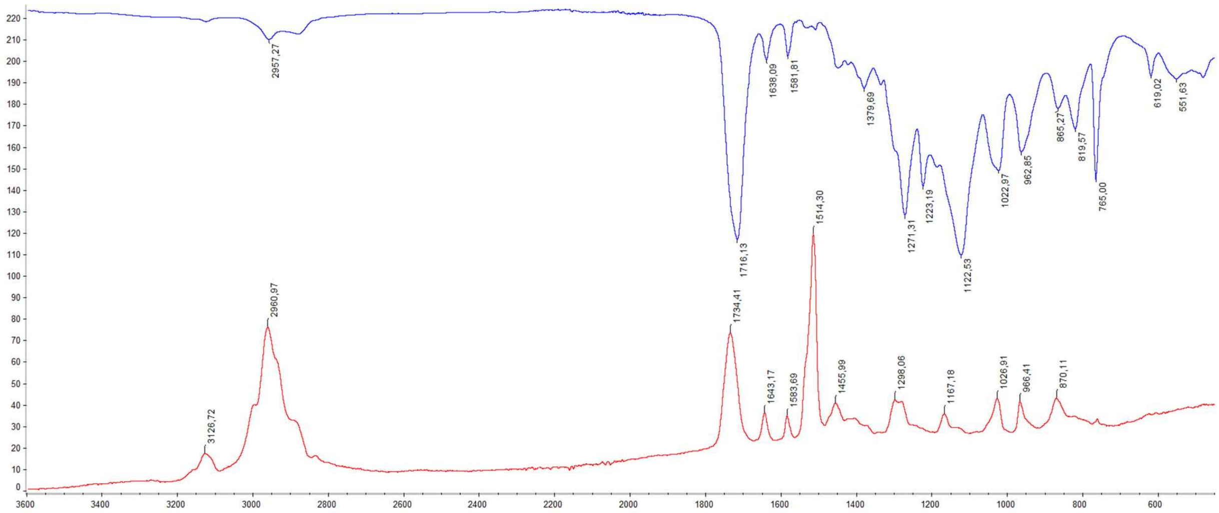

Figure S17. Infrared spectrum (top) and Raman spectrum (bottom) of **UPEF-SI3**.

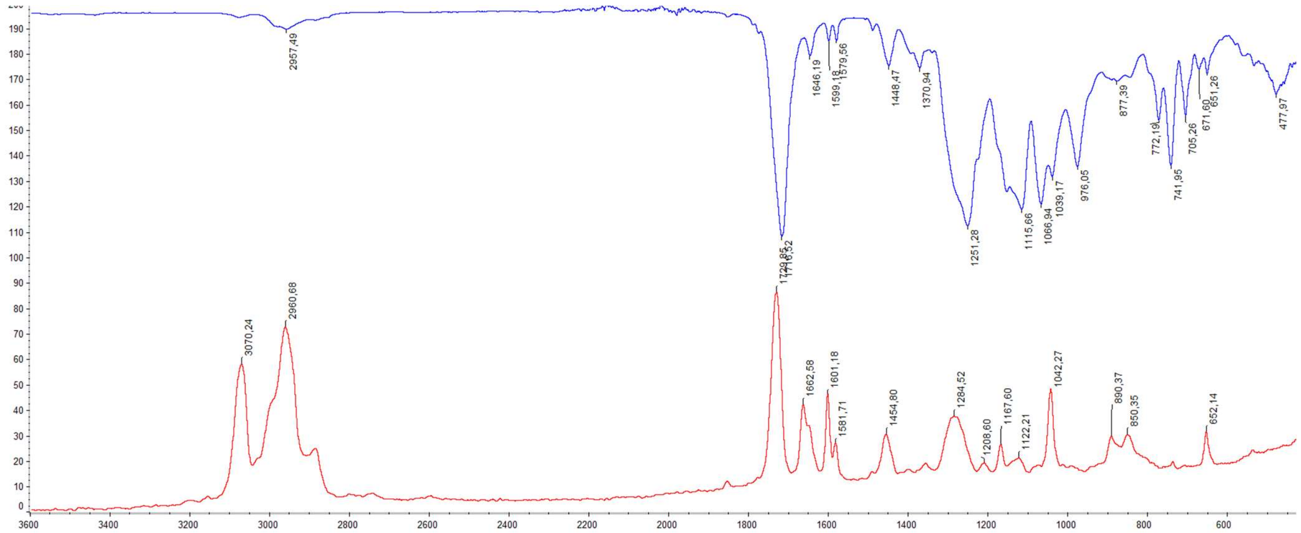

Figure S18. Infrared spectrum (top) and Raman spectrum (bottom) of **UPR-F**.

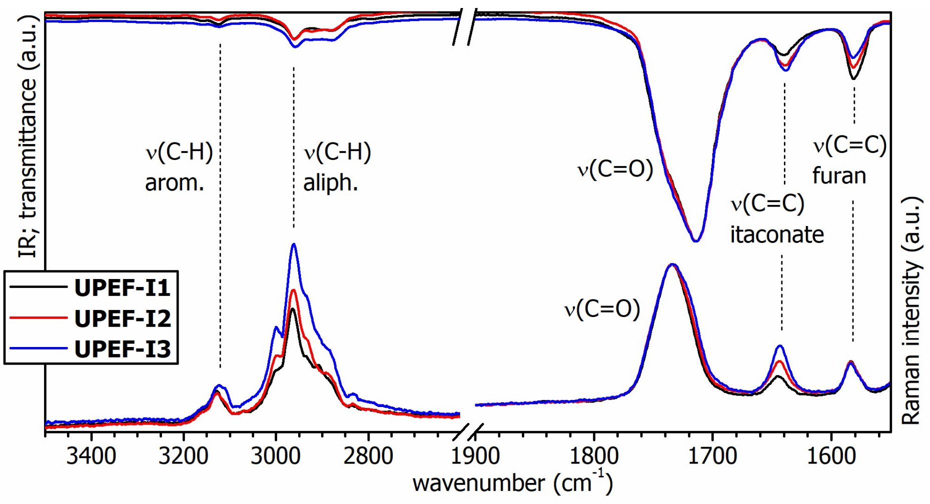

Figure S19. Assignment of vibrational spectra of **UPEF-I1**, **UPEF-I2** and **UPEF-I3**.

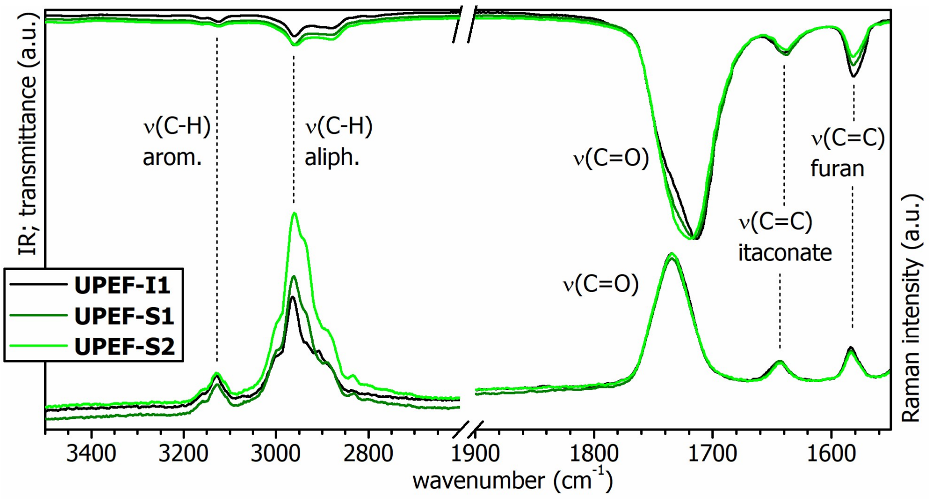

Figure S20. Assignment of vibrational spectra of **UPEF-I1**, **UPEF-S1** and **UPEF-S2**.

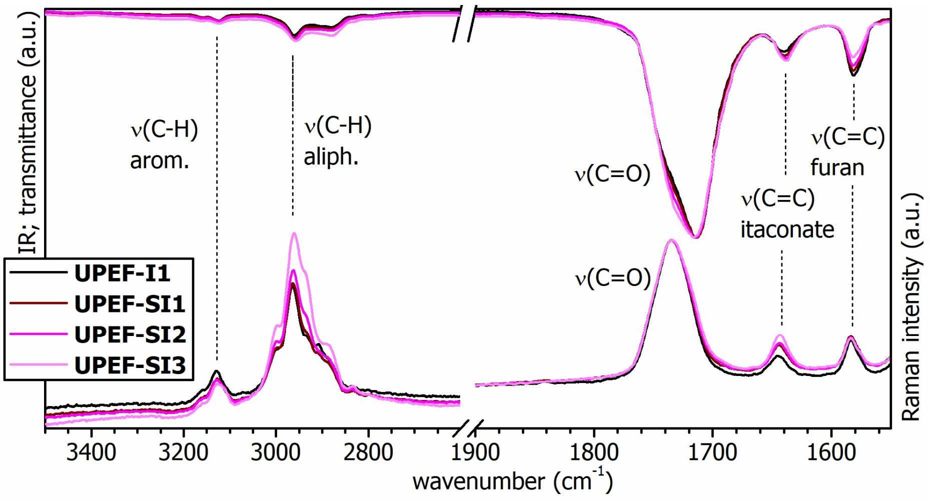

Figure S21. Vibrational of **UPEF-I1**, **UPEF-SI1**, **UPEF-SI2** and **UPET-SI3.**

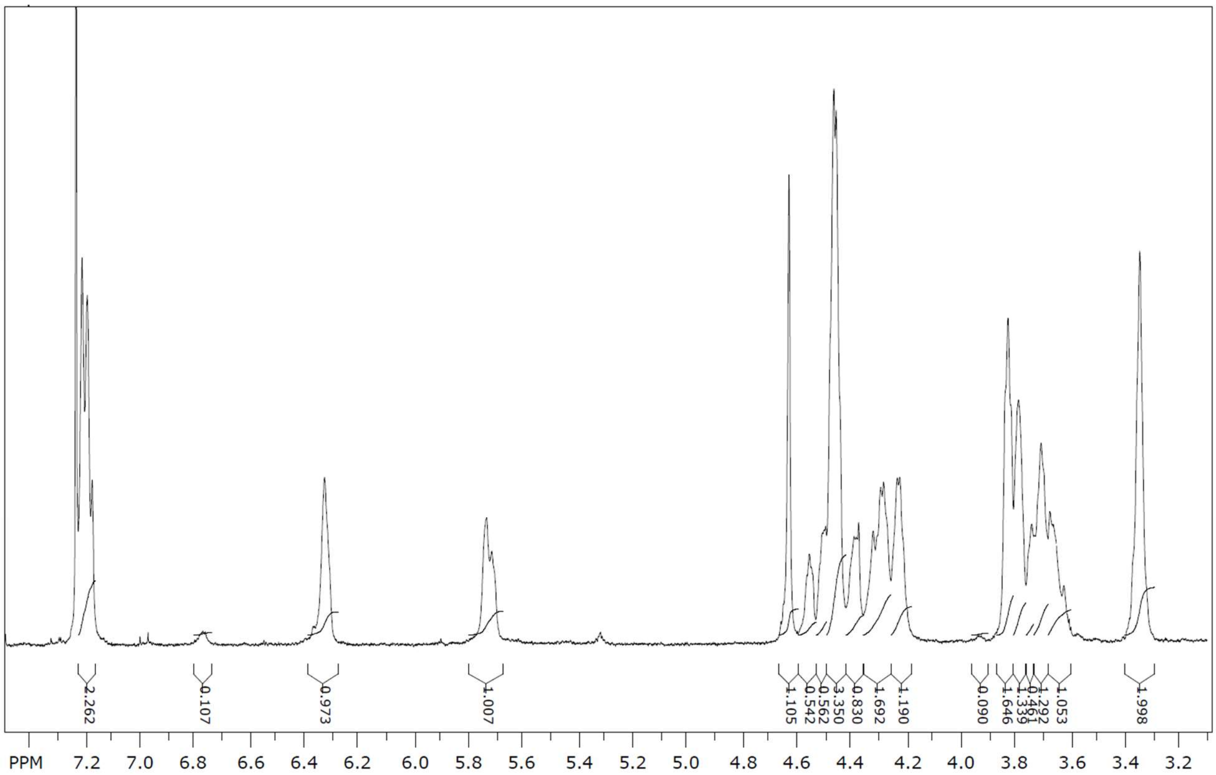

Figure S22. ^{1}H NMR spectrum of **UPEF-I1** in $CDCl_3$.

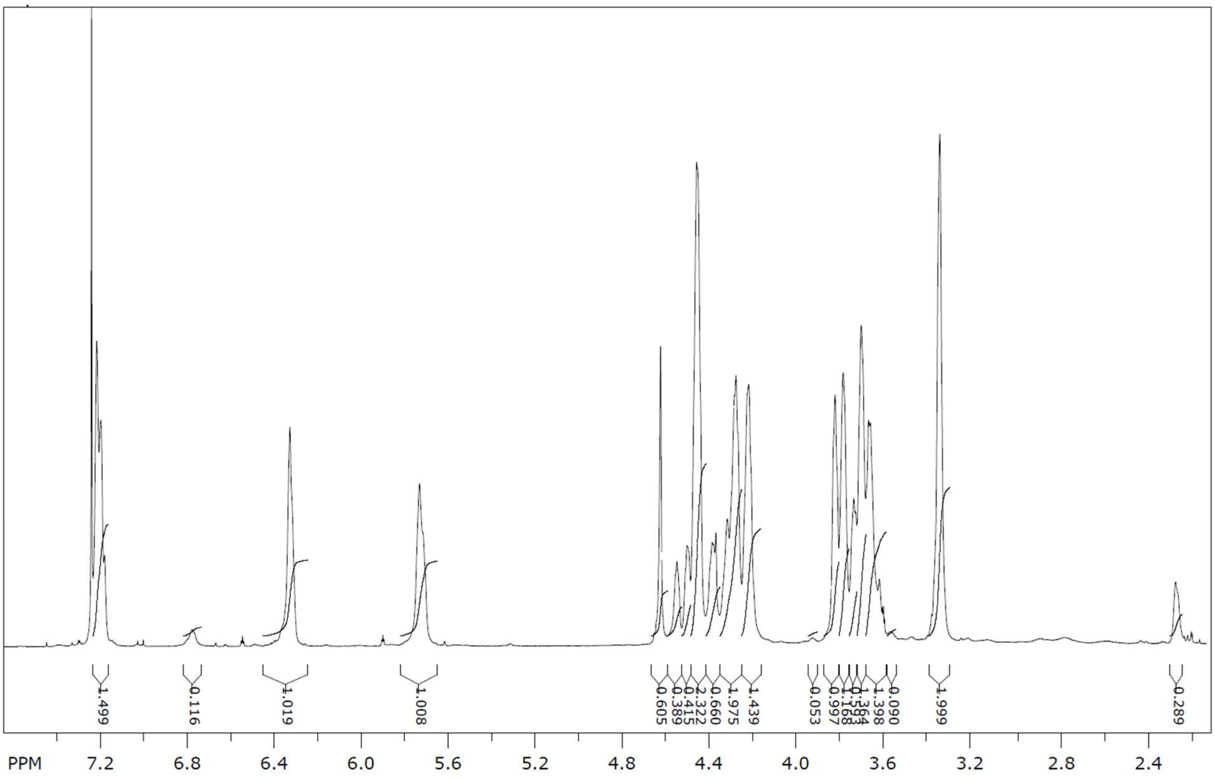

Figure S23. ^{1}H NMR spectrum of **UPEF-I2** in $CDCl_3$.

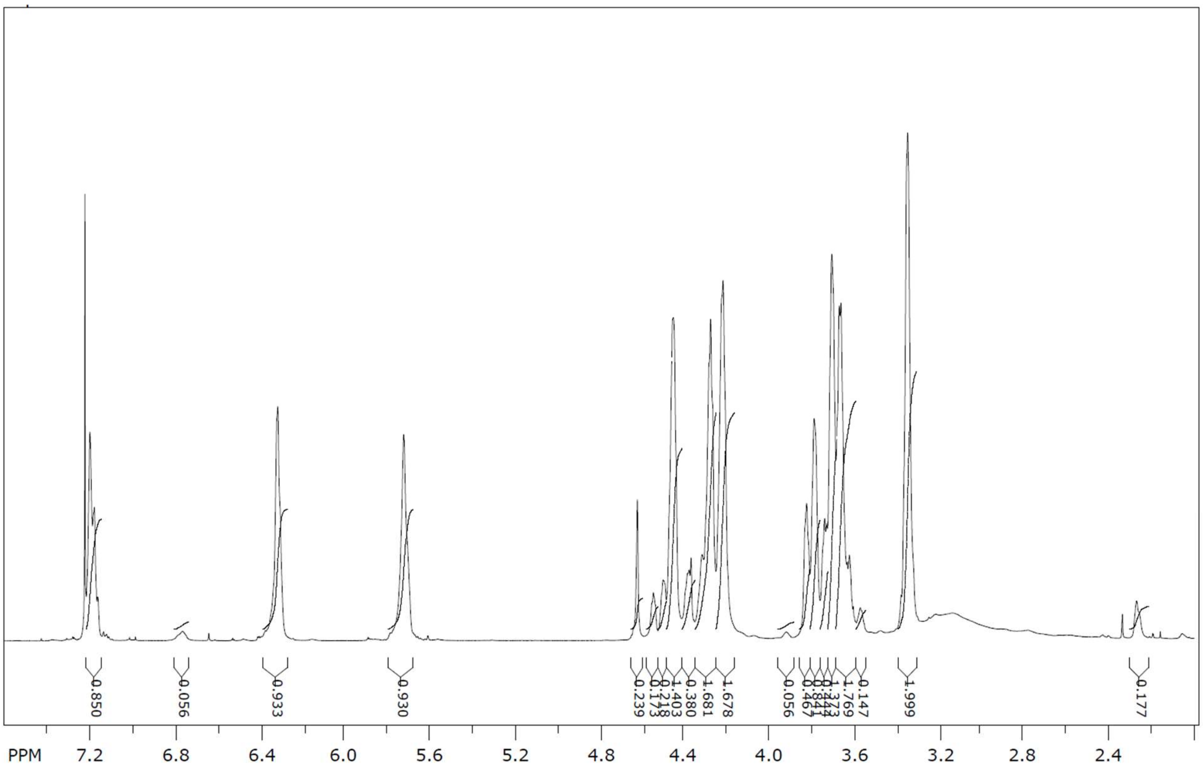

Figure S24. ^{1}H NMR spectrum of **UPEF-I3** in $CDCl_3$.

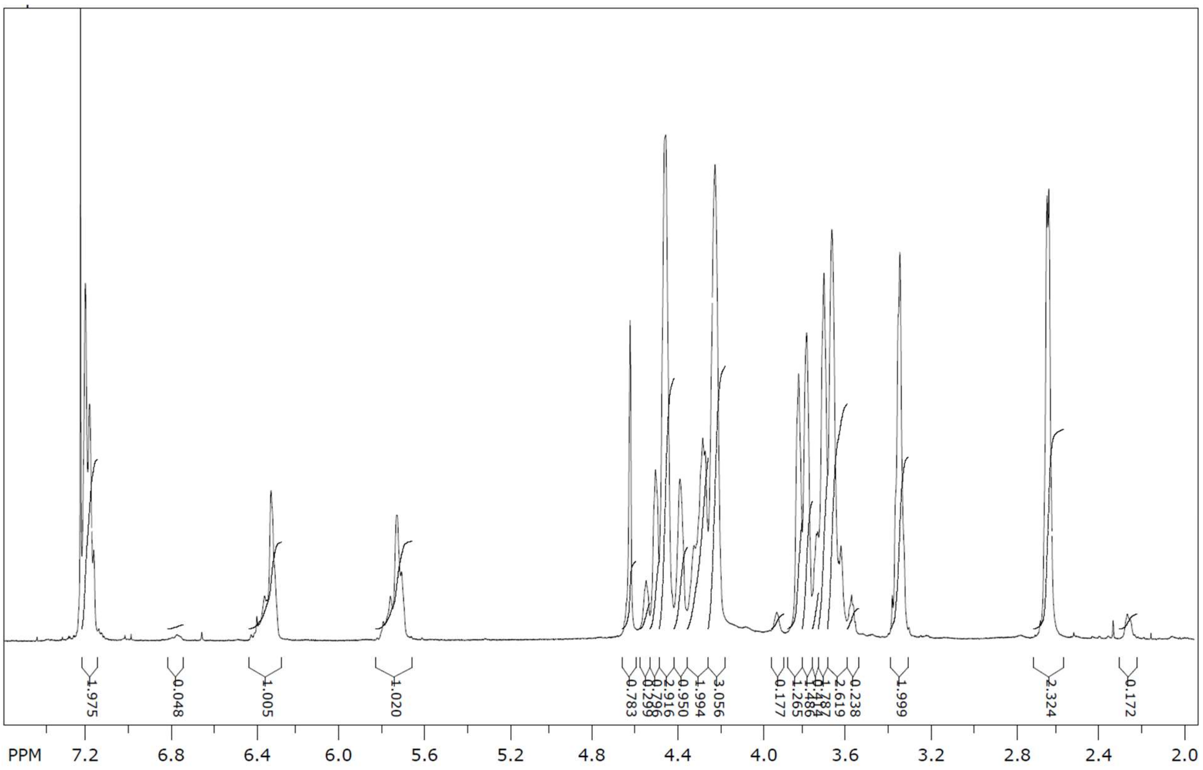

Figure S25. ^{1}H NMR spectrum of **UPEF-S1** in $CDCl_3$.

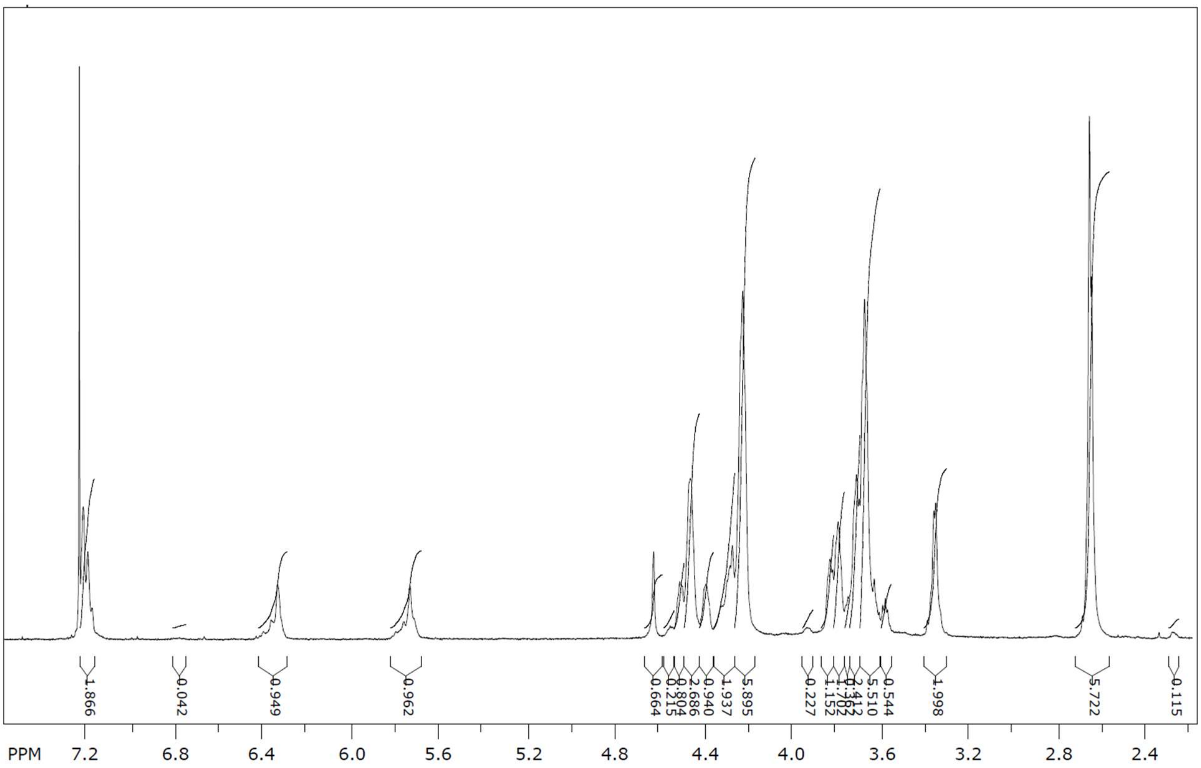

Figure S26. ^{1}H NMR spectrum of **UPEF-S2** in $CDCl_3$.

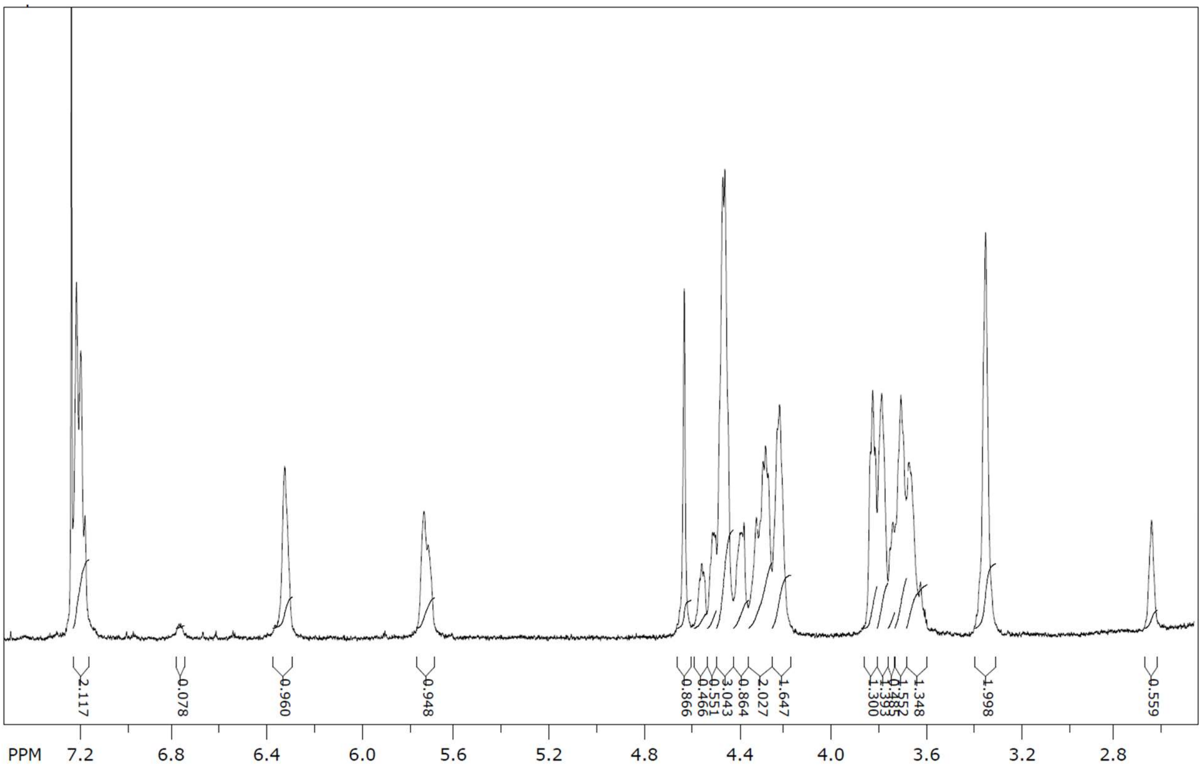

Figure S27. ^{1}H NMR spectrum of **UPEF-SI1** in $CDCl_3$.

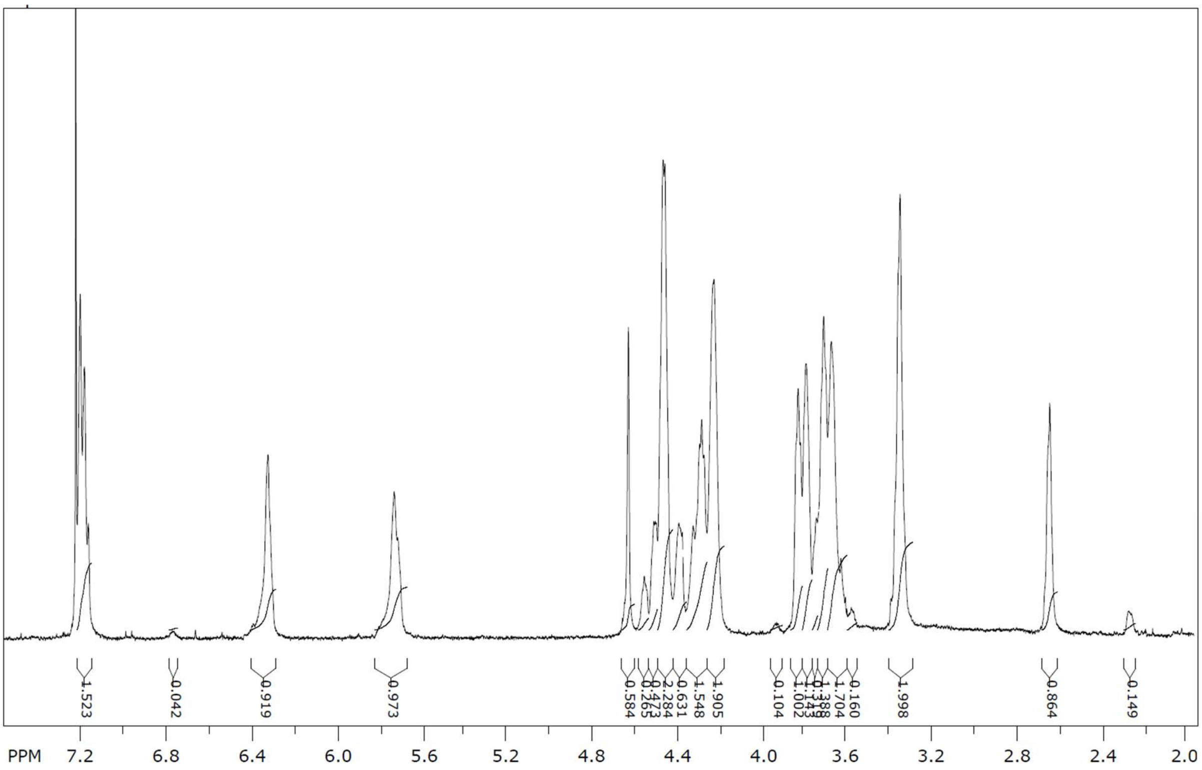

Figure S28. ^{1}H NMR spectrum of **UPEF-SI2** in $CDCl_3$.

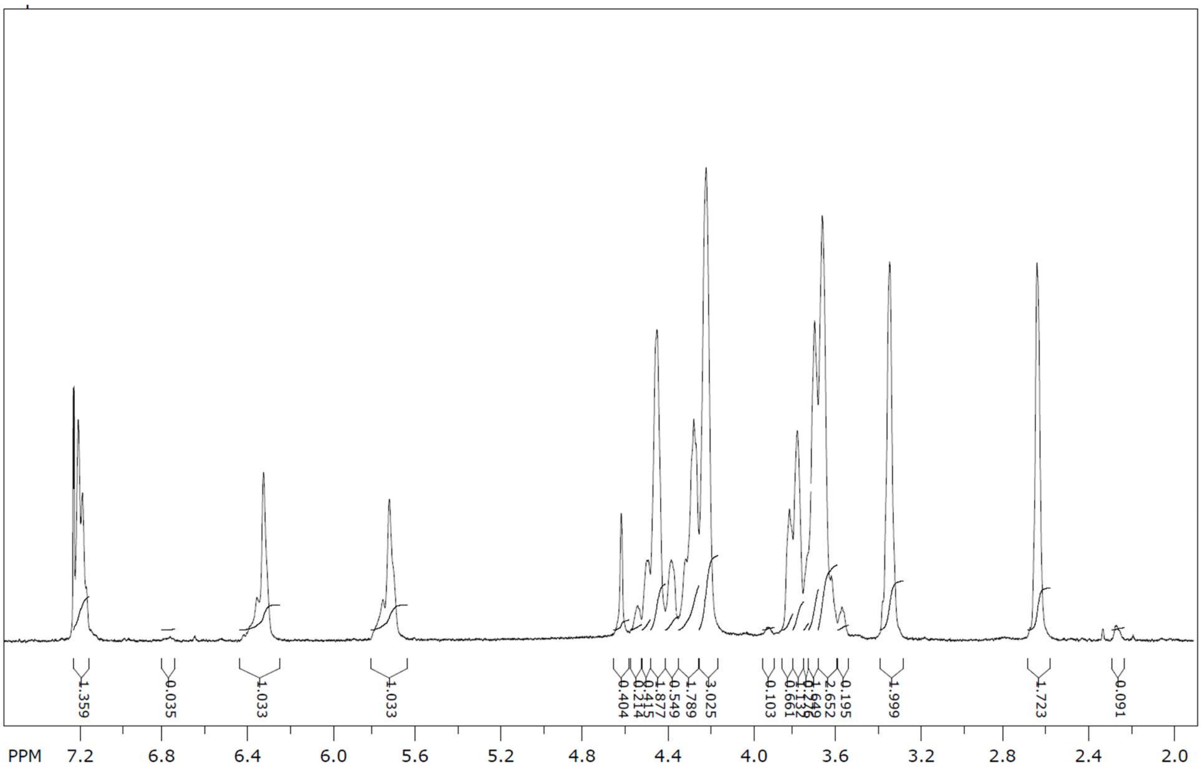

Figure S29. ^{1}H NMR spectrum of **UPEF-SI3** in $CDCl_3$.

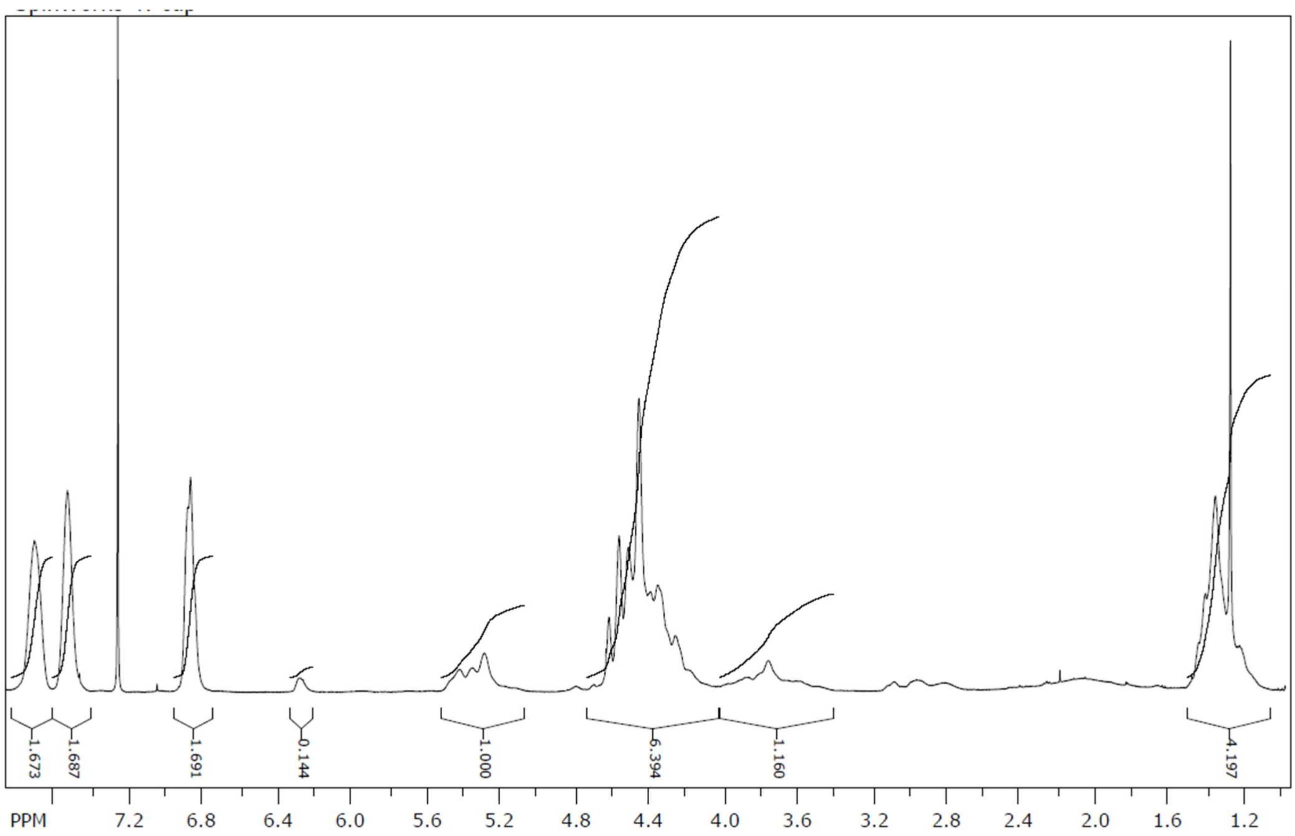

Figure S30. ^{1}H NMR spectrum of **UPR-F** in $CDCl_3$.

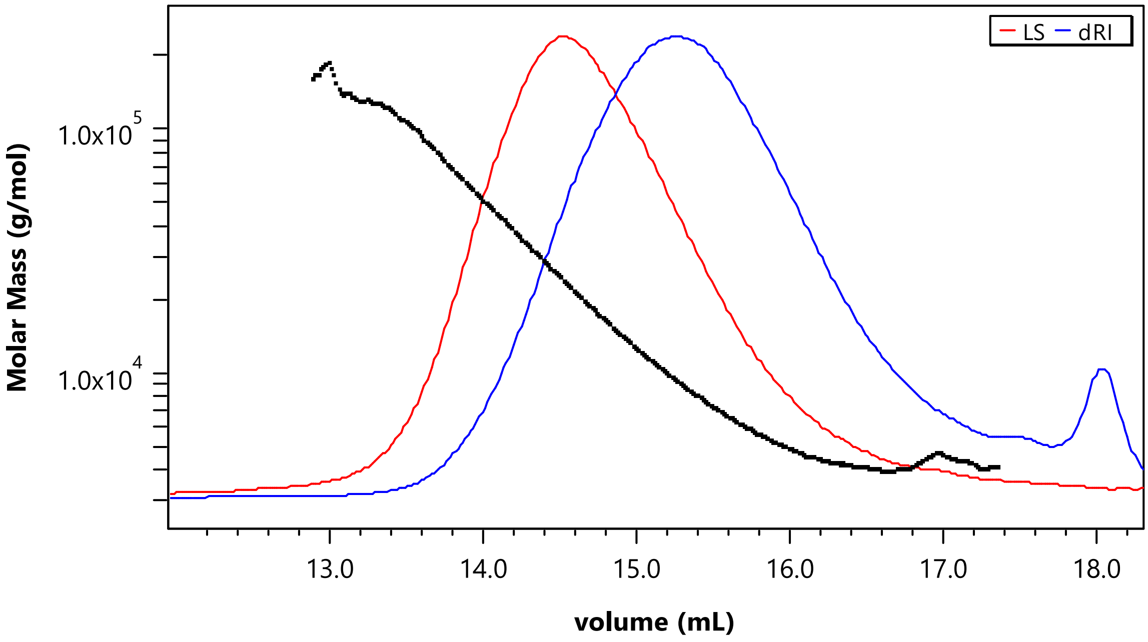

Figure S31. Molar mass versus elution volume plot and chromatograms recorded by RI detector (blue) and MALS detector at 90° (red) of **UPEF-I1**.

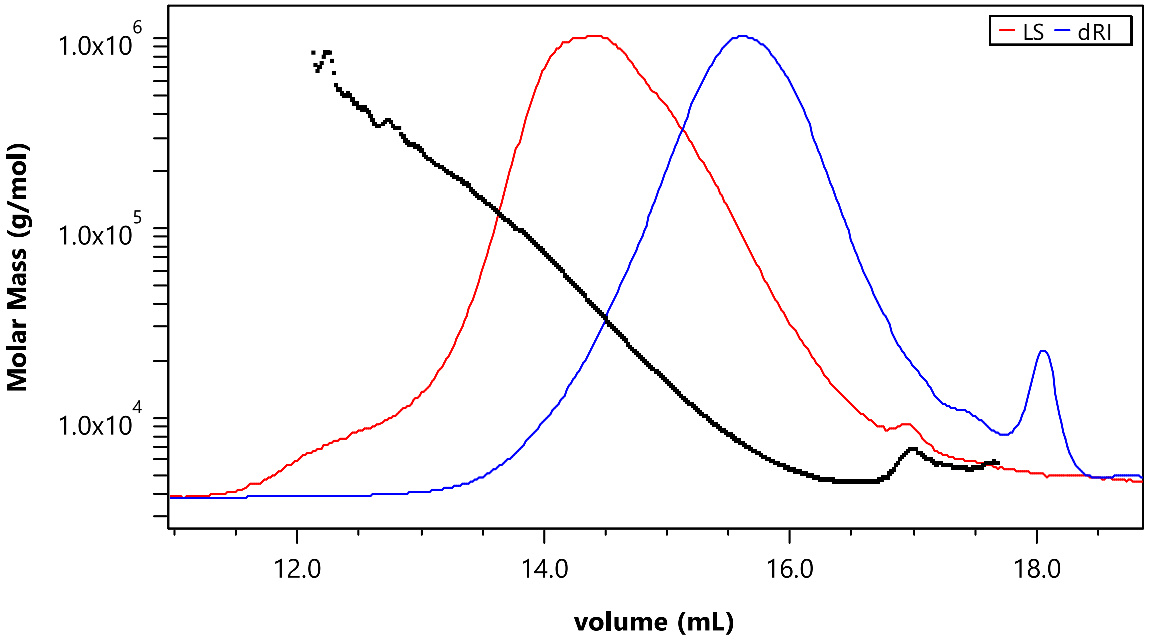

Figure S32. Molar mass versus elution volume plot and chromatograms recorded by RI detector (blue) and MALS detector at 90° (red) of **UPEF-I2**.

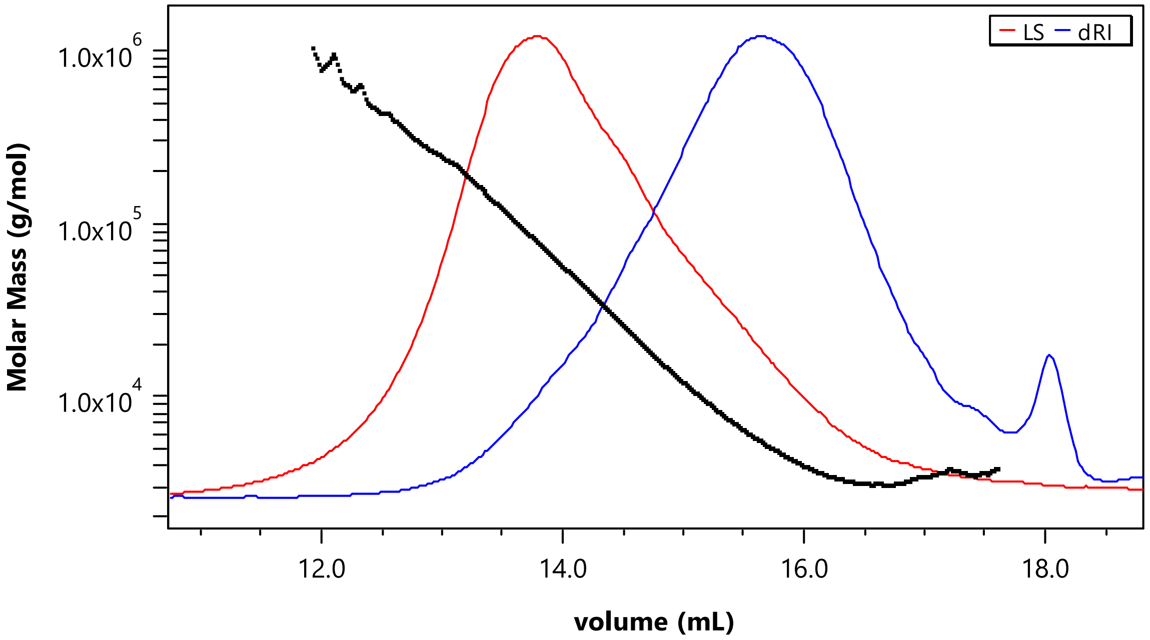

Figure S33. Molar mass versus elution volume plot and chromatograms recorded by RI detector (blue) and MALS detector at 90° (red) of **UPEF-I3**.

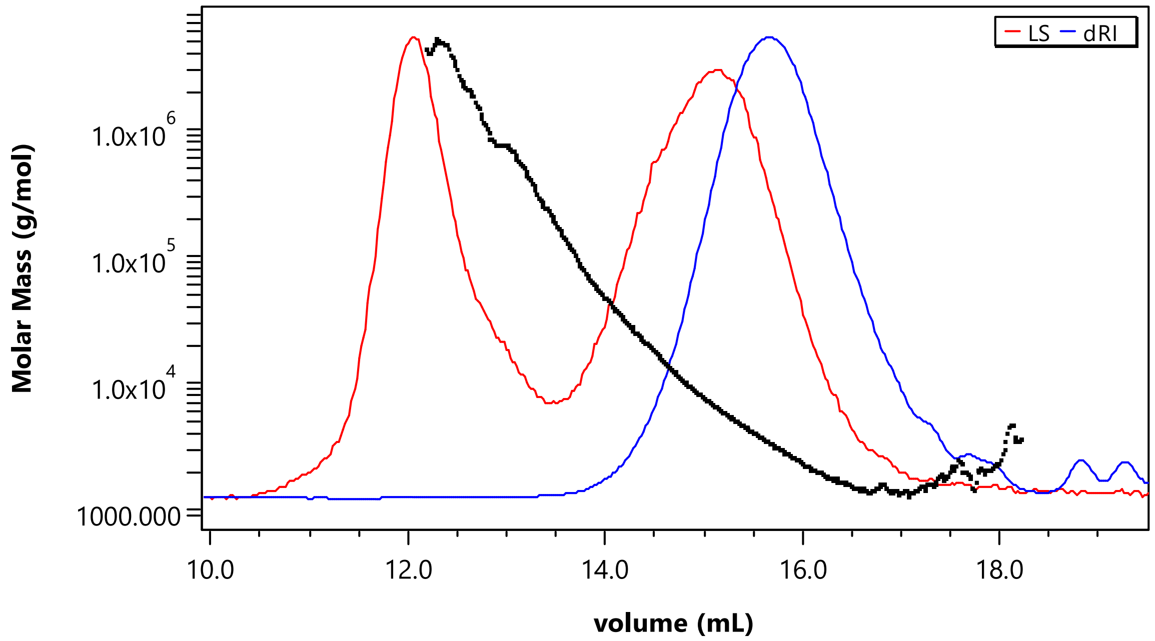

Figure S34. Molar mass versus elution volume plot and chromatograms recorded by RI detector (blue) and MALS detector at 90° (red) of **UPEF-S1**.

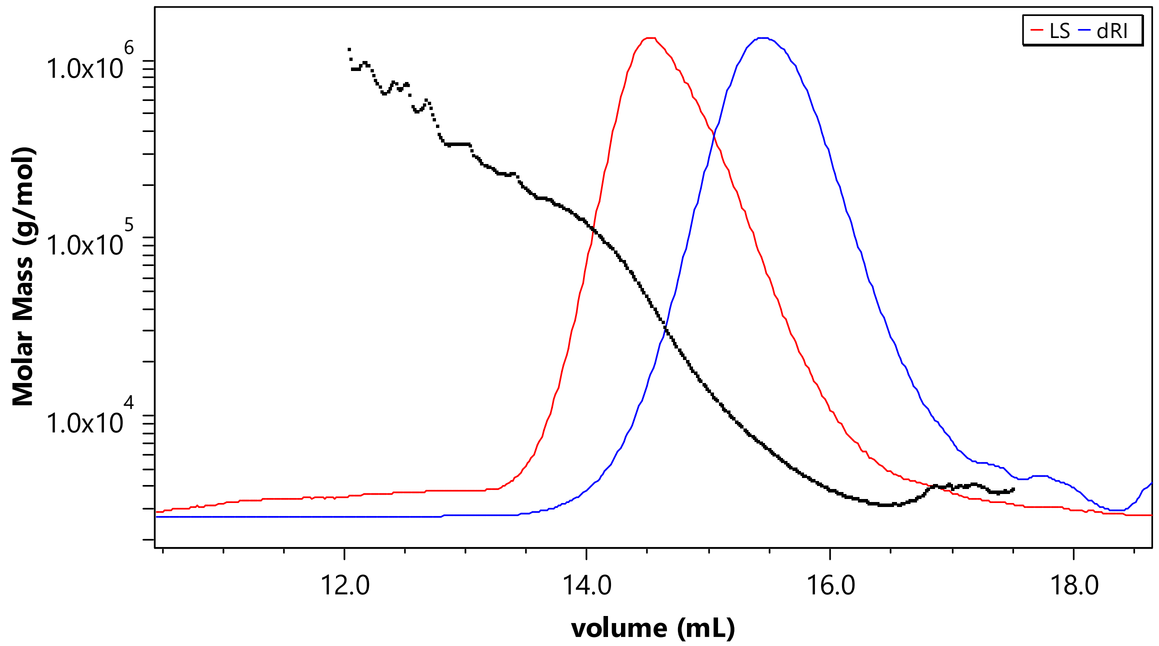

Figure S35. Molar mass versus elution volume plot and chromatograms recorded by RI detector (blue) and MALS detector at 90° (red) of **UPEF-S2**.

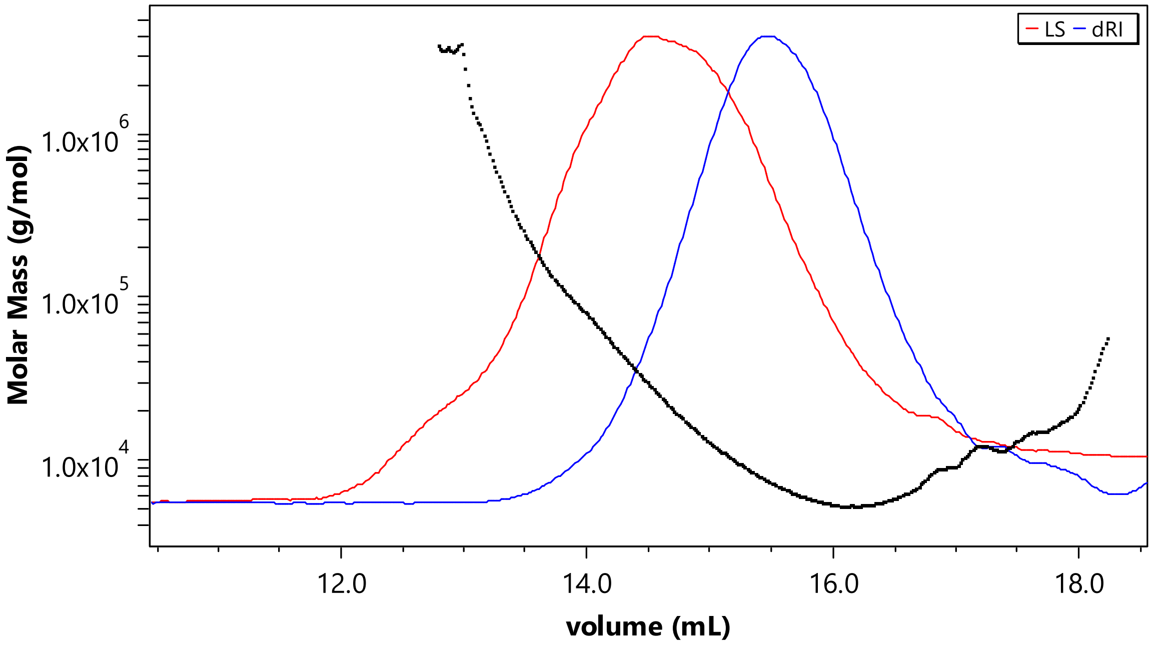

Figure S36. Molar mass versus elution volume plot and chromatograms recorded by RI detector (blue) and MALS detector at 90° (red) of **UPEF-SI1**.

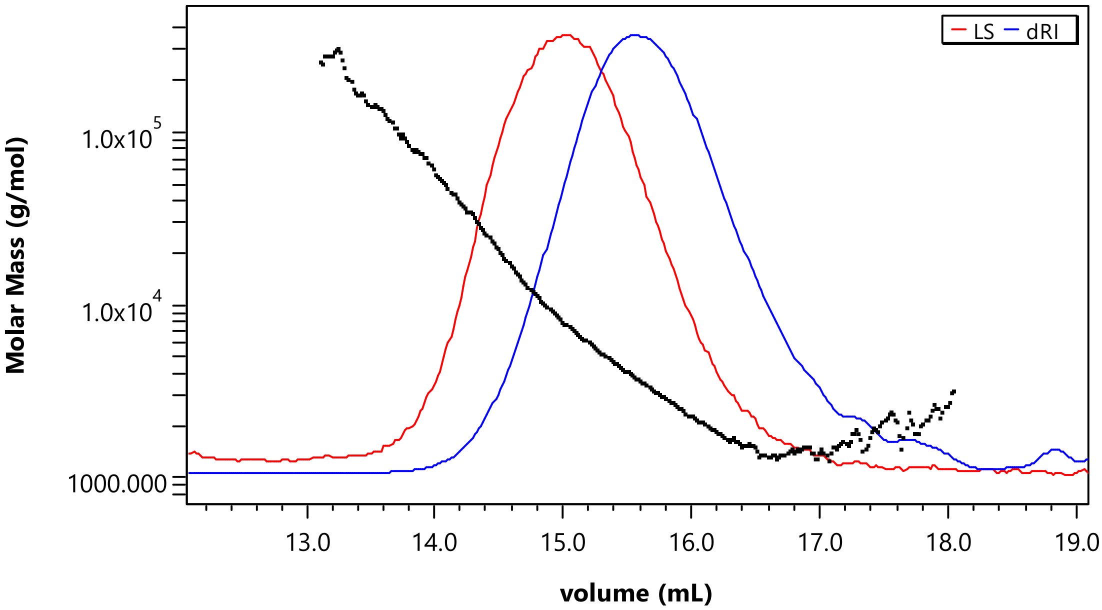

Figure S37. Molar mass versus elution volume plot and chromatograms recorded by RI detector (blue) and MALS detector at 90° (red) of **UPEF-SI2**.

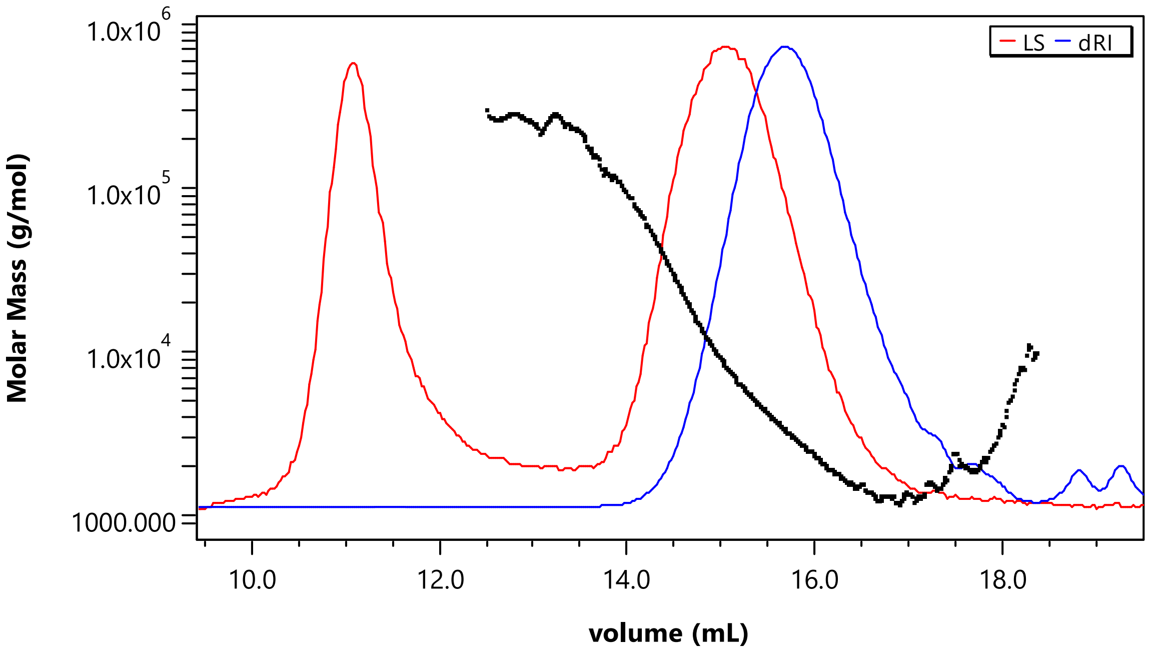

Figure S38. Molar mass versus elution volume plot and chromatograms recorded by RI detector (blue) and MALS detector at 90° (red) of **UPEF-SI3**.

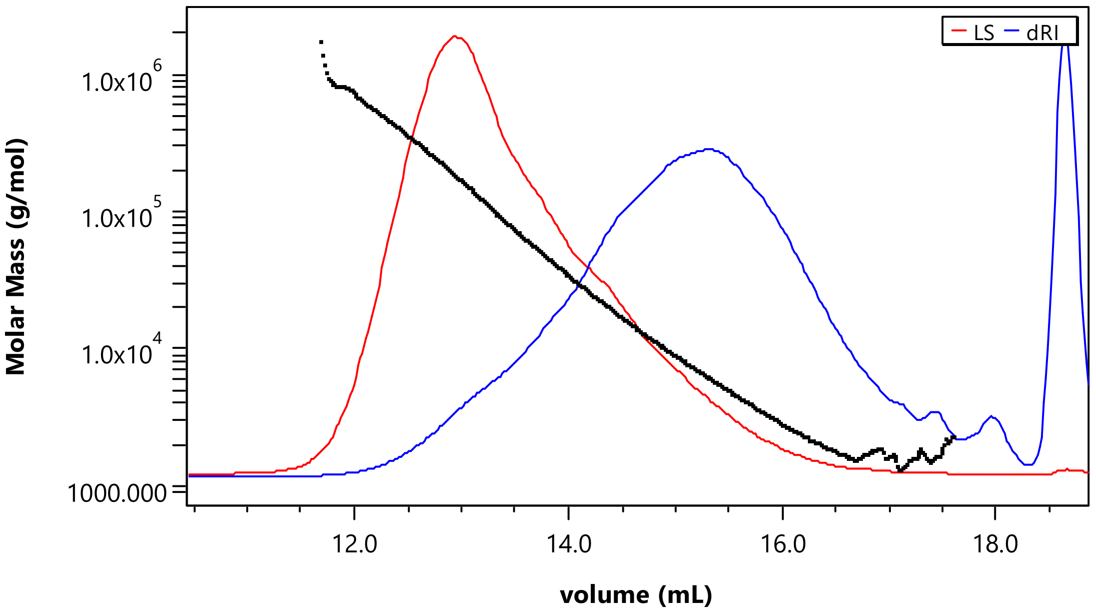

Figure S39. Molar mass versus elution volume plot and chromatograms recorded by RI detector (blue) and MALS detector at 90° (red) of **UPR-F**.

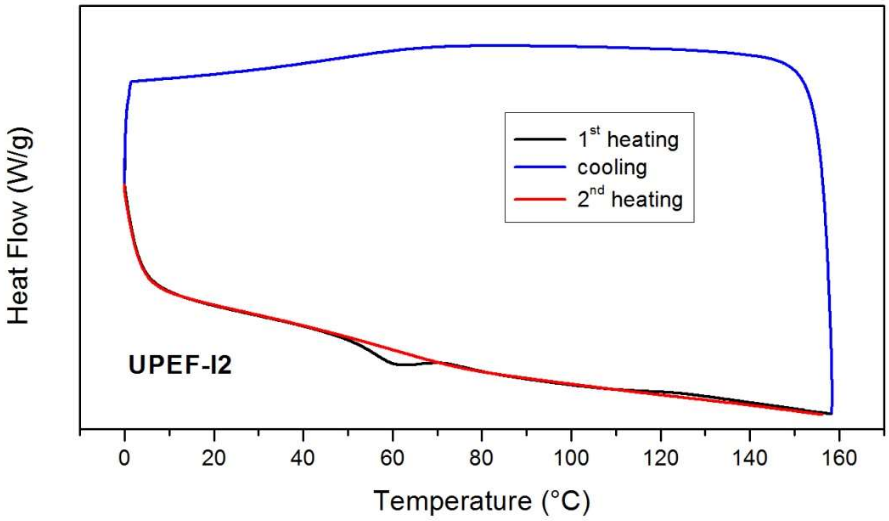

Figure S40. DSC curves of **UPEF-I2**.

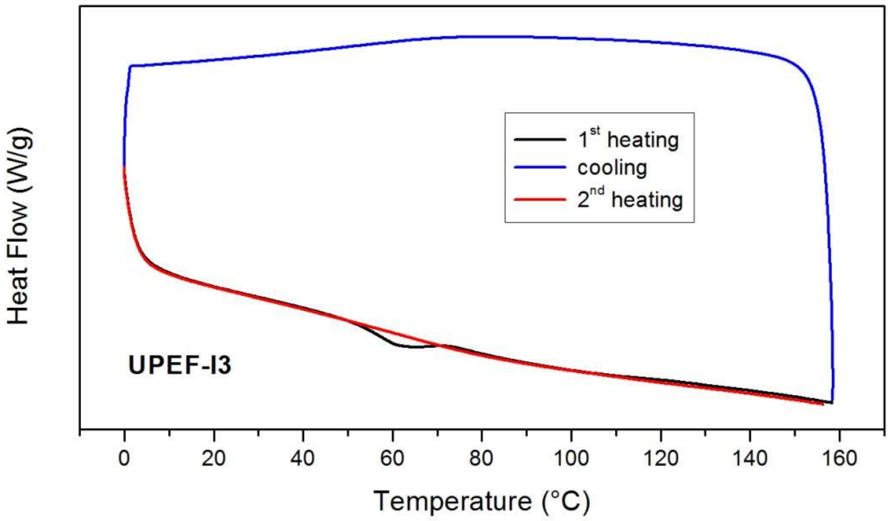

Figure S41. DSC curves of **UPEF-I3**.

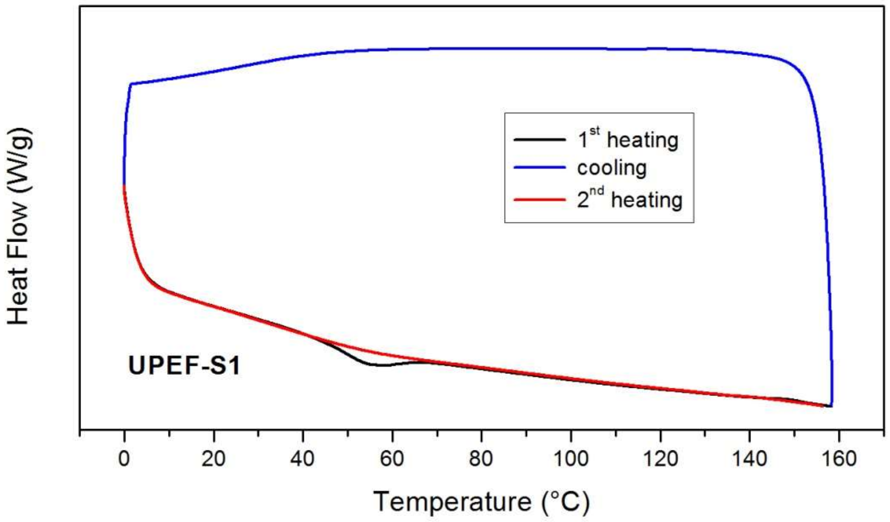

Figure S42. DSC curves of **UPEF-S1**.

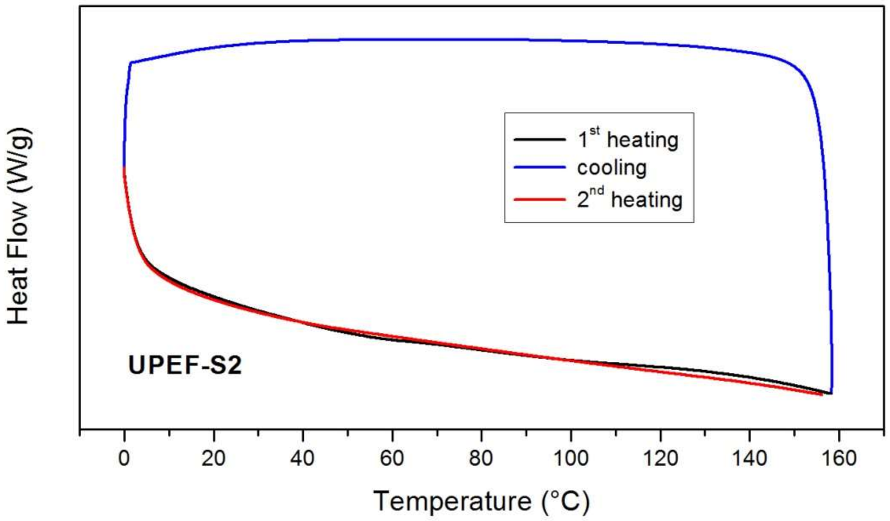

Figure S43. DSC curves of **UPEF-S2**.

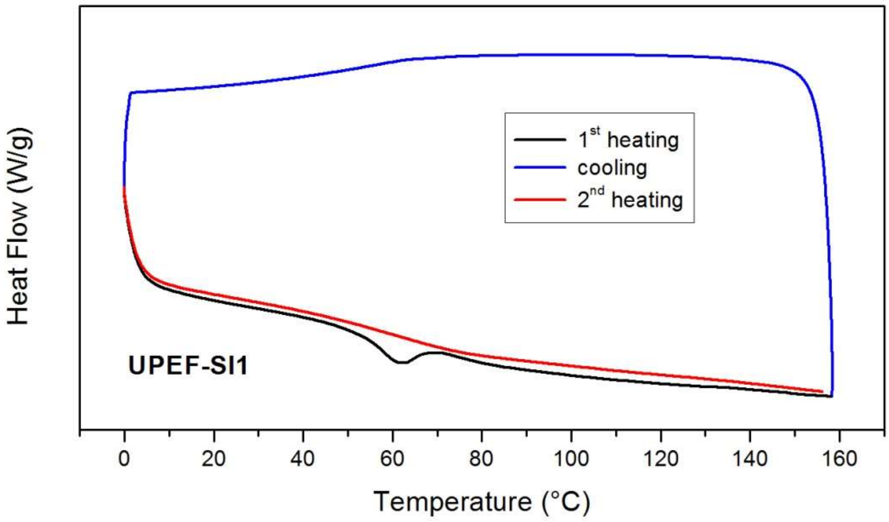

Figure S44. DSC curves of **UPEF-SI1**.

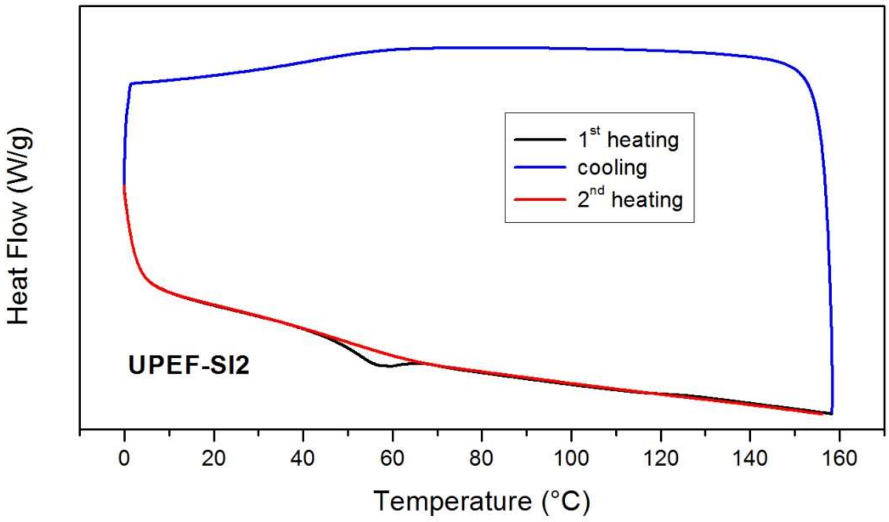

Figure S45. DSC curves of **UPEF-SI2**.

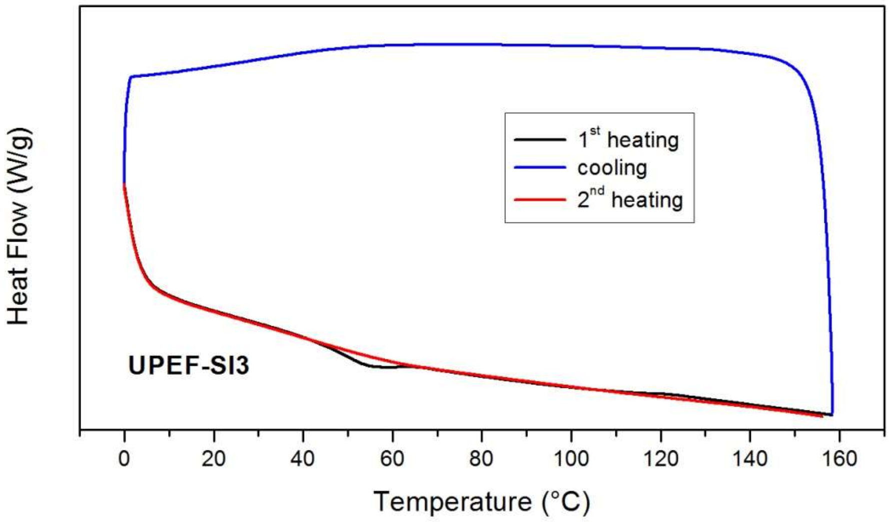

Figure S46. DSC curves of **UPEF-SI3**.

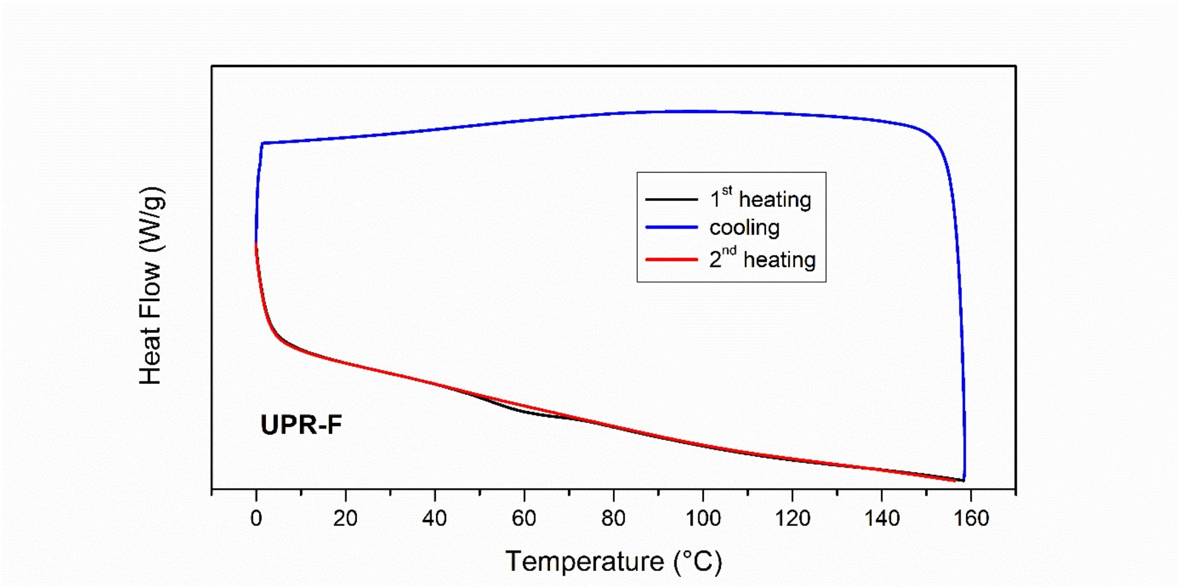

Figure S47. DSC curves of **UPR-F**.

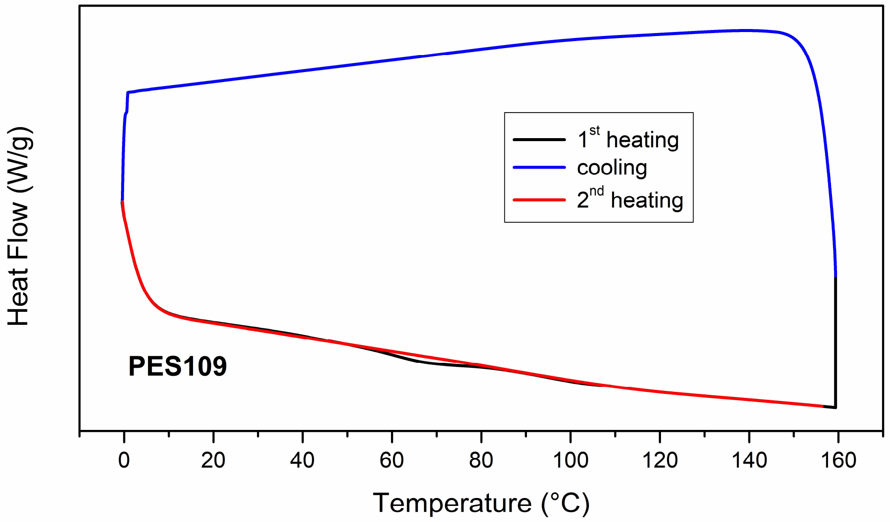

Figure S48. DSC curves of **PES109**.

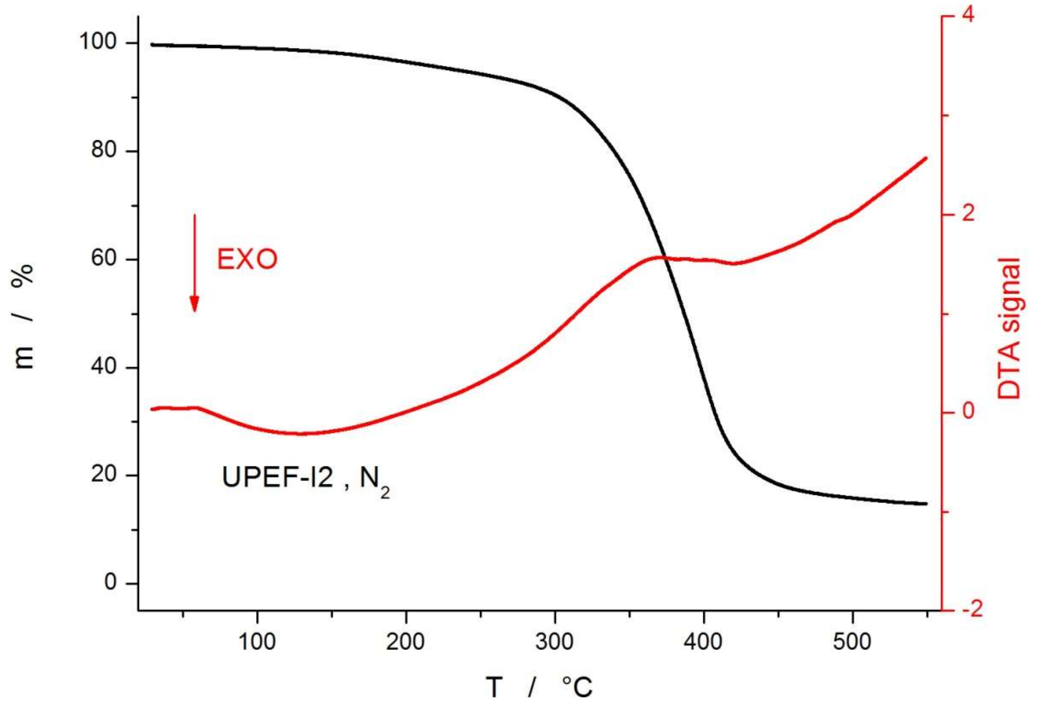

Figure S49. TGA measurement of **UPEF-I2** in nitrogen atmosphere.

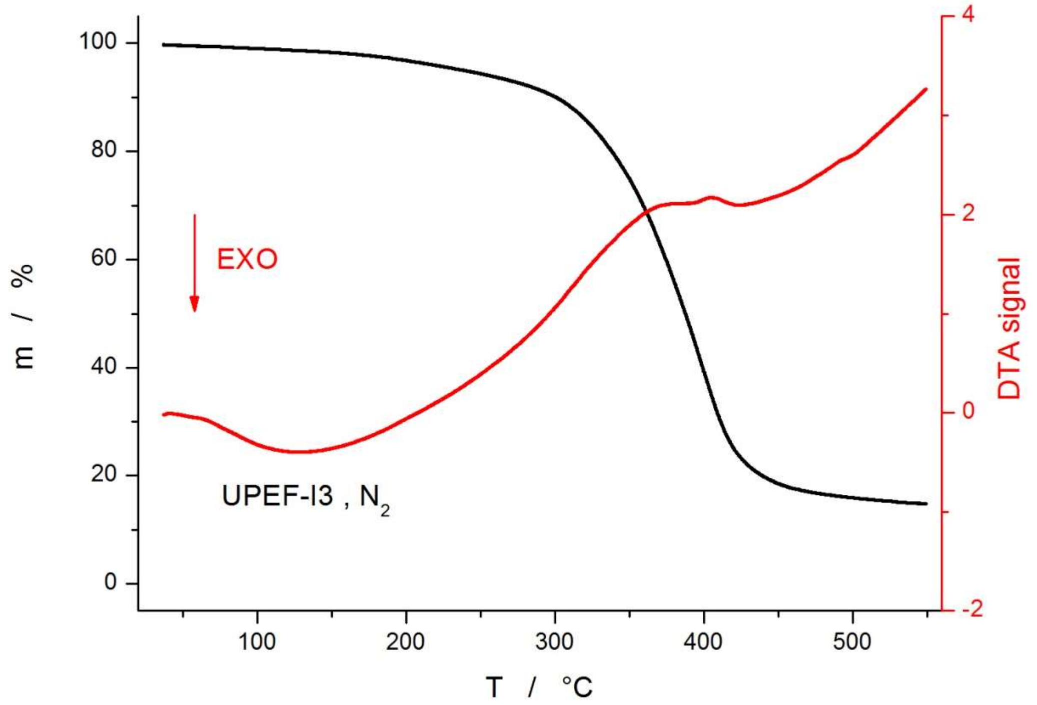

Figure S50. TGA measurement of **UPEF-I3** in nitrogen atmosphere.

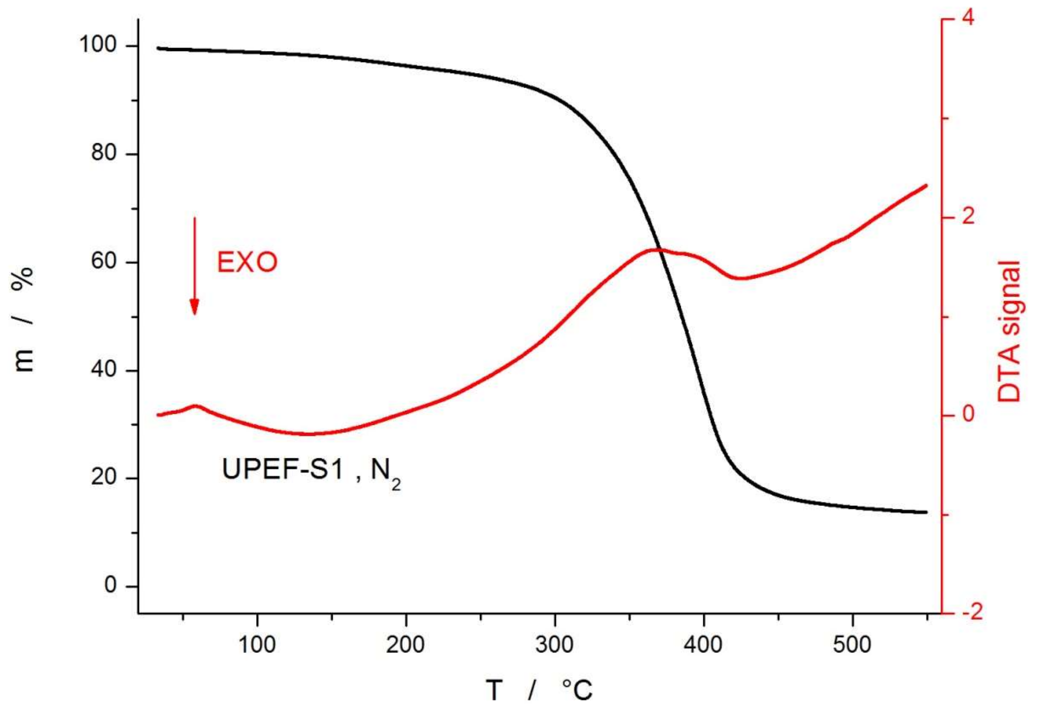

Figure S51. TGA measurement of **UPEF-S1** in nitrogen atmosphere.

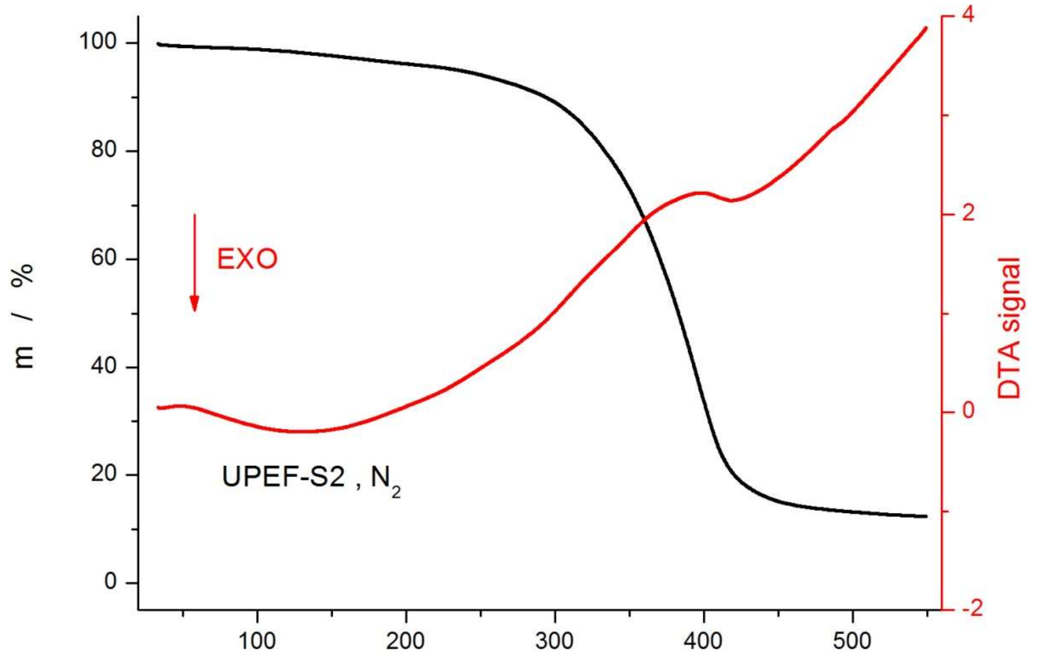

Figure S52. TGA measurement of **UPEF-S2** in nitrogen atmosphere.

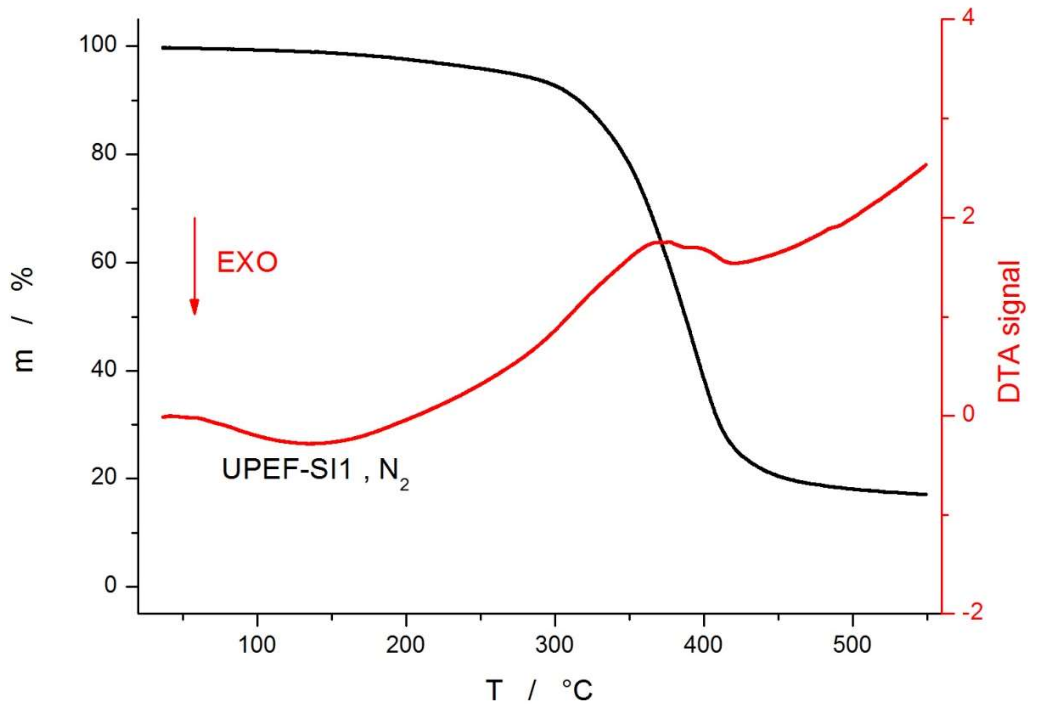

Figure S53. TGA measurement of **UPEF-SI1** in nitrogen atmosphere.

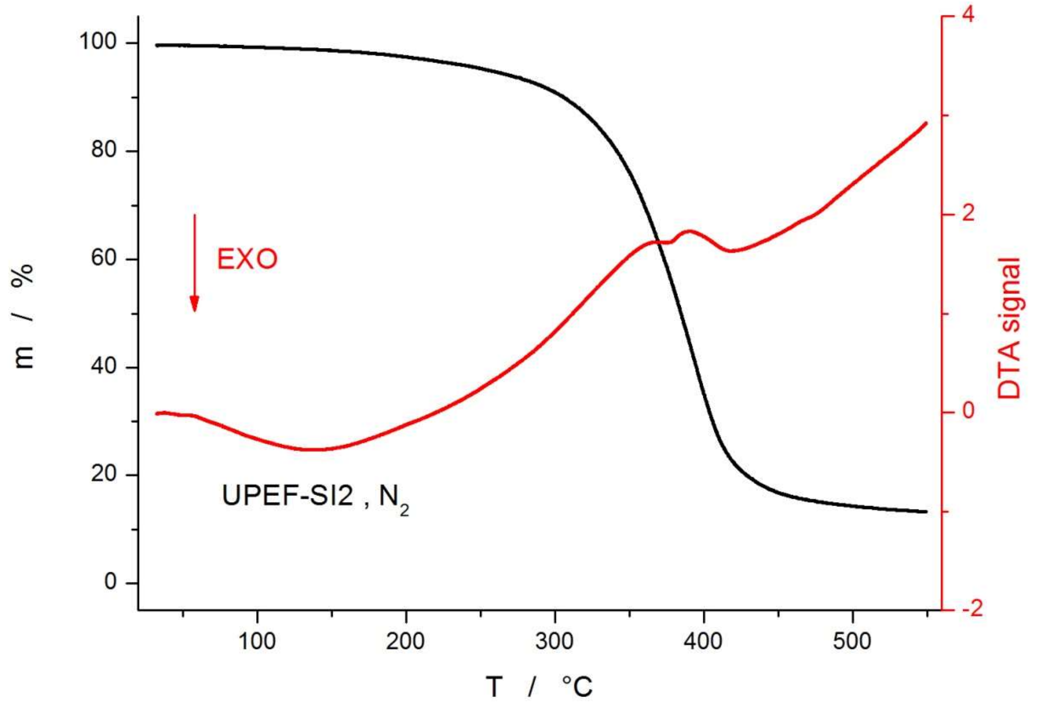

Figure S54. TGA measurement of **UPEF-SI2** in nitrogen atmosphere.

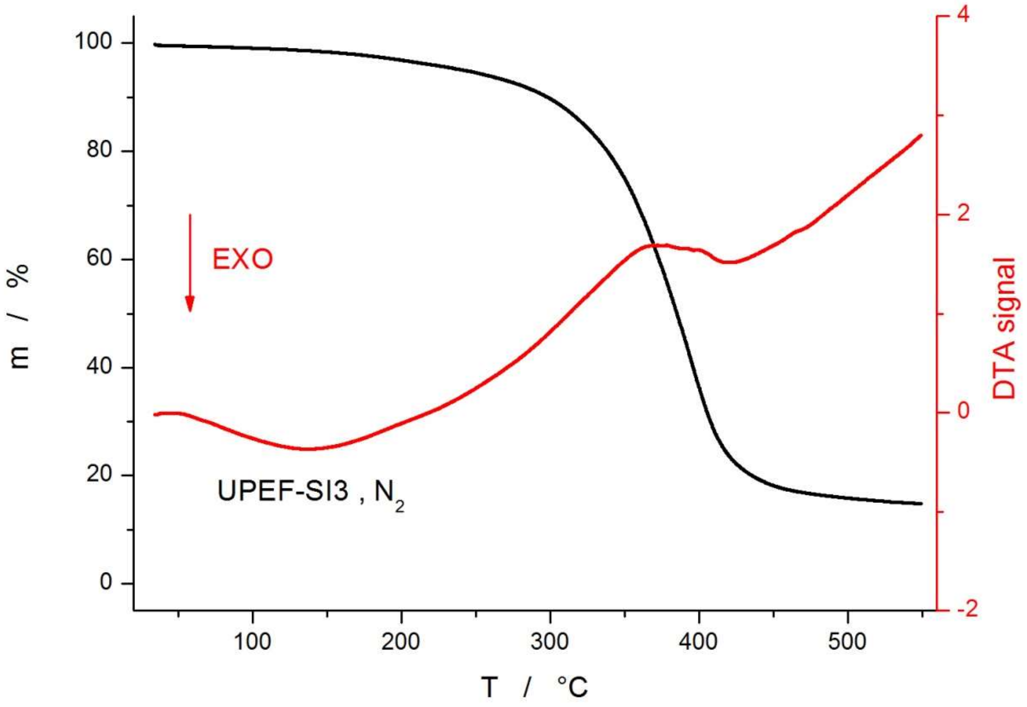

Figure S55. TGA measurement of **UPEF-SI3** in nitrogen atmosphere.

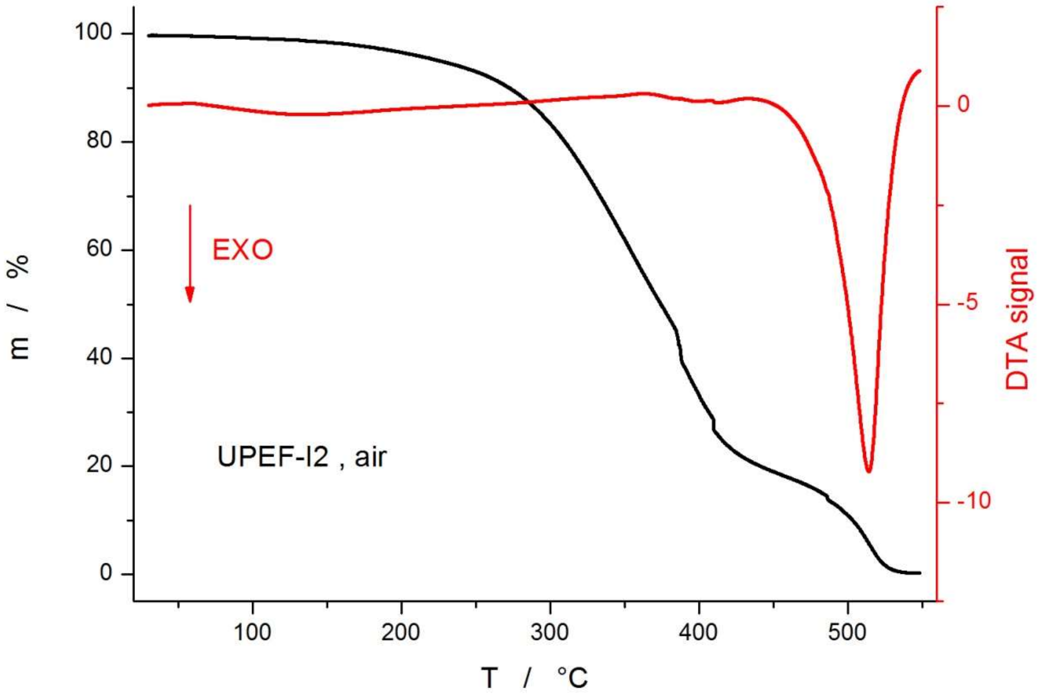

Figure S56. TGA measurement of **UPEF-I2** in air atmosphere.

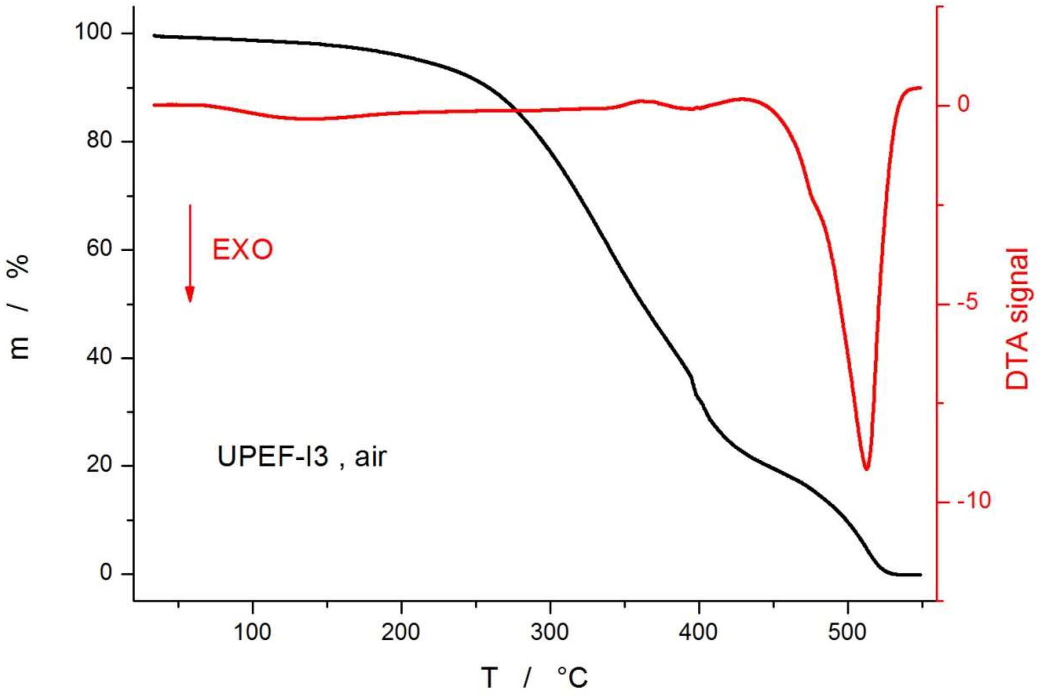

Figure S57. TGA measurement of **UPEF-I3** in air atmosphere.

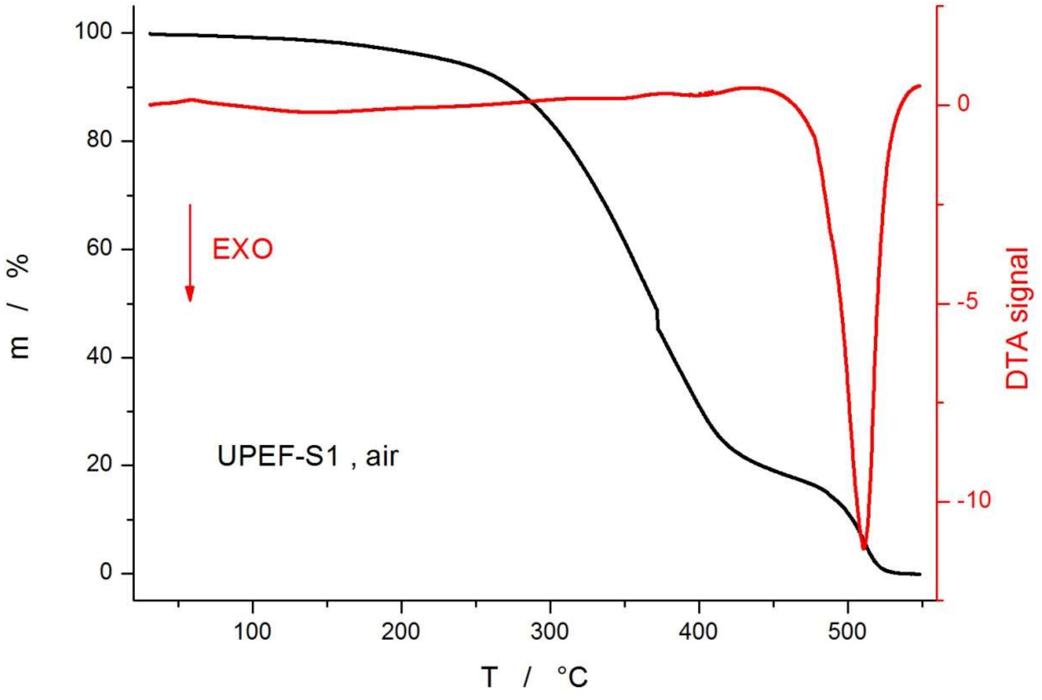

Figure S58. TGA measurement of **UPEF-S1** in air atmosphere.

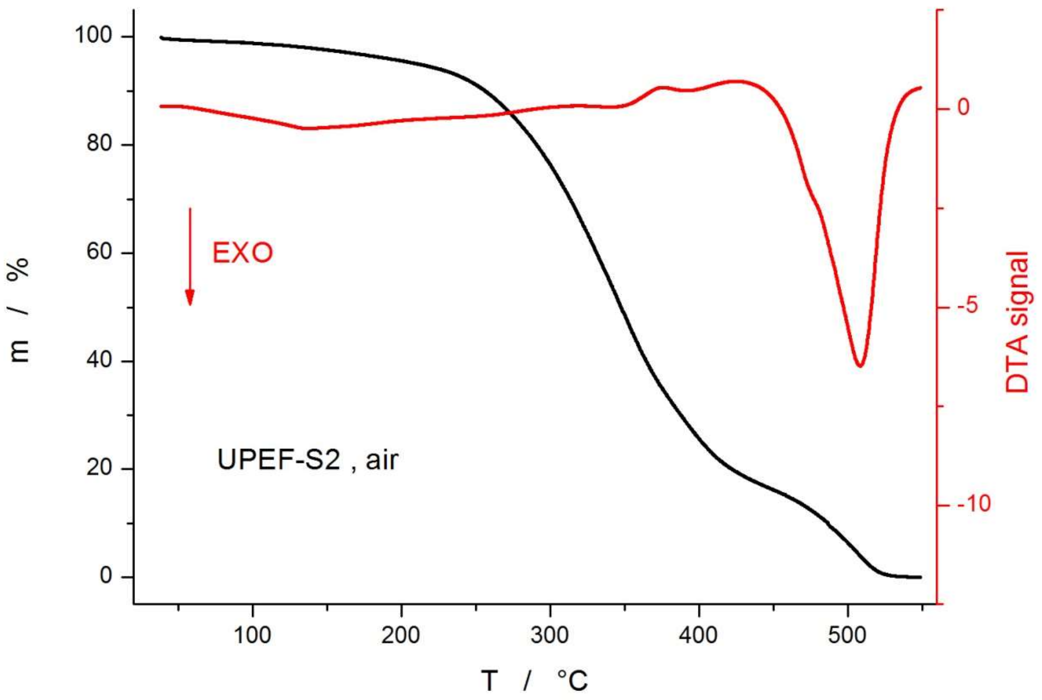

Figure S59. TGA measurement of **UPEF-S2** in air atmosphere.

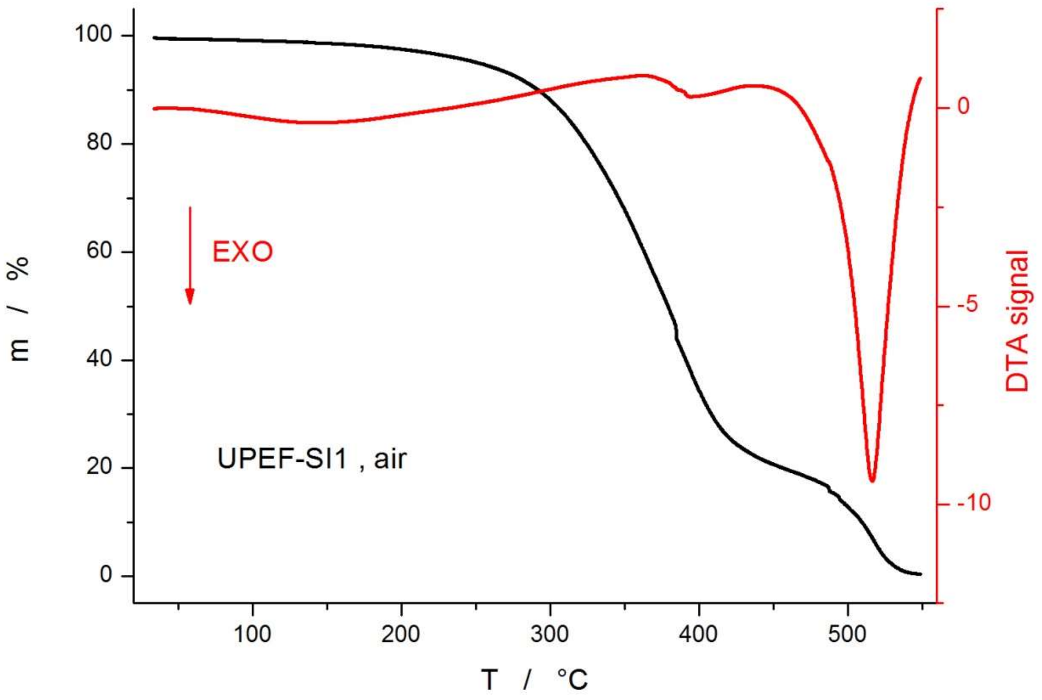

Figure S60. TGA measurement of **UPEF-SI1** in air atmosphere.

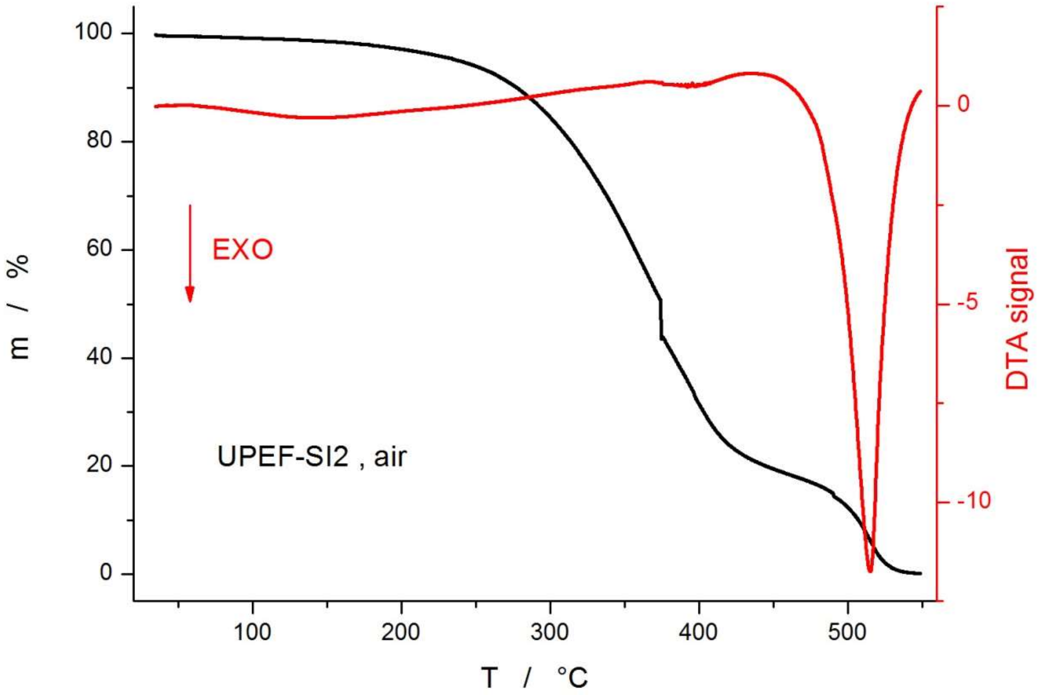

Figure S61. TGA measurement of **UPEF-SI2** in air atmosphere.

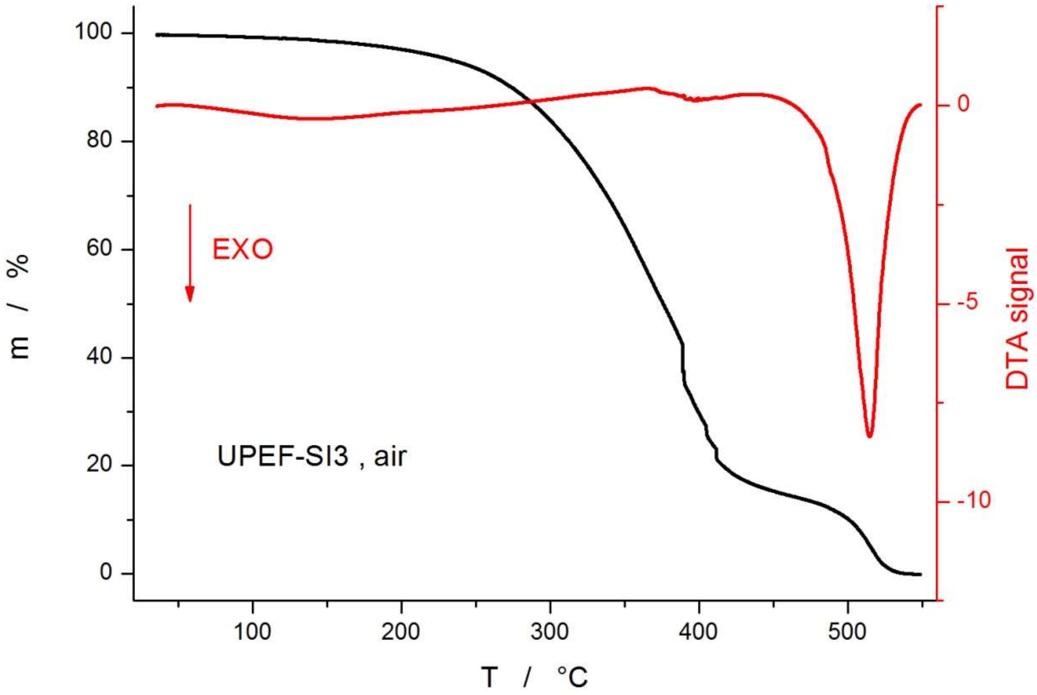

Figure S62. TGA measurement of **UPEF-SI3** in air atmosphere.

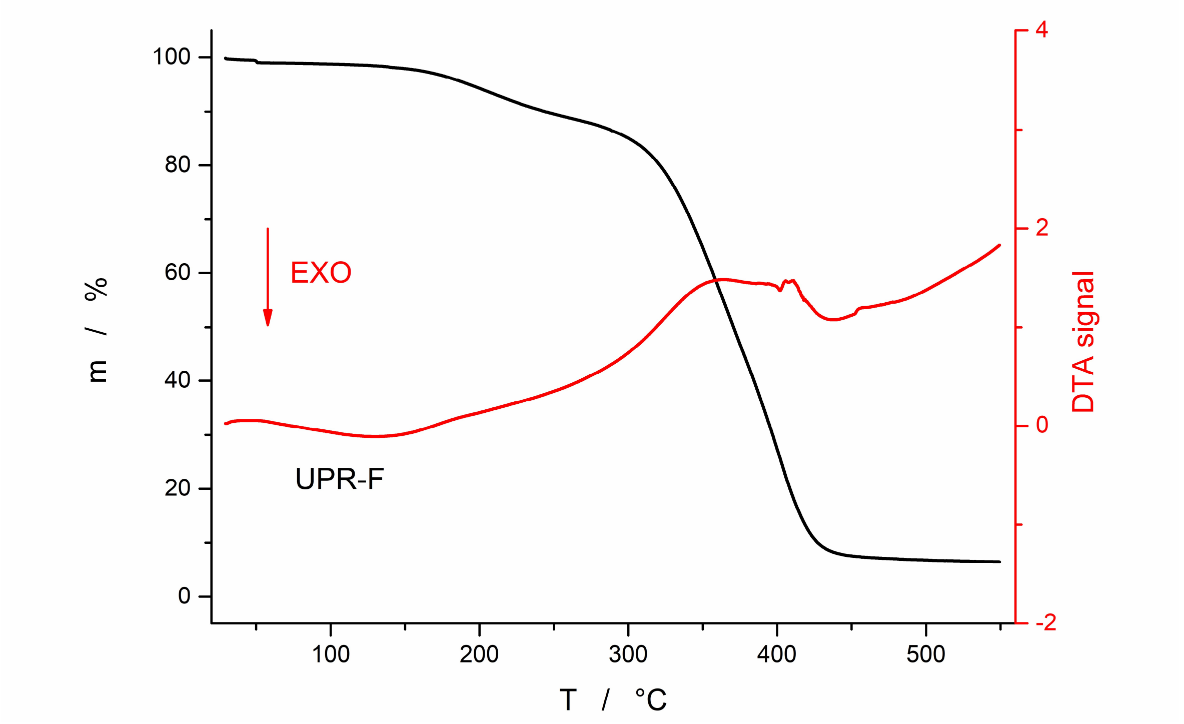

Figure S63. TGA measurement of **UPR-F** in nitrogen atmosphere.

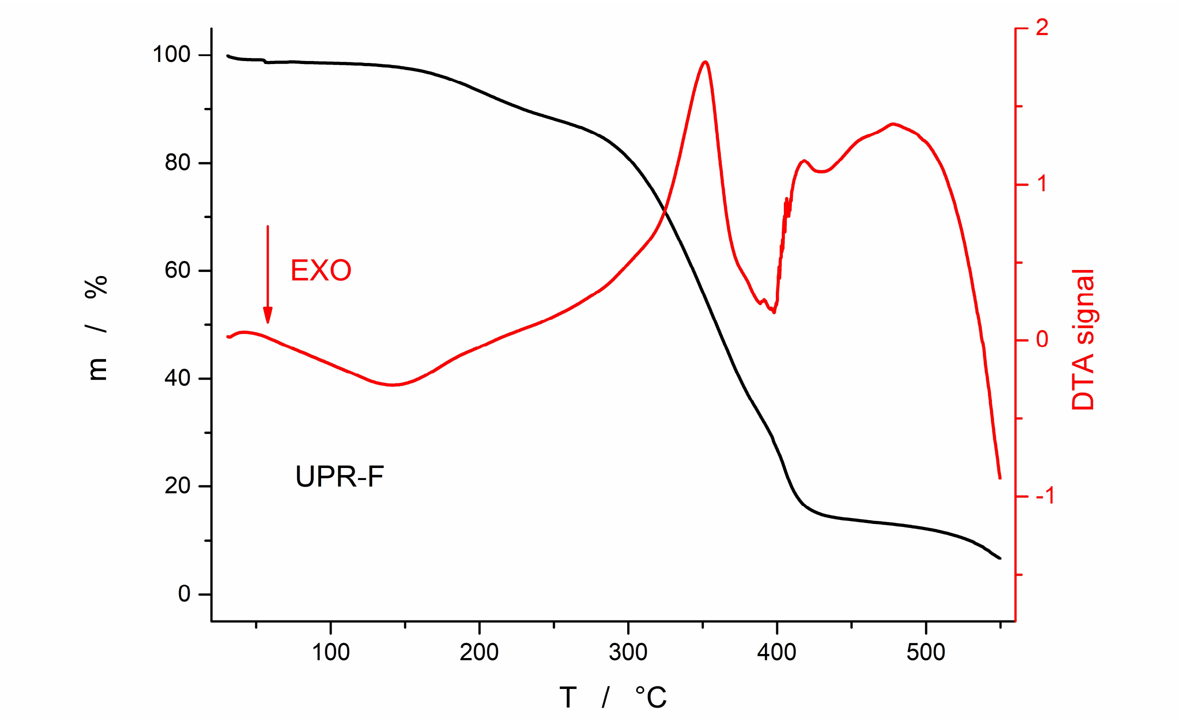

Figure S64. TGA measurement of **UPR-F** in air atmosphere.

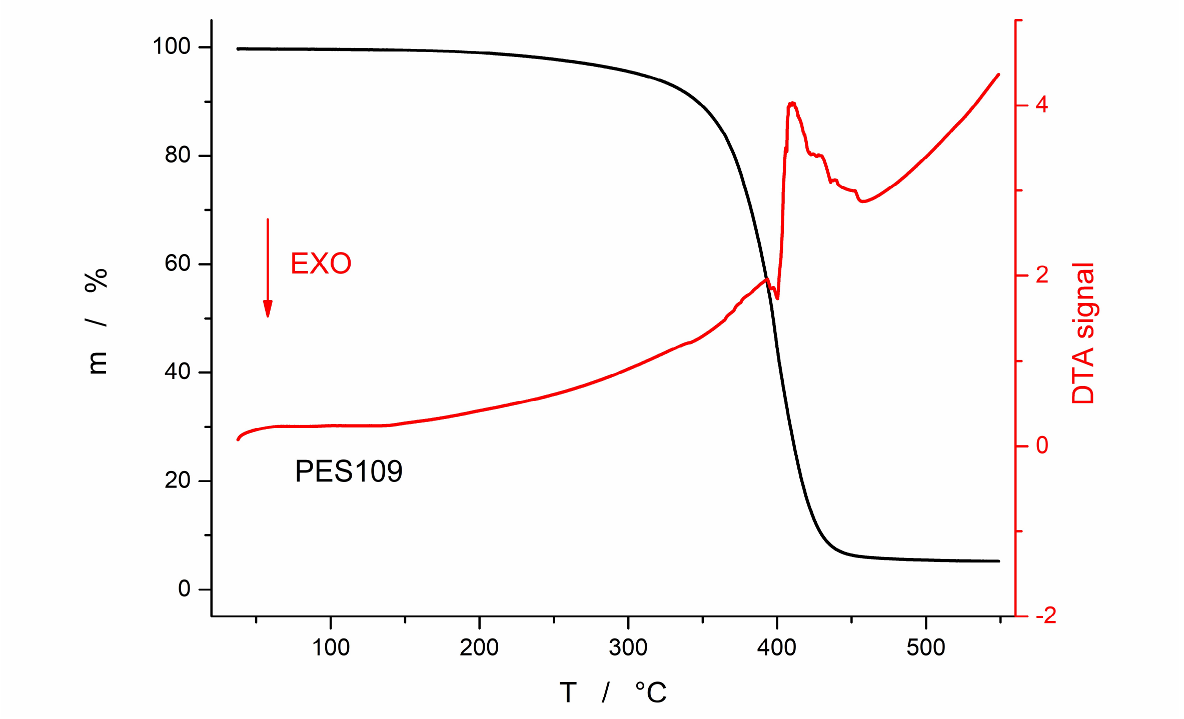

Figure S65. TGA measurement of **PES109** in nitrogen atmosphere.

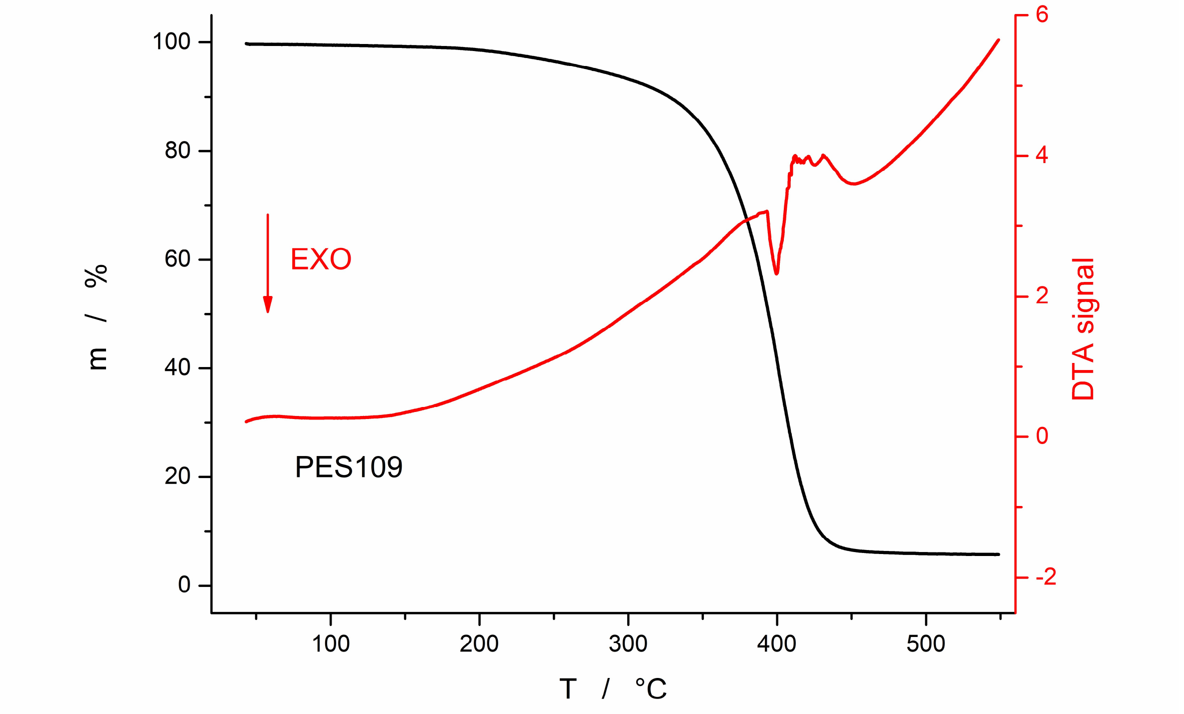

Figure S66. TGA measurement of **PES109** in air atmosphere.

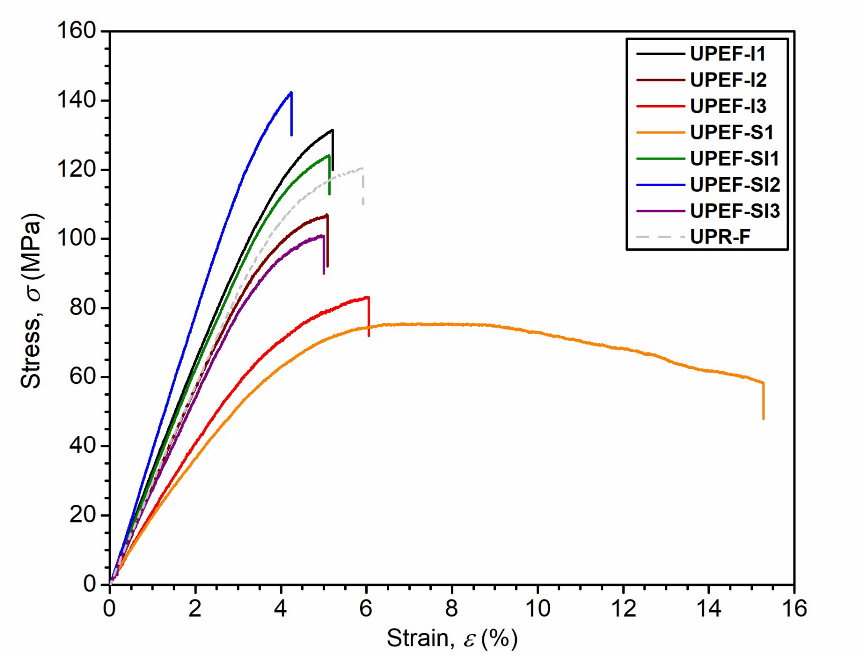

Figure S67. Stress-strain curves of flexural strength test of prepared resins.

Table S1. Full composition of synthesized UPs.[a,b]

Polyester	FDCA	IA	SA	MES [c]	OA [d]	EG	DEG
**UPEF-I1**	0.25 (0.24)	0.25 (0.20)	–	– (0.02)	– (0.01)	0.25 (0.26)	0.25 (0.26)
**UPEF-I2**	0.20 (0.19)	0.30 (0.26)	–	– (0.03)	– (0.01)	0.20 (0.2)	0.30 (0.32)
**UPEF-I3**	0.15 (0.14)	0.35 (0.31)	–	– (0.02)	– (0.01)	0.15 (0.15)	0.35 (0.37)
**UPEF-S1**	0.20 (0.18)	0.20 (0.18)	0.10 (0.10)	– (0.01)	– (0.02)	0.20 (0.20)	0.30 (0.31)
**UPEF-S2**	0.15 (0.13)	0.15 (0.14)	0.20 (0.20)	– (0.01)	– (0.02)	0.15 (0.15)	0.35 (0.36)
**UPEF-SI1**	0.225 (0.22)	0.25 (0.20)	0.025 (0.03)	– (0.02)	– (0.00)	0.225 (0.23)	0.275 (0.30)
**UPEF-SI2**	0.20 (0.19)	0.25 (0.23)	0.05 (0.05)	– (0.01)	– (0.01)	0.20 (0.21)	0.30 (0.30)
**UPEF-SI3**	0.15 (0.15)	0.25 (0.23)	0.10 (0.11)	– (0.01)	– (0.01)	0.15 (0.14)	0.35 (0.34)

[a] Molar content of used building blocks. [b] Feed composition; composition determined by ^{1}H NMR is given in parentheses. [c] Mesaconate. [d] Ordelt adduct.

Table S2. AE calculation for the synthesis of **GLF**.[a]

Component	x	$M_{reactant}$ (g/mol)	y	$M_{product}$ (g/mol)
PEF	1	182		
DEG	1	106		
**GLF**			1	288
	$\Sigma\, x \cdot M_{reactant} = 288$		$\Sigma\, y \cdot M_{product} = 288$	
			**AE = 100%**	

[a] AE parameter was calculated according to Eq. 7.

Table S3. E-factor calculation for the synthesis of **GLF**.[a]

Component	$m_{reactant}$ (g)	$m_{product}$ (g)	m_{waste} (g)
PEF	50		
DEG	29.2		
**GLF**		79.2	
waste			0
		**E-factor = 0**	

[a] AE parameter was calculated according to Eq. 8.

Table S4. AE calculations for the synthesis of **UPEF-I1**.[a]

Component	x	$M_{reactant}$ (g/mol)	y	$M_{product}$ (g/mol)
**GLF**	0.25	288	0.25	270
IA	0.25	130	0.25	112
	$\Sigma\, x \cdot M_{reactant}$ = 104.5		$\Sigma\, y \cdot M_{product}$ = 95.5	
			**AE = 91.4%**	

[a] AE parameter was calculated according to Eq. 7 using stoichiometric coefficients given in Table 1.

Table S5. E-factor calculation for the synthesis of **UPEF-I1**.[a]

Component	$m_{reactant}$ (g)	$m_{product}$ (g)	m_{waste} (g)
**GLF**	20.7		
IA	9.3		
**UPEF-S1**		27.4	
toluene			8.7
water			2.6
		**E-factor = 0.41**	

[a] AE parameter was calculated according to Eq. 8.

Table S6. AE calculations for the synthesis of **UPEF-I2**.[a]

Component	x	$M_{reactant}$ (g/mol)	y	$M_{product}$ (g/mol)
**GLF**	0.2	288	0.2	270
IA	0.3	130	0.3	112
DEG	0.1	106	0.1	88
	$\Sigma\, x \cdot M_{reactant} = 107.2$		$\Sigma\, y \cdot M_{product} = 96.4$	
			**AE = 89.9%**	

[a] AE parameter was calculated according to Eq. 7 using stoichiometric coefficients given in Table 1.

Table S7. E-factor calculation for the synthesis of **UPEF-I2**.[a]

Component	$m_{reactant}$ (g)	$m_{product}$ (g)	m_{waste} (g)
**GLF**	16.1		
IA	10.9		
DEG	3		
**UPEF-S1**		27	
toluene			8.7
water			3
		**E-factor = 0.43**	

[a] AE parameter was calculated according to Eq. 8.

Table S8. AE calculations for the synthesis of **UPEF-I3**.[a]

Component	x	$M_{reactant}$ (g/mol)	y	$M_{product}$ (g/mol)
**GLF**	0.15	288	0.15	270
IA	0.35	130	0.35	112
DEG	0.2	106	0.2	88
	$\Sigma\, x \cdot M_{reactant} = 109.9$		$\Sigma\, y \cdot M_{product} = 97.3$	
			**AE = 88.5%**	

[a] AE parameter was calculated according to Eq. 7 using stoichiometric coefficients given in Table 1.

Table S9. E-factor calculation for the synthesis of **UPEF-I3**.[a]

Component	$m_{reactant}$ (g)	$m_{product}$ (g)	m_{waste} (g)
**GLF**	11.8		
IA	12.4		
DEG	5.8		
**UPEF-S1**		26.6	
toluene			8.7
water			3.4
		**E-factor = 0.45**	

[a] AE parameter was calculated according to Eq. 8.

Table S10. AE calculations for the synthesis of **UPEF-S1**.[a]

Component	x	$M_{reactant}$ (g/mol)	y	$M_{product}$ (g/mol)
**GLF**	0.2	288	0.2	270
IA	0.2	130	0.2	112
SA	0.1	118	0.1	100
DEG	0.1	106	0.1	88
	$\Sigma\, x \cdot M_{reactant} = 106$		$\Sigma\, y \cdot M_{product} = 95.2$	
			**AE = 89.8%**	

[a] AE parameter was calculated according to Eq. 7 using stoichiometric coefficients given in Table 1.

Table S11. E-factor calculation for the synthesis of **UPEF-S1**.[a]

Component	$m_{reactant}$ (g)	$m_{product}$ (g)	m_{waste} (g)
**GLF**	16.3		
IA	7.4		
SA	3.3		
DEG	3		
**UPEF-S1**		26.9	
toluene			8.7
water			3.1
		**E-factor = 0.44**	

[a] AE parameter was calculated according to Eq. 8.

Table S12. AE calculations for the synthesis of **UPEF-S2**.[a]

Component	x	$M_{reactant}$ (g/mol)	y	$M_{product}$ (g/mol)
**GLF**	0.15	288	0.15	270
IA	0.15	130	0.15	112
SA	0.2	118	0.2	100
DEG	0.2	106	0.2	88
	$\Sigma\, x \cdot M_{reactant}$ = 107.5		$\Sigma\, y \cdot M_{product}$ = 94.9	
			**AE = 88.3%**	

[a] AE parameter was calculated according to Eq. 7 using stoichiometric coefficients given in Table 1.

Table S13. E-factor calculation for the synthesis of **UPEF-S2**.[a]

Component	$m_{reactant}$ (g)	$m_{product}$ (g)	m_{waste} (g)
**GLF**	12		
IA	5.4		
SA	6.6		
DEG	5.9		
**UPEF-S1**		26.5	
toluene			8.7
water			3.5
		**E-factor = 0.46**	

[a] AE parameter was calculated according to Eq. 8.

Table S14. AE calculations for the synthesis of **UPEF-SI1**.[a]

Component	x	$M_{reactant}$ (g/mol)	y	$M_{product}$ (g/mol)
**GLF**	0.225	288	0.225	270
IA	0.25	130	0.25	112
SA	0.025	118	0.025	100
DEG	0.05	106	0.05	88
	$\Sigma\, x \cdot M_{reactant}$ = 105.6		$\Sigma\, y \cdot M_{product}$ = 95.7	
			**AE = 90.6%**	

[a] AE parameter was calculated according to Eq. 7 using stoichiometric coefficients given in Table 1.

Table S15. E-factor calculation for the synthesis of **UPEF-SI1**.[a]

Component	$m_{reactant}$ (g)	$m_{product}$ (g)	m_{waste} (g)
**GLF**	18.4		
IA	9.2		
SA	0.8		
DEG	1.5		
**UPEF-S1**		27.2	
toluene			8.7
water			2.8
		**E-factor = 0.42**	

[a] AE parameter was calculated according to Eq. 8.

Table S16. AE calculations for the synthesis of **UPEF-SI2**.[a]

Component	x	$M_{reactant}$ (g/mol)	y	$M_{product}$ (g/mol)
**GLF**	0.2	288	0.2	270
IA	0.25	130	0.25	112
SA	0.05	118	0.05	100
DEG	0.1	106	0.1	88
		$\Sigma\, x \cdot M_{reactant}$ = 106.6		$\Sigma\, y \cdot M_{product}$ = 95.8
				**AE = 89.9%**

[a] AE parameter was calculated according to Eq. 7 using stoichiometric coefficients given in Table 1.

Table S17. E-factor calculation for the synthesis of **UPEF-SI2**.[a]

Component	$m_{reactant}$ (g)	$m_{product}$ (g)	m_{waste} (g)
**GLF**	16.2		
IA	9.1		
SA	1.7		
DEG	3		
**UPEF-S1**		27	
toluene			8.7
water			3
		**E-factor = 0.43**	

[a] AE parameter was calculated according to Eq. 8.

Table S18. AE calculations for the synthesis of **UPEF-SI3**.[a]

Component	x	$M_{reactant}$ (g/mol)	y	$M_{product}$ (g/mol)
**GLF**	0.15	288	0.15	270
IA	0.25	130	0.25	112
SA	0.1	118	0.1	100
DEG	0.2	106	0.2	88
		$\Sigma\ x \cdot M_{reactant}$ = 108.7		$\Sigma\ y \cdot M_{product}$ = 96.1
				**AE = 88.4%**

[a] AE parameter was calculated according to Eq. 7 using stoichiometric coefficients given in Table 1.

Table S19. E-factor calculation for the synthesis of **UPEF-SI3**.[a]

Component	$m_{reactant}$ (g)	$m_{product}$ (g)	m_{waste} (g)
**GLF**	11.9		
IA	9		
SA	3.3		
DEG	5.9		
**UPEF-S1**		26.5	
toluene			8.7
water			3.5
		**E-factor = 0.46**	

[a] AE parameter was calculated according to Eq. 8.

Table S20. AE calculations for the synthesis of **UPEF-SI3**.[a]

Component	x	$M_{reactant}$ (g/mol)	y	$M_{product}$ (g/mol)
PA	1	148	1	148
MA	1	98	1	98
PG	1	76	1	58
EG	1	62	1	44
		$\Sigma\, x \cdot M_{reactant} = 384$		$\Sigma\, y \cdot M_{product} = 348$
				**AE = 90.6%**

[a] AE parameter was calculated according to Eq. 7 using stoichiometric coefficients given in Table 1.

Table S21. E-factor calculation for the synthesis of **UPEF-SI3**.[a]

Component	$m_{reactant}$ (g)	$m_{product}$ (g)	m_{waste} (g)
**GLF**	74		
IA	49		
SA	38		
DEG	31		
**UPEF-S1**		174	
toluene			17.3
water			18
		**E-factor = 0.2**	

[a] AE parameter was calculated according to Eq. 8.
